# Cancer risk and mortality among firefighters: a meta-analytic review

**DOI:** 10.3389/fonc.2023.1130754

**Published:** 2023-05-12

**Authors:** David J. Lee, Soyeon Ahn, Laura A. McClure, Alberto J. Caban-Martinez, Erin N. Kobetz, Henna Ukani, Devina J. Boga, Diana Hernandez, Paulo S. Pinheiro

**Affiliations:** ^1^ Department Public Health Sciences, Leonard M. Miller School of Medicine, University of Miami, Miami, FL, United States; ^2^ Sylvester Comprehensive Cancer Center, University of Miami Health Systems, Miami, FL, United States; ^3^ Department of Educational and Psychological Studies, School of Education and Human Development, University of Miami, Miami, FL, United States; ^4^ Department of Physical Medicine and Rehabilitation, Leonard M. Miller School of Medicine, University of Miami, Miami, FL, United States; ^5^ Department of Medicine, Leonard M. Miller School of Medicine, University of Miami, Miami, FL, United States

**Keywords:** firefighters, meta-analysis, cancer incidence, cancer mortality, occupational research, review

## Abstract

**Background:**

Firefighting is a hazardous occupation that is associated with an increased risk of select cancers. The number of studies has grown in recent years allowing for a synthesis of findings.

**Methods:**

Following PRISMA guidelines, multiple electronic databases were searched to identify studies on firefighter cancer risk and mortality. We computed pooled standardized incidence risk (SIRE) and standardized mortality estimates (SMRE), tested for publication bias, and conducted moderator analyses.

**Results:**

Thirty-eight studies published between 1978 and March 2022 were included for final meta-analysis. Overall, cancer incidence and mortality were significantly lower for firefighters (SIRE = 0.93; 95% CI: 0.91-0.95; SMRE = 0.93; 95% CI: 0.92 - 0.95) compared to the general population. Incident cancer risks were significantly higher for skin melanoma (SIRE = 1.14; 95% CI:1.08 - 1.21), other skin cancers (SIRE = 1.24; 95% CI:1.16-1.32), and prostate cancer (SIRE = 1.09; 95% CI: 1.04-1.14). Firefighters showed higher mortality for rectum (SMRE = 1.18; 95% CI: 1.02-1.36), testis (SMRE = 1.64; 95% CI: 1.00-2.67), and non-Hodgkin lymphoma (SMRE = 1.20; 95% CI: 1.02-1.40). There was evidence of publication bias for SIRE and SMRE estimates. Some moderators explained variations in study effects, including study quality scores.

**Conclusion:**

Firefighters are at higher risk for several cancers; to the extent that some (e.g., melanoma and prostate) are screening amenable, more study into firefighter-specific recommendations for cancer surveillance is needed. Moreover, longitudinal studies with more detailed data on the specific length and types of exposures are necessary, as well as on unstudied subtypes of cancers (e.g., subtypes of brain cancer and leukemias) are needed.

## Introduction

Firefighting is a hazardous occupation that is associated with an increase in the risk of select cancers. Hazards include direct and indirect exposure to known and suspected carcinogens. Firefighters may inhale, ingest, or have skin contact with known carcinogens such as polycyclic aromatic hydrocarbons (PAH) and benzene (1). Exposure to firefighting activities leads to increased urinary levels of a variety of chemicals including PAHs, benzene, organo-chlorine and -phosphorus compounds, phenols, phthalates and heavy metals and metalloids (2). Changes in DNA methylation have been documented in firefighters 2-3 years relative to pre-fire school training levels (3). Select methylated sites with pathways associated with cancer risk were identified providing one potential mechanistic pathway linking occupational exposures to cancer risks in firefighters. The International Agency for Research on Cancer (IARC) is working on a comprehensive update of a previous report issued on cancer risks in firefighters (4). The final report has not yet been released but an initial summary of findings indicates that there is strong mechanistic evidence that occupational exposures documented in firefighters exhibit carcinogenic characteristics (5). On the basis of this comprehensive review the Working Group has concluded that the occupational exposures of firefighters are “carcinogenic to humans (Group 1) based on ‘sufficient’ evidence for cancer in humans”.

Although numerous studies have been conducted, generalizable and comparative assessments are complicated by considerable variations in study design, time period, geographic location, measurement of exposure, definition of firefighter roles, and classification of cancer diagnoses, among other study characteristics. Further complications may arise from small sample sizes and time-related variables such as changes in safety regulations and types of firefighter exposures. Due to these challenges faced by primary researchers, meta-analysis has been used as a valuable tool for understanding the overall relationship between firefighting and cancer and further delineates the differential (or moderating) effects.

However, previous meta-analyses on this topic reveal mixed findings. For example, LeMasters et al. (6) found firefighters are at probable higher risk for multiple myeloma, non-Hodgkin lymphoma, prostate, and testicular cancers. The earlier IARC report on occupational exposures in firefighters included a meta-analysis which found increased risks for prostate, testicular, and non-Hodgkin lymphoma cancers (4). A more recent meta-analysis by Jalilian et al. (7) found firefighters to be at greater risk for colorectal, prostate, testicular, bladder, thyroid, and pleural cancers and malignant melanoma, and increased mortality for rectal cancer and non-Hodgkin lymphoma. Similarly, Soteriades et al. (8) found firefighting to be associated with incident colon, prostate, and testicular cancers. Firefighter mortality was elevated for a larger number of cancer subtypes including brain and central nervous system, non-Hodgkin lymphoma, melanoma, rectal, bladder, prostate, testicular, Hodgkin’s disease, lymphosarcoma and reticulosarcoma, multiple myeloma, pancreatic, and kidney cancer. However, a more restrictive analysis conducted by Soteriades et al. (8) based on a subset of studies judged to be high quality found significantly higher risks only for testicular incident cancers and mortality due to rectal (and colorectal) cancer. Finally, a recent examination of eleven systematic reviews examining firefighter cancer incidence and mortality concluded that relative to the general population, firefighters are at higher risk of bladder cancer, melanoma, mesothelioma, prostate cancer, and rectal cancer (9). Excess mortality due to rectal cancer and non-Hodgkin lymphoma was also consistently reported to be elevated in firefighters. Such large variations in individual studies and mixed findings from previous meta-analyses demonstrated a need for further research.

Additionally, chronological and geographical overlap in participants was found across firefighter cancer studies, demonstrating the dependency issues in study effects when conducting meta-analysis. The Jalilian et al.’s (7) meta-analysis did not take this into consideration while the Soteriades et al.’s (8) meta-analysis did exclude papers which covered similar geographic areas. Included in the Jalilian et al.’s (7) and Soteriades et al.’s (8) meta-analyses were studies which employed less common study designs (e.g., case-control), which when combined with the more common designs, can introduce heterogeneity complicating interpretation of study findings. With our careful inspection of study design, we only included studies that do not have substantial geographical and chronological overlap a potential dependency that would inflate type I error rates. In addition, we performed a variety of moderator analyses such that the sources of the variations in effects (i.e., study quality, gender, type of effect size) can be identified.

Lastly, a number of new studies examining firefighter cancer risk and mortality have been published since the Jalilian et al.’s (7) and Soteriades et al.’s (8) meta-analyses were completed (10–16) which have been incorporated into the present analysis (n=7). Therefore, the present meta-analysis provides an updated review of worldwide cancer risk among firefighters. Details of the protocol for this meta-analysis were registered on PROSPERO (17) and can be accessed at www.crd.york.ac.uk/PROSPERO/display_record.asp?ID=CRD42019110520.

## Materials and methods

### Search strategy

Relevant studies were located by searching multiple sources. First, a series of comprehensive electronic searches were conducted using multiple databases including ERIC, PsychINFO, ProQuest Dissertation & Theses, PUBMED, and MEDLINE *via* EBSCO. Second, we performed citation searches using various online search engines including Embase, Web of Science Core collection, Google Scholar, and SCOPUS. Third, we searched multiple websites including government, cancer registries, and the Cochrane Library. Keywords being used in all our searches are a combination of the following terms: (Cancer OR tumor OR malignancy OR neoplasm OR mutation) & (fire inspector OR fire inspectors OR fire rescue OR fire-rescue OR firefighter OR firefighters OR “fire fighter” OR “fire fighters” OR paramedic OR paramedics OR emergency medical technician OR “first responder”). All Mesh terms used in our searches can be found in Appendix 1. Two independent reviewers were responsible for determining if studies were eligible with a third author confirming any discrepancies in whether a study met eligibility criteria.

### Inclusion and exclusion criteria

To be included in the current meta-analysis, a study must meet the following inclusion and exclusion criteria, including:

1. A study must be empirical and quantitative;
2. A study must be based on human research;
3. A study population must be firefighters;
4. A study must be related to firefighters’ cancer incidence or mortality;
5. A study must be written in English;
6. A study must provide cancer incidence and/or mortality from firefighters that are largely geographically and chronologically independent from those included other studies;
7. The study comparison group must be the general population (e.g., multi-national, national, regional, or local)
8. A study must report sufficient statistics that enable us to compute effect size and its associated standard error.

**Figure 1:**
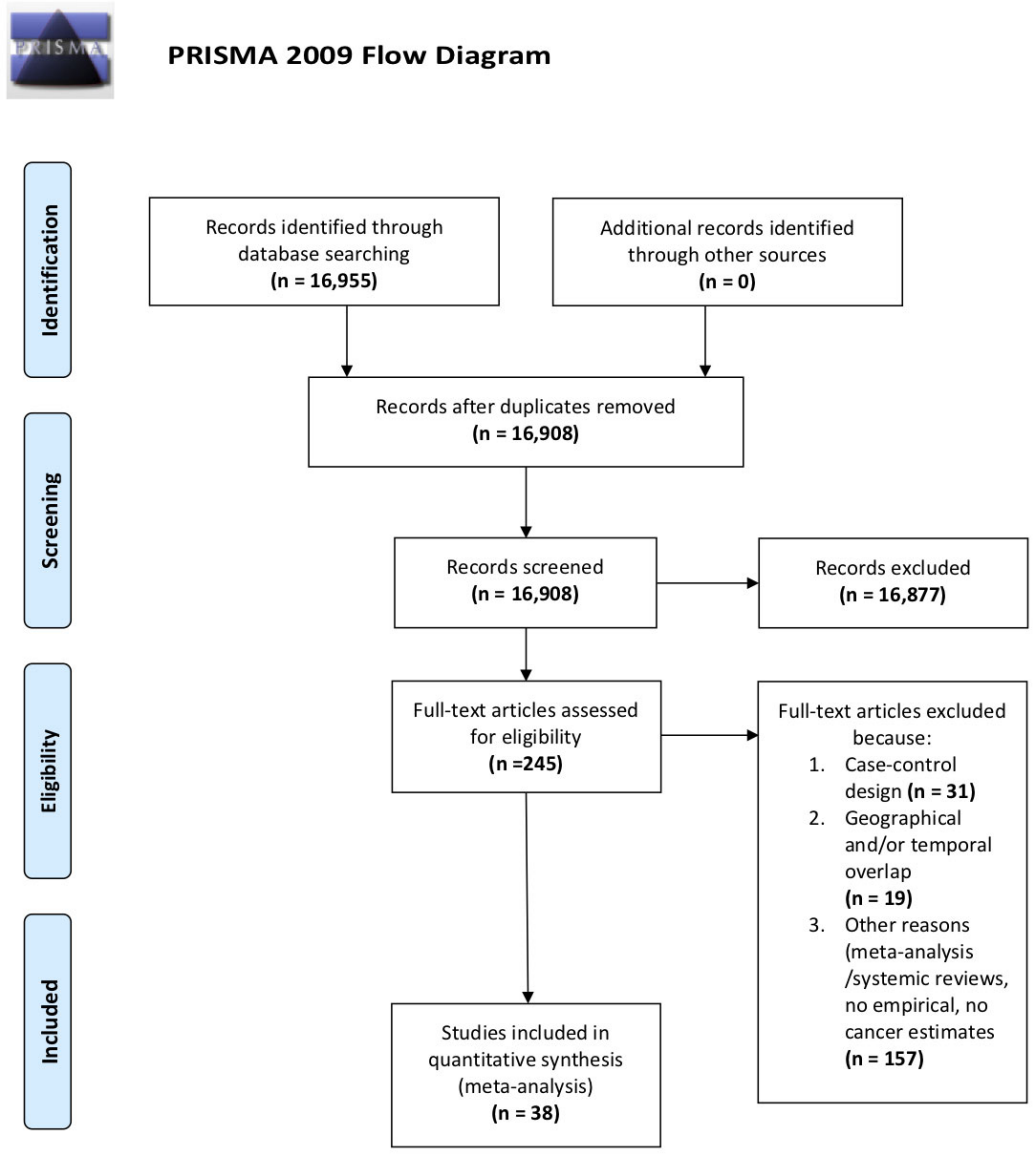
Flow diagram.

**Figure 1** displays a PRISMA flow chart that summarizes the comprehensive search process based on inclusion and exclusion criteria.

### Coding of study characteristics

Each study initially considered for the present meta-analysis was reviewed and coded, including (1) design characteristics (i.e., cohort, cross-sectional, longitudinal, mixed, and other) (2), outcome type (i.e., incidence and mortality) (3), cancer coding system (i.e., International Classification of Diseases (ICD) ICD-8, ICD-9, ICD-10, International Classification of Disease for Oncology (ICD-O), ICD-O-2, ICD-O-3, Surveillance, Epidemiology, End Results (SEER) codes, and others) (4), cancer sites (5), whether *in situ* cases were excluded in data analyses (6), source of occupation designations (i.e., employment, certification, cancer, registry, death certificate, and other) (7), type of incident that firefighters attended (8), sample characteristics (i.e., age, employment duration, employment status, gender, race/ethnicity, and smoking status), and (9) study characteristics (i.e., location, publication type, and publication year). The two coders independently read the included studies and extracted study information from the 38 studies included in the meta-analysis based on the final coding instrument through REDCap (see Appendix 2 for REDCap coding sheet). Intraclass correlation (ICC) calculated for each category indicated good to excellent level of inter-coder reliability, yielding a range from .73 (type of research design) to 1.00 (study location). Any discrepancies discovered in the coding stage were corrected based on the additional review by the first author before data analysis.

### Effect sizes and standard errors

The primary effect size measures used in the current meta-analysis were (1) standardized incidence ratio (SIR) and (2) standardized mortality ratio (SMR) depending on study designs used in the primary studies. We calculated pooled incidence risk estimates (SIRE) and mortality risk estimates (SMRE) as described below.

First, the reported SIR value was directly extracted from each study. When it was not reported, the SIR value was computed by dividing the observed number of firefighters with cancer by the expected number of the reference population with cancer. The computed SIR value was transformed to its logarithm by taking the log of the computed SIR value, whose standard error value was computed by taking a square root of 1/E1 + 1/E2, where E1 and E2 are the expected frequencies of firefighters and reference population with cancer. If only 95% confidence intervals around the reported SIR value are given, the standard error was computed using: (UL – SIR)/1.96, where UL is an upper level of 95% confidence interval and SIR is the reported SIR value.

Second, the reported SMR value and its associated standard error were directly extracted from each study. When it was not reported, the SMR value was computed by dividing the observed number of firefighters’ mortality due to cancer by the expected number of the reference population’s mortality due to cancer. The computed SMR value was transformed to its logarithm by taking the log of the computed SMR value, whose standard error value was computed by taking a square root of 1/O, where O is the observed number of firefighters’ mortality due to cancer. If only 95% confidence intervals around the reported SMR value are given, the standard error was computed using: (UL – SMR)/1.96, where UL is an upper level of 95% confidence interval and SMR is the reported SMR value.

All the parameter estimates (i.e., pooled SIRE and SMRE) computed in the meta-analysis were transformed back to the original scale by taking exponential (*e*) to the power of the estimates.

### Publication bias

The potential for publication bias, which might occur due to the possibility that studies demonstrating a significant effect in a favorable direction are more likely to be published, was assessed using multiple indicators. These included (1): Begg and Mazumdar’s rank correlation test (18) for funnel plot asymmetry (2), Egger’s regression test of intercept (19), and (3) funnel plots. When the null hypothesis stating no relationship between effect sizes and their associated precision measures is accepted, we can conclude that there is no sufficient evidence supporting publication bias in the included studies.

### Handling dependency in effect sizes

As many studies reported effect sizes in relation to multiple cancer sites, more than one effect sizes were extracted from a single study, leading to the violation of independence assumption for the meta-analysis (20). Before any analysis, we first excluded studies that were geographically overlapping with others. Of several available methods that deal with dependency issues (21), effect sizes were first separated by outcome type (i.e., SMR vs. SIR). We performed statistical analysis separated by type of outcome variables. Second, within each cancer site, we checked whether effect sizes from the same study are independent of one another. When effect sizes were based on independent samples (i.e., effects reported separated by gender (22), firefighters exposed to 9/11 vs. those not exposed to 9/11 (23), FDNY vs. CFHS (15), low, medium, vs. high risk (24), they were treated as independent effects.

### Study quality assessment

Two content experts (first and last authors) independently rated 38 studies using the Risk of Bias and Precision of Observational Studies (RTI) item banks and Newcastle-Ottawa Quality Assessment Scale (Newcastle-Ottawa). These scales were examined by the independent reviewers on a sample of studies not included in the final analysis to ensure consistent assumptions and criteria were employed. Slight modifications were then made to the original quality assessments to better align with the methods of the studies evaluated, and some items were removed that were not relevant. Then, the Many-facet Rasch Measurement Model (MFRM) was used to estimate the latent quality score of each assessment, which is expressed on a standard score (z-score) with a mean of 0 (25).

### Statistical analysis

The metaphor package (26) in R version 4.0.2 (R Development Core Team, 2021) was used to analyze the data, which was based on the meta-analytic methods proposed by Hedges and Olkin (27). First, the overall homogeneity was assessed using *Q_total_* under the assumption that all total number of effects (*k*) were from the sample population. If *Q_total_* was found to be statistically significant, the overall effect was estimated under the random-effects model, where between-study variance was estimated using Restricted Maximum Likelihood (REML) estimation method (28). Otherwise, the fixed-effects model was used to estimate the overall effect (i.e., pooled SIRE and SMRE). All main analyses were performed separately by cancer sites. Second, when *Q_total_* was found to be significant, a series of moderator analyses with a categorical predictor (i.e., weighted Analysis of Variance [ANOVA]) were performed to identify source of variability in SIRs or SMRs. In particular, the mixed-effects model with a categorical predictor was adopted when a within-study variation (*Q_within_*) is found to be statistically significant after controlling for the moderator. When the mixed-effects model was used, the additional between-study variance after controlling for moderator was estimated using the REML method and then incorporated. Otherwise, the fixed-effects analysis with a categorical predictor was performed. In these moderator analyses, the significant *Q_model_* suggests that study effects (i.e., SIRs or SMRs) significantly differ depending on subgroups. Moderators used in the current meta-analysis include (1): cancer sites (2), whether *in situ* cases were excluded in data analyses (3), participant characteristics (i.e., employment status, gender, race/ethnicity, and smoking status), and (4) study characteristics (i.e., location, publication type). More details about random-effects or mixed-effects model can be found in Raudenbush (28).

## Results

### Description of studies

A total of 38 independent studies published between 1978 and 2022 were coded in a number of study characteristics including (1): outcome type (i.e., *k _study_* = 17, reporting SIR, *k _study_* = 26, reporting SMR; with 5 studies reporting both SIR and SMR) (2), cancer coding system (3), cancer sites (4), whether *in situ* cases were excluded in data analyses (*k_study_* = 1 *in situ* cases were included, *k_study_* = 3 *in situ* cases were excluded, *k_study_* = 34 not reported) (5), source of occupation designations (i.e., employment, certification, cancer, registry, death certificate, and other) (6), type of incident that firefighters attended (e.g., 9-11) (7), participant characteristics (e.g., age, employment status, gender, race/ethnicity, and smoking status), and (8) study characteristics (i.e., location [*k_study_* = 13 for US studies, *k_study_* = 25 for non-US studies], publication type [*k_study_* = 2 for unpublished, *k_study_* = 36 for published]).

### Assessing publication bias

**Figure 2:**
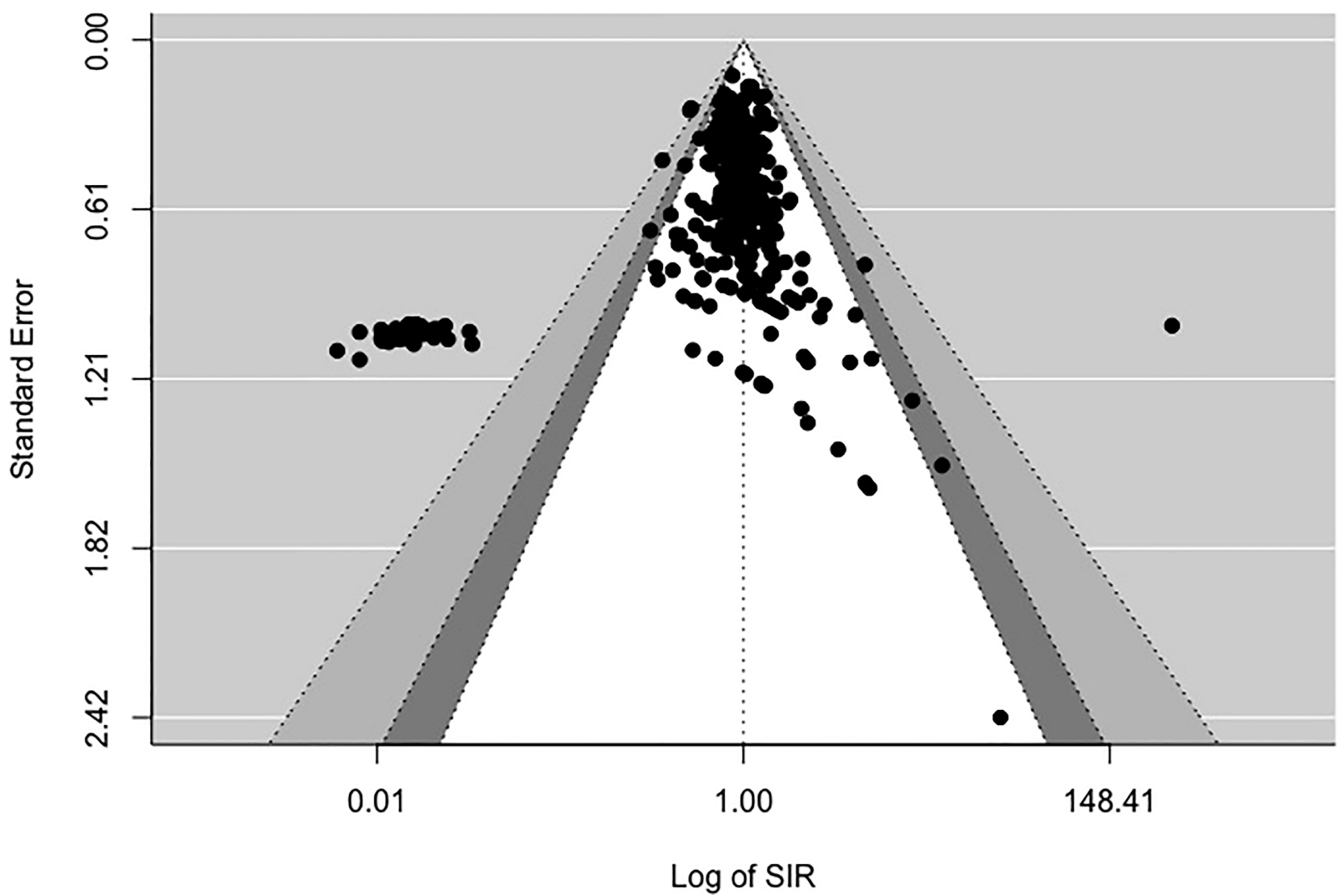
Funnel plot for Standardized Incidence Rate (SIR).

**Figure 3:**
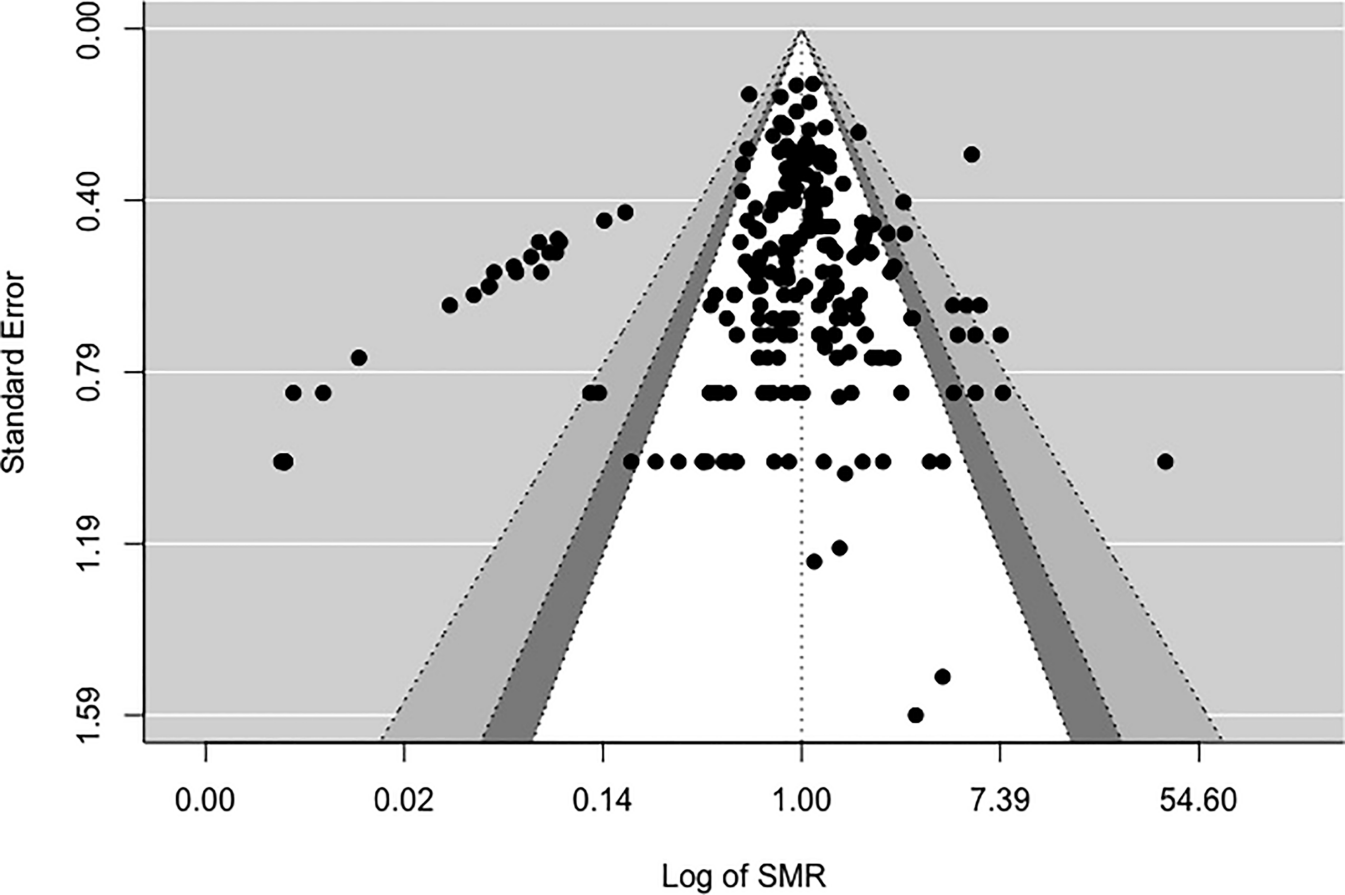
Funnel plot for Standardized Mortality Rate (SMR).

**Figures 2**, **3** display funnel plots that visually display the relationship between effect sizes (SIRE for **Figure 2** and SMRE for **Figure 3**) and their associated standard errors as measure of precision. Results from (1) Begg and Mazumdar’s rank correlation test for funnel plot asymmetry (2), Egger’s regression test of intercept, and (3) funnel plot suggest that there might be some evidence for publication bias for SIRE (*z* = -13.07, *p* <.01 by Egger’s regression test; Kendall’s tau = -.21, *p* <.01) and SMR (*z* = -13.11, *p* <.01 by Egger’s regression test; Kendall’s tau = .03, *p* = .52). Therefore, we have some evidence supporting the potential publication bias in the included studies for both SIRE and SMRE.

### Overall analyses

#### Standardized incidence risk estimates

**Figure 4:**
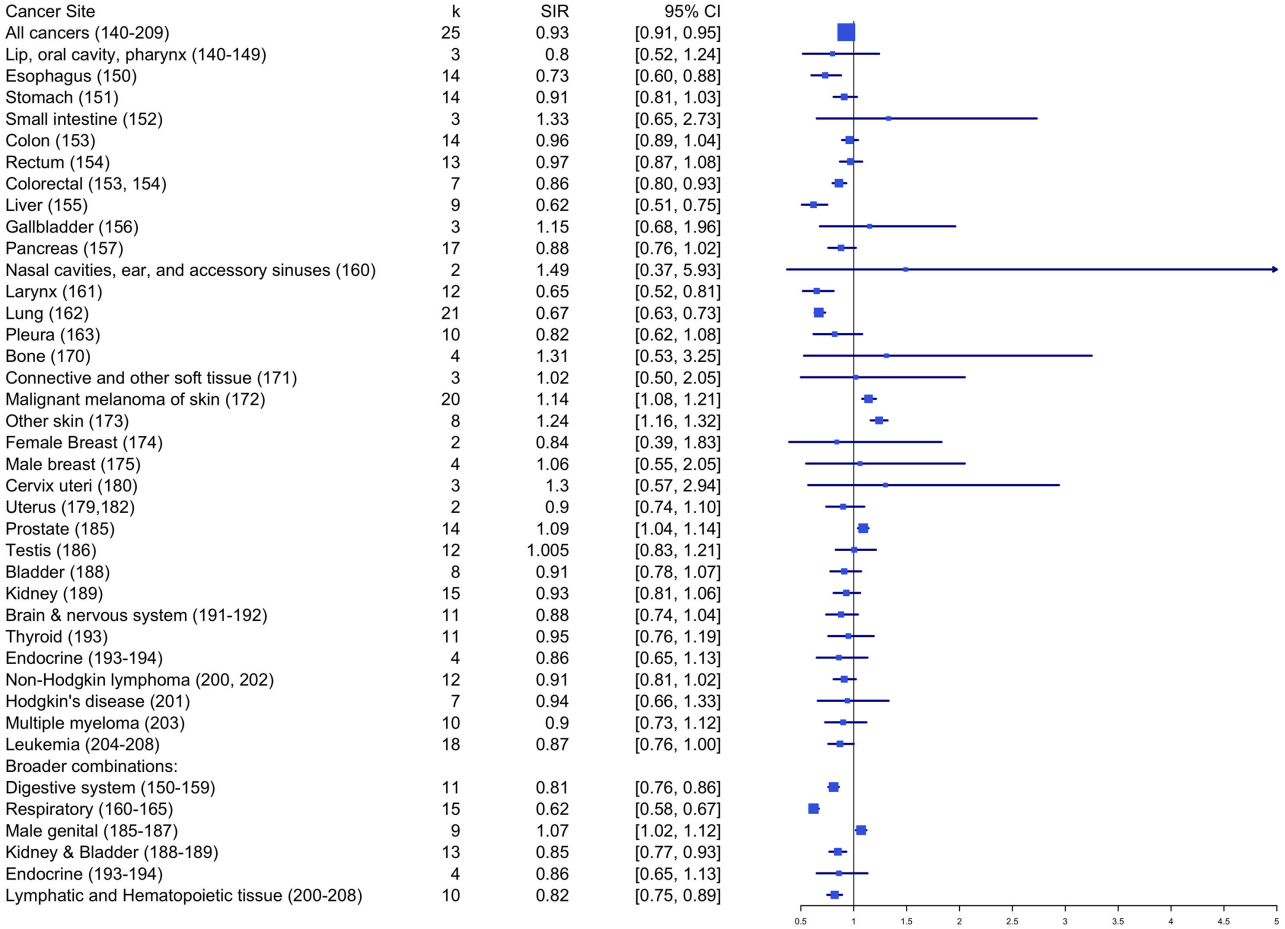
Standardized incidence ratio by cancer site.

**Table 1** and **Figure 4** summarize the pooled effects of standardized incidence risk estimates by cancer site. Results showed that firefighters had significantly higher incidence rates for skin melanoma (SIRE = 1.14, *k* = 20, 95% CI: 1.08 - 1.21), other skin cancers (SIRE = 1.24, *k* = 8, 95% CI: 1.16 – 1.32), and prostate cancer (SIRE = 1.09, *k* = 14, 95% CI: 1.04 - 1.14) when compared to the reference population. However, no significant differences in standardized incidence rates were found for other cancer sites between firefighters and the reference population. Also, incidence rates were significantly lower among firefighters for esophagus (SIRE = 0.73, *k* = 14, 95% CI: 0.60 – 0.88), liver (SIRE = 0.62, *k* = 9, 95% CI: 0.51 – 0.75), larynx (SIRE = 0.65, *k* = 12, 95% CI: 0.52 – 0.81), lung (SIRE = 0.67, *k* = 21, 95% CI: 0.63 – 0.73), colorectal (SIRE = 0.86, *k* = 7, 95% CI: 0.80 – 0.93), lymphatic and hematopoietic tissue (SIRE = 0.82, *k* = 10, 95% CI: 0.75 – 0.89), and all cancers (SIRE = 0.93, *k* = 25, 95% CI: 0.91 – 0.95). No other significant differences in standardized incidence rates were found for other areas of cancer site between firefighters and reference groups.

**Table 1:** Standardized incidence ratio by cancer site.

Cancer sites	SIRE	*k*	95%CI
All cancers (140–209)	0.93**	25	[0.91, 0.95]
Lip, oral cavity, pharynx (140–149)	0.80	3	[0.52, 1.24]
Esophagus (150)	0.73**	14	[0.60, 0.88]
Stomach (151)	0.91	14	[0.81, 1.03]
Small intestine (152)	1.33	3	[0.65, 2.73]
Colon (153)	0.96	14	[0.89, 1.04]
Rectum (154)	0.97	13	[0.87, 1.08]
Colorectal (153, 154)	0.86**	7	[0.80, 0.93]
Liver (155)	0.62**	9	[0.51, 0.75]
Gallbladder (156)	1.15	3	[0.68, 1.96]
Pancreas (157)	0.88	17	[0.76, 1.02]
Nasal cavities, ear, and accessory sinuses (160)	1.49	2	[0.37, 5.93]
Larynx (161)	0.65**	12	[0.52, 0.81]
Lung (162)	0.67**	21	[0.63, 0.73]
Pleura (163)	0.82	10	[0.62, 1.08]
Bone (170)	1.31	4	[0.53, 3.25]
Connective and other soft tissue (171)	1.02	3	[0.50, 2.05]
Malignant melanoma of skin (172)	1.14**	20	[1.08, 1.21]
Other skin (173)	1.24**	8	[1.16, 1.32]
Female Breast (174)	>0.84	2	[0.39, 1.83]
Male breast (175)	1.06	4	[0.55, 2.05]
Cervix uteri (180)	1.30	3	[0.57, 2.94]
Uterus (179,182)	0.90	2	[0.74, 1.10]
Prostate (185)	1.09**	14	[1.04, 1.14]
Testis (186)	1.01	12	[0.83, 1.21]
Bladder (188)	0.91	8	[0.78, 1.07]
Kidney (189)	0.93	15	[0.81, 1.06]
Brain & nervous system (191-192)	0.88	11	[0.74, 1.04]
Thyroid (193)	0.95	11	[0.76, 1.19]
Endocrine (193-194)	0.86	4	[0.65, 1.13]
Non-Hodgkin lymphoma (200, 202)	0.91	12	[0.81, 1.02]
Hodgkin’s disease (201)	0.94	7	[0.66, 1.33]
Multiple myeloma (203)	0.90	10	[0.73, 1.12]
Leukemia (204-208)	0.87	18	[0.76, 1.00]
Broader combinations:			
Digestive system (150-159)	0.81**	11	[0.76, 0.86]
Respiratory (160-165)	0.62**	15	[0.58, 0.67]
Male genital (185-187)	1.07**	9	[1.02, 1.12]
Kidney & Bladder (188-189)	0.85**	13	[0.77, 0.93]
Endocrine (193-194)	0.86	4	[0.65, 1.13]
Lymphatic and Hematopoietic Tissue (200-208)	0.82**	10	[0.75, 0.89]

k = # of effect sizes; **p <.01.

#### Standardized mortality risk estimates

**Figure 5:**
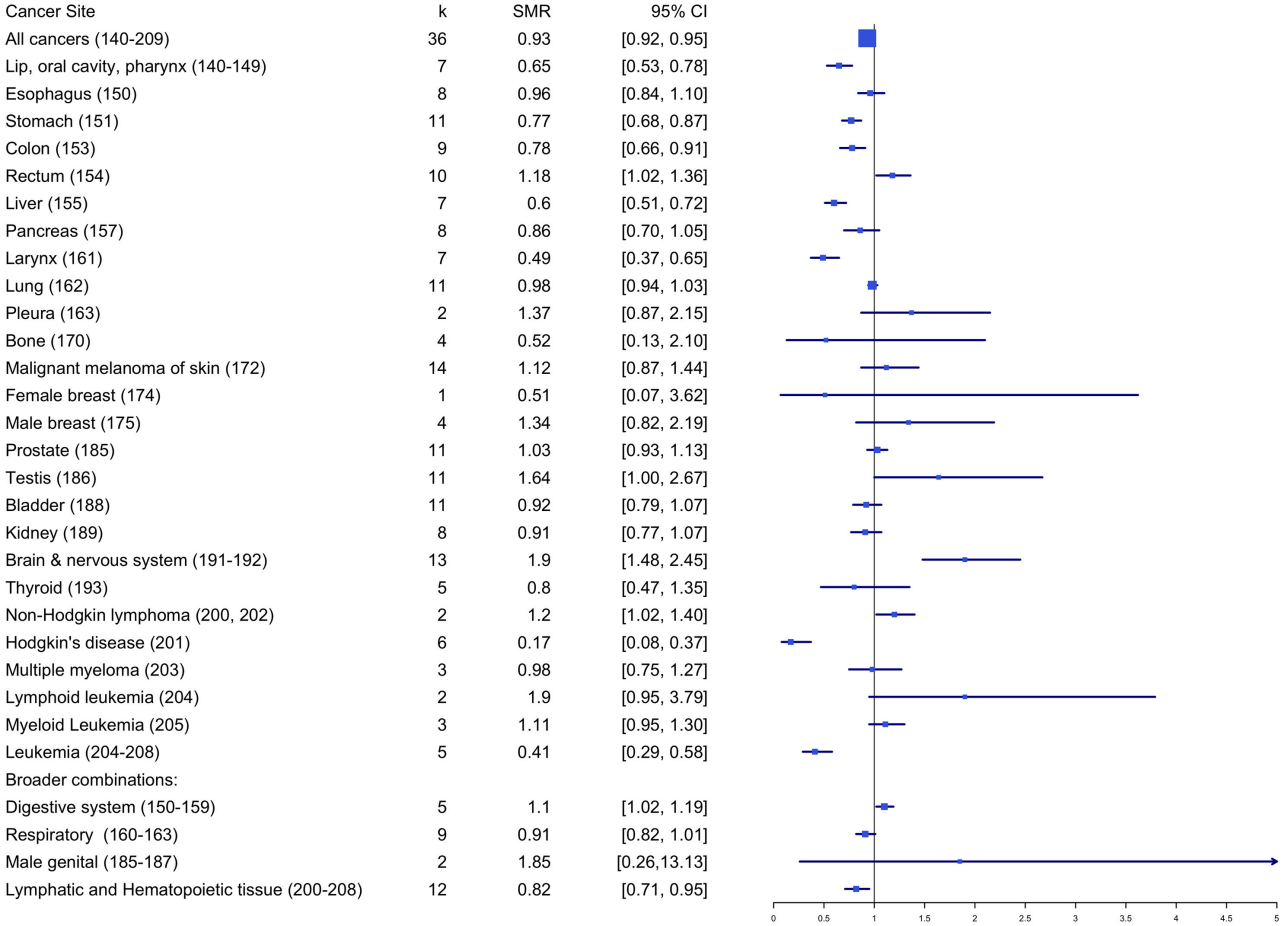
Standardized mortality ratio by cancer site.

**Table 2** and **Figure 5** summarize the estimated overall effects of SMR by cancer site. Results showed firefighters with significantly higher mortality rates for rectum (SMRE = 1.18, *k* = 10, 95% CI: 1.02-1.36), testis (SMRE = 1.64, *k* = 11, 95% CI: 1.00-2.67) and non-Hodgkin lymphoma (SMRE = 1.20, *k* = 2, 95% CI: 1.02-1.40), when compared to the reference population. Also, mortality rates were significantly lower among firefighters for all cancers (SMRE = 0.93, *k* = 36, 95% CI: 0.92-0.95), stomach (SMRE = 0.77, *k* = 11, 95% CI: 0.68-0.87), colon (SMRE = 0.78, *k* = 9, 95% CI: 0.66-0.91), liver (SMRE = 0.60, *k* = 7, 95% CI: 0.51-0.72), larynx (SMRE = 0.49, *k* = 7, 95% CI: 0.37-0.65), bone (SMRE = 0.52, *k* = 2, 95% CI: 0.13-2.10), brain (SMRE = 0.64, *k* = 4, 95% CI: 0.53-0.78), Hodgkin’s disease (SMRE = 0.17, *k* = 6, 95% CI: 0.08-0.37), lip, oral cavity, and pharynx (SMRE = 0.65, *k* = 7, 95% CI: 0.53-0.78), lymphatic and hematopoietic tissue (SMRE = 0.82, *k* = 12, 95% CI: 0.71-0.95). However, no significant differences in standardized mortality rates were found for other cancer sites between firefighters and reference groups.

**Table 2:** Standardized mortality ratio by cancer site.

Cancer site	SMRE	k	95%CI
All cancers (140-209)	0.93**	36	[0.92, 0.95]
Lip, oral cavity, pharynx (140-149)	0.65**	7	[0.53, 0.78]
Esophagus (150)	0.96	8	[0.84, 1.10]
Stomach (151)	0.77**	11	[0.68, 0.87]
Colon (153)	0.78**	9	[0.66, 0.91]
Rectum (154)	1.18**	10	[1.02, 1.36]
Liver (155)	0.60**	7	[0.51, 0.72]
Pancreas (157)	0.86	8	[0.70, 1.05]
Larynx (161)	0.49**	7	[0.37, 0.65]
Lung (162)	0.98	11	[0.94, 1.03]
Pleura (163)	1.37	2	[0.87, 2.15]
Bone (170)	0.52	4	[0.13, 2.10]
Malignant melanoma of skin (172)	1.12	14	[0.87, 1.44]
Male breast (175)	1.34	4	[0.82, 2.19]
Prostate (185)	1.03	11	[0.93, 1.13]
Testis (186)	1.64**	11	[1.00, 2.67]
Bladder (188)	0.92	11	[0.79, 1.07]
Kidney (189)	0.91	8	[0.77, 1.07]
Brain & nervous system (191-192)	1.90**	13	[1.48, 2.45]
Thyroid (193)	0.8	5	[0.47, 1.35]
Non-Hodgkin lymphoma (200, 202)	1.20**	2	[1.02, 1.40]
Hodgkin’s disease (201)	0.17**	6	[0.08, 0.37]
Multiple myeloma (203)	0.98	3	[0.75, 1.27]
Lymphoid leukemia (204)	1.90	2	[0.95, 3.79]
Myeloid Leukemia (205)	1.11	3	[0.95, 1.30]
Leukemia (204-208)	0.41**	5	[0.29, 0.58]
Broader combinations:			
Digestive system (150-159)	1.10**	5	[1.02, 1.19]
Respiratory (160-163)	0.91	9	[0.82, 1.01]
Male genital (185-187)	1.85	2	[0.26,13.13]
Lymphatic and Hematopoietic Tissue (200-208)	0.82**	12	[0.71, 0.95]

k = # of effect size; **p <.01.

### Moderator analyses

#### Standardized incidence risk estimates

Results from the mixed-effects model suggest that several moderators were found to explain variations in SIREs. Significant moderators include: 1) quality score rated using Newcastle-Ottawa Quality Assessment Scale (*Q_model_* (1) = 27.91, *p* <.01), 2) whether studies included female participants or not (*Q_model_* (1) = 19.61, *p* <.01), 3) whether patients with non-malignant tumors were included or not (*Q_model_* (1) = 37.61, *p* <.01). First, the pooled estimate of SIRE was found to be significantly increased by 29% when quality score rated using Newcastle-Ottawa Quality Assessment Scale was increased by 1 (*b* = 1.29, *p* <.01). Second, the pooled SIRE for males was significantly lower than those for females (SIRE = 0.61, *p* = .05 for male firefighters vs. SIRE = 1.07, *p* <.01 for female firefighters). Third, SIRE was significantly lower when participants with non-malignant tumors (e.g., benign brain tumors and *in situ*) were included (SIRE = 0.97, *p* = .70).

#### Standardized mortality risk estimates

Results from the mixed-effects model suggest that several moderators were found to explain variations in SMREs. Significant moderators include: 1) quality score rated using Newcastle-Ottawa Quality Assessment Scale (*Q_model_* (1) = 66.70, *p* <.01), 2) whether studies included female participants or not (*Q_model_* (1) = 11.58, *p* <.01) and 3) study location (*Q_model_* (1) = 11.16, *p* <.01). First, the pooled estimate of SMRE was found to be significantly increased by 55% when quality score rated using Newcastle-Ottawa Quality Assessment Scale was increased (*b* = 1.55, *p* <.01). Second, the pooled SMRE for males was significantly higher than those for females when compared to general population (SMRE = 1.48, *p* = .05 for male firefighters vs. SMRE = 0.71, *p* <.01 for female firefighters). Third, the pooled SMRE extracted from non-US studies (SMRE = 1.10, *p* <.01) was significantly higher than those from US studies (SMRE = 0.65, *p* = .43).

## Discussion

### Summary of study findings

Overall, this meta-analysis demonstrated that the risk of incident cancers was significantly higher for skin melanoma, other skin cancers, prostate cancer, with higher mortality for rectal, testicular, brain and nervous system cancers, and non-Hodgkin lymphoma, among firefighters when compared to reference populations. Below we discuss in greater detail these significant findings including the possible firefighting exposures which could be implicated in these increased risks.

#### Skin cancers

In the present meta-analysis, firefighters had a 14% higher risk of melanoma compared to the general population. A similar pattern was found for mortality, although not statistically significant (SMRE = 1.12, 95% CI: 0.87, 1.44). Other skin cancer incidence rate was also significantly higher in firefighters (SIRE = 1.24, 95% CI: 1.16 – 1.32). Firefighters are exposed to a number of carcinogens associated with melanoma and/or other skin cancers including polycyclic aromatic hydrocarbons (PAHs), Polychlorinated Biphenyls (PCBs), and benzene (29). Often working outside firefighters are exposed to ultraviolent radiation, a known risk factor for melanoma (30). As reviewed previously by Guidotti et al., there may be a causal association between firefighting exposures and risk of melanoma in firefighters although non-occupational ultraviolent radiation and firefighter lifestyle factors cannot be ruled out as a contributing factor (31).

#### Prostate cancer

In the present meta-analysis, incident prostate cancer was significantly higher in firefighters (SIRE = 1.09, 95% CI: 1.04 – 1.14) while mortality was similar to that of the general population (SMRE = 1.03, 95% CI: 0.93 – 1.13). Of note, studies in other populations have documented associations with PAH, PCB, and heavy metal exposures with prostate cancer risk (32). Moreover, shiftwork has been associated with prostate cancer risk in other worker groups (33). Additional research is needed to determine if firefighter-specific carcinogenic exposures can be causally linked to any increased risks for prostate cancer that are independent of possible detection bias due to the different prevalence of PSA testing in firefighters versus other populations.

#### Rectal cancer

In the present meta-analysis, incident rectal cancer was not significantly higher in firefighters (SIRE = 0.97, 95% CI: 0.87 – 1.08) while there was significantly increased risk of death due to rectal cancer (SMRE = 1.18, 95% CI: 1.02 – 1.36). Other investigators have suggested that there is a possible association between rectal cancer mortality and firefighting (6, 34), however, the lack of known carcinogenic exposures linked to this cancer in firefighters should be considered when assessing causality.

#### Testicular cancer

In the present meta-analysis, incident testicular cancer was not significantly higher in firefighters (SIRE = 0.97, 95% CI: 0.87 – 1.08) while there was a significantly higher risk of death due to testicular cancer (SMRE = 1.64, 95% CI: 1.02- 2.67). Firefighters are exposed to Polychlorinated Biphenyls and Polybrominated Biphenyls (PCBs) (4), which is an IARC- verified carcinogen (35); however, studies conducted in other occupational groups are limited (36–38). Caution is required to interpret our finding given the considerable discrepancy in the pooled effects for incidence and mortality.

#### Non-hodgkin lymphoma

The present meta-analysis suggests lower risk (albeit non-significant) for non-Hodgkin lymphoma among firefighters (SIRE = 0.91, 95% CI: 0.81 – 1.02) while a significant increased risk of death was observed on the basis of just two pooled studies (SMRE = 1.20, 95% CI: 1.02 – 1.40). Again, some caution is required to interpret our findings given discrepancies in the pooled effects for incidence and mortality. There is “limited” evidence from studies of non-Hodgkin lymphoma risk to support the Group 1 designation (5), in line with conclusions from IARC (4) and supported by a subsequent meta-analysis completed by LeMasters et al. (6), conducted in approximately the same time period. Of note, non-Hodgkin lymphoma has many distinct sub-types (>30), not all of which have been shown to associated with occupational exposures (39, 40). According to the IARC, exposure to formaldehyde, a known firefighter exposure (4), as well as lindane and pentachlorophenol, both of which can be used to treat wood products, are associated with an increased risk of non-Hodgkin lymphoma (41).

#### Brain and central nervous system cancers

In the present meta-analysis incident brain and central nervous system cancers were not significantly higher in firefighters (SIRE = 0.88, 95% CI: 0.74 – 1.04) while there was a significant increased risk of death due to this heterogeneous group of cancers (SMRE = 1.90, 95% CI: 1.48 – 2.45). This cancer grouping is characterized by a large number of histologically distinct subtypes, only a subset of which may be associated with firefighting-related occupational exposures (39, 42). Lead exposures are associated with firefighting activities (4), and associations between lead exposure and increased brain cancer risk have been documented in other occupational groups as well (43–47).

### The role of moderator analyses in the interpretation of meta-analytic findings

Previous meta-analytic studies of firefighter cancer risk either did not complete formal moderator analyses or conducted analysis with a limited number of variables. Uncovering sources of variability in study effects (i.e., cancer incidence or mortality) can be useful for researchers in that it will enhance the interpretability and generalizability of meta-analytic results, which is estimated based on more consistent study effects. In this meta-analysis, we identified several moderators that explained considerable amount of between-study variability in effects. Jalilian et al.’s documented evidence of moderate to strong heterogeneity for several site-specific cancers including buccal cavity and pharynx, brain and nervous system, esophagus, larynx, lung, melanoma, skin, prostate, and kidney (7).

Not only have we expanded the number of moderator variables examined, but also, we undertook a comprehensive assessment of study quality as a moderator using two quality assessment tools. Soteriades et al.’s meta-analysis included a smaller number of studies and did analyses categorized by level of study quality (i.e., “good and adequate” and “good studies only”). Such subgrouping presumably reduced heterogeneity in effects, but at the expense of the number of studies included in their subgroup analyses across specific cancer sites. For analyses based on “good studies”, the number of studies ranged from 1 to 14 for cancer incidence, and from 2 to 24 for cancer mortality. We found that study quality ratings using the Newcastle-Ottawa Quality Assessment Scale explained variations in SIRE and SMRE estimates, suggesting that SIRE and SMRE estimates are likely to be higher as study quality score is increased. Careful consideration of optimal future study designs examining firefighter cancer risk is needed to capture risk levels more accurately in this occupational group.

The identification of other important moderators helps researchers to give careful consideration to the optimal design of future firefighter cancer studies (i.e., sample characteristics, design characteristics) as well as help to contextualize the present meta-analysis findings. For instance, our moderator analyses also found significantly lower pooled cancer incidence effects for males than females, while pooled cancer mortality was higher for males than females, when compared to general population. One possible explanation for these differential effects is that the proportionately smaller number of female firefighters, when included in select individual studies, resulted in large, but unstable effect size estimates in incidence studies, while lower pooled effects in female mortality studies could reflect differences in aggregate lifetime carcinogenic exposures since women started joining the workforce only in recent decades in the US and elsewhere (48). Caution is therefore warranted when interpreting possible cancer risks in female versus male firefighters given the relative sparseness of the number of observed incident and mortality cases in females as well as differences in the likely average career carcinogenic exposure profiles experienced in male and female fighters in the studies included in the present meta-analysis.

### Comparison with previous meta-analytic findings

Two meta-analyses conducted by independent research teams were published in 2019 (7, 8). There was substantial overlap in the papers selected for these two meta-analyses which, in the case of Jalilian et al. included studies published through 2017. However, Soteriades et al. limited their original searches for articles to papers published between 1960 and 2007, and ultimately did not include any firefighter’s cancer studies that were published between 2010 and 2017. Of the three meta-analyses, Soteriades et al. consistently included the smallest number of studies in their meta-analysis, especially when they restricted their analyses to those that were judged to be of high quality. For this reason, a list of studies included in our meta-analysis more closely resembles that the Jalilian et al.’s meta-analysis, but also included additional papers published after 2017 (10, 12, 49–55). However, not all studies were included in our meta-analysis given that we eliminated data from studies with substantial geographic and chronological overlaps. In such cases we extracted data from studies that covered the greatest number of years and/or conducted in the largest geographic catchment area. Given the potential biases that might arise from the dependence in study effects, we carefully investigated study design and included study effects that are largely independent of one another. Thus, in some cases the number of independent study estimates differ from the number of studies that contributed to the study estimates.

Overall, there was some agreement for an increased risk of several cancers across the two meta-analyses as well as findings from the present analysis. For example, pooled SIRE estimates for incident prostate cancer were significantly higher in previous two meta-analyses, which ranged from 1.09 and 1.20. The range of pooled SMRE estimates for non-Hodgkin lymphoma mortality was significantly higher than general population in all three meta-analyses, which ranged from 1.20 to 1.44. Finally, the range of pooled SMRE estimates for rectal cancer mortality were significantly higher in all three analyses, which ranged from 1.16 to 1.36. Except for the pooled estimate of rectal cancer mortality found in the Soteriades et al.’s meta-analysis (SMR = 1.18 95% CI: 1.02-1.36), our estimates were lowest.

Both the present (SIR = 1.14, 95% CI: 1.08-1.21) and Jalilian et al.’s meta-analyses (95% CI: 1.21-1.45) demonstrated significantly higher risk for incident melanoma; Soteriades et al.’s finding was not statistically significant (SIR = 1.10, 95% CI: 0.77-1.58). In contrast, testicular mortality estimate was significantly higher in the present meta-analysis (SIR = 1.64, 95% CI: 1.00-2.67), but not in the Soteriades et al.’s (SIR = 1.63, 95% CI: 0.60-4.40), although the pooled estimates were similar in magnitude. Jalilian et al. did not report a summary estimate for testicular mortality. Brain and central nervous system cancer mortality rates were significantly higher in the present (SMR = 1.90, 95% CI: 1.48-2.45) and in the Soteriades et al.’s meta-analyses (SMR = 1.26, 95% CI: 1.02-1.55), while they were not in the Jalilian et al.’s (SMR = 1.25, 95% CI: 0.96-1.63), although similar in magnitude.

Risk of testicular cancer incidence was not found to be significant in the present analysis (SIR = 1.01, 95% CI: 0.83-1.21) whereas both the Jalilian et al. (SIR = 1.34, 95% CI: 1.08-1.68) and the Soteriades et al. found significantly higher risk (SIR = 1.63, 95% CI: 0.60-4.40). Reasons for such discrepancy are uncertain. No study has considered differences between seminomas and non-seminoma types of testicular cancer. However, more estimates were used in the present meta-analysis (*k* = 12), but fewer in both Jalilian et al.’s (*k* = 9) and Soteriades et al.’s (*k* = 7). Also, some of more recent studies that were included in the present meta-analysis showed lower risk testicular cancer incidence.

### Evaluating causality

Determining if elevations in cancer risk documented *via* meta-analytic reviews are causally related to the occupation of firefighting is a complex process. The IARC has developed a systematic process for determining if certain chemical, biological, and occupational exposures may be causally linked to a specific cancer *via* assessment of available cancer studies and meta-analytic synthesis, experimental animal studies, exposure studies and assessment of other relevant mechanistic data (e.g., toxicokinetic, metabolomic and genetic effects) (41, 56). In its most recent assessment, it has upgraded a previous designation of firefighter as possibly carcinogenic occupational exposure (Group 2B designation) (4) to the Group 1 designation of firefighting as carcinogenic (5). Part of the evidence in support of this elevated designation included a meta-analysis of seven studies which found an increased risk of incident mesothelioma (reported 58% increased risk; 95% CI=14%–120%) as well as a pooled increased risk of incident bladder cancer in based on the inclusion of “several” bladder cancer cohort studies (reported 16% increased risk; 95% CI=8%–26%). We did not find similar increased pooled estimates for either incident cancer (pleura [mesothelioma] 0.82; [0.62-1.08]; bladder 0.91; [0.78-1.07]) although we did find a non-significantly increased risk of pleura mortality SMRE= (1.37, 95% CI: 0.87-2.15). The forthcoming full IARC report will likely provide more details on the methods employed and the list of studies that were included in their meta-analysis for these and other cancers.

Other investigators have proposed and applied different frameworks for evaluating the causality or likelihood of cancer risks associated with firefighting that includes some of the following features: 1) consistency of reported epidemiologic findings, 2) assessment of study quality, confounders, misclassification and bias, and 3) consideration of the biologic plausibility of carcinogenic and other chemical exposures as drivers of increased cancer risk (6, 34, 39).

It is therefore important to note that completion of meta-analytic analysis of study findings is just one element of a comprehensive assessment of cancer risk that may be determined to be causally linked to exposures associated with firefighting.

### Prevention and screening opportunities in the fire service

Results of the present analysis, combined with the other recent meta-analytic reviews and the recent IARC review clearly document elevated cancer risk in firefighters, supporting the need for additional work identifying and implementing best practices to reduce the carcinogenic exposures which occur during and after fire suppression activities (57–60). Additional work on educating firefighters on strategies to reduce overall cancer risk reduction is also needed (61–63). Finally, workplace policies for more aggressive early detection of skin and prostate cancers should be considered given the noted elevated risks seen in firefighters (61, 64).

### Limitations

There are study limitations that require some caution for interpretation. First, our analysis included only a small number of studies that estimated the amount of carcinogenic exposure firefighters faced (65); but because this information is not available for virtually all studies, we have no method of correlating amount or type of exposure with cancer risk. Rather, most studies included in this meta-analysis use job title as the exposure indicator. Through a variety of sources, the general population is also exposed, to some degree, to carcinogens commonly attributed to firefighters’ activities. Healthy worker effects are likely present in studies of firefighters and may lead to a systematic underestimate of cancer risks in firefighters (66). Another limitation is the small number of cases for some cancer sites which does not allow for the calculation of stable risk estimates. This is particularly true for cancers of the bone, specific leukemias and brain cancers, and mesothelioma among others. An additional limitation is that out of 38 studies, we have only 8 studies reporting the descriptive statistics of participants by age. Seven studies reported participant mean age, between 30 to 39.4. One study reported a mean age of 57. Given that the lack of reported information and (when reported) its homogeneous nature of participants in terms of their mean age (30 – 39), we decided not to run the moderator analysis using age as a continuous predictor on SIR and SMR. Finally, the process to minimize study overlap *via* careful selection of papers with respect to geographic region and years covered was imprecise as we balanced the need for inclusion of otherwise eligible studies against avoiding the inclusion of effect sizes drawn for similar cohorts of firefighters. It was not possible to completely eliminate study overlap so some dependency effects may remain in our analysis.

Despite these limitations, we were able to minimize the inflation of type I error rates that might arise from dependency in effects by undertaking careful inspection of individual study designs for the possibility of dependency in effect sizes. In addition, we performed a variety of moderator analyses to better understand sources of the observed variations (i.e., gender, study quality scores, type of effect size). Finally, we carefully investigated all included studies and selected the most independent study effects to avoid overlap with respect to geographic region and years covered by each study.

## Conclusion

Despite differences between our study and others, our results reinforce the associations between firefighting and cancer. This occupational group faces many unique hazards and exposures, and more research employing high-quality study designs, such as the ongoing National Firefighter Registry (67) and the Career Firefighter Health Study (68), is needed to investigate how these exposures impact cancer risk. Moreover, future cohort studies that account for important confounders and employ longitudinal exposure to document the frequency, duration, and intensity, of exposures are needed. Continued improvements to personal protective equipment, adherence to safety measures in the fire service, and protective policies and legislation are imperative to keeping firefighters safe (68).

## Data availability statement

The original contributions presented in the study are included in the article/**Supplementary Material**. Further inquiries can be directed to the corresponding author.

## Author contributions

All authors listed have made a substantial, direct, and intellectual contribution to the work and approved it for publication.
