# Supplementary material for: Cancer risk and mortality among firefighters: a meta-analytic review
Source: Front Oncol. 2023 May 12;13:1130754. doi: 10.3389/fonc.2023.1130754 (PMC10213433; doi:10.3389/fonc.2023.1130754)
Supplement: Supplementary file 1 [file DataSheet_1.docx]

| **Supplementary Table 1. Characteristics of Cancer Incidence Studies included in Meta-Analytic Analysis** | | | | | | | | | | | |
| --- | --- | --- | --- | --- | --- | --- | --- | --- | --- | --- | --- |
| **Reference** | **Firefighter Catchment Area** | **Enrollment Period** | **Study Design** | **Comparison Group** | **# FF and # controls/ comparison** | **Cancer Data Source** | **Cancer coding** | **Occupational coding source** | **Additional Exposure/ stratification variables** | **Statistical Measure** | **Covariates** |
| Ahn, Jeong, and Kim (2012) | South Korea | 1980-2007 | Cohort | Korean general population | FF:29,438  Non-FF:3,978 | Korea National Central Cancer Registry (KNCCR) | ICD-10 | Employment records | All emergency responders -Experience of firefighting job (firefighting, non-firefighting) -Job duration (<10 years, >=10 years) -Classification of job duration as a surrogate variable for exposure intensity | SIR | Age  Calendar year of diagnosis |
| P. A. Demers et al. (1994) | Seattle and Tacoma, Washington | 1974-1989 | Cohort | Male graduates of the Seattle Police Academy between 1944 and 1978, policemen employed by the city of Tacoma for at least one year between 1944 and 1979, and general male population | FF: 2,447 Police: 1,878 FF cases: 224 | Cancer Surveillance System (CSS) of the Fred Hutchinson Cancer Research Center | ICD-9 | Employment records Certification records | Duration of exposure (active duty) -Age groups (5-year intervals) -Time period at risk -Firefighters vs. policemen | SIR | Age groups (five-year intervals) -Calendar periods (1974-79, 1980-84, 1985-89). |
| Firth, Cooke, and Herbison (1996) | New Zealand | 1972-1984 | Cohort | Males in full or part-time employment in New Zealand aged 15-64 years | FF: 4 | New Zealand Cancer Registry | ICD-9 | Cancer registry records | N/A | SIR | Age Socioeconomic level |
| Glass, Del Monaco, Pircher, Vander Hoorn, and Sim (2017) | Australia | 1998-2010 | Cohort | General Australian population | FF: 144,512 FF w/ cancer: 11,548 (calculated) | Australian Cancer Database (ACD) | ICD-10 | Various fire agencies | Type of volunteer firefighter -Incident types -Number of incidents -Duration of services -Era | SIR | Age -Calendar period |
| Glass, Del Monaco, Pircher, Vander Hoorn, and Sim (2016) | Fiskville, Victoria, Australia | 1971-1999 | Cohort | General Australian population, Victorian (state) population | FF:611 FF cases: 69 (calculated from Table 1) | Victorian Cancer Registry (VCR), the Australian Cancer Database (ACD), the National Death Index (NDI) | ICD-10 | Employment records and death registries | Risk of exposure group (low, medium, high) | SIR | RIR adjusted for: -Age  -Calendar period |
| Glass, Pircher, Del Monaco, Hoorn, and Sim (2016) | Australia | 1980-2011 | Cohort | Australian general population | FF:29,014FF cases: 1,693 | The cohort was linked to the National Death Index (NDI) and the Australian Cancer Database (ACD), both held by the Australian Institute of Health and Welfare (AIHW). | ICD-10 | Fire agencies | Full-time vs. part-time-Incident type-Duration of employment groups (>3 months <10 years, 10-20 years and 20+ years)-Era first employment (pre-1970, 1970-1994, post-1995) | SIR | Age-Calendar period |
| Lenahan (2018) | New Jersey (4 municipal cities) | 1981-2015 | Cohort | U.S General Population | FF w/ cancer: 758 723 (Says 758 in figure, but 723 in text on pg. 77) FF w/out cancer: 3,019 | New Jersey State Cancer Registry | ICD-O-10 | Employment records | Year of diagnosis -Age -Amount of time, in person years | SIR | Year of diagnosis -Age -Amount of time, in person years, that each subject is exposed to the environmental hazard in question |
| K. K. U. Petersen, Pedersen, Bonde, Ebbehoej, and Hansen (2018) | Denmark | 1968-2014 | Cohort | Denmark general population | FF: 9,061 FF w/ cancer: 1,389 | Danish Cancer Registry | ICD-10 | Collected personnel and membership records from employers and trade unions. Danish Supplementary Pension Fund Register (ATP) | Duration of firefighting employment (<1year vs. ≥1year vs. ≥10 years vs. ≥20 years), -Era of first employment (pre-1970 vs. 1970-1994 vs. post-1994) -Age at first employment (<25 years vs. ≥25 - <35 years vs. ≥35 years) -Employment type (full time vs. other) and function (regular vs. specialized) | SIR | Age (5-year intervals) -Calendar year (5-year intervals) |
| Pukkala et al. (2014) | Denmark, Finland, Iceland, Norway. Sweden | 1965-2005 | Cohort | Population of all five countries (Denmark, Finland, Iceland, Norway, and Sweden | FF: 16,422FF w/cancer: 2,536 | Cancer registries of Denmark, Finland, Iceland, Norway, and Sweden | ICD-10 | Census questionnaires | Country-Time period-Age group | SIR | N/A |
| Zeig-Owens et al. (2011) | New York, New York | 1996-2002 | Cohort | US population cancer rates | FF w/ cancer: 263 398? | FDNY Bureau of Health Services by questionnaire, self-report or state tumor registries | ICD-O-3 | Fire Department of the City of New York (FDNY) | WTC exposure (exposed, non-exposed) | SIR | -Age -Race  -Ethnic origin |
| Zeegers, Friesema, Goldbohm, and van den Brandt (2004) | Netherlands | 1986-1993 | Cohort | Netherland general population | N=58,279  FF with cancer: 763 | Record linkage of the full cohort to cancer registries and the Dutch national database of pa- thology reports | ICD-9 | The Dutch Occupation Classification System of the “Cen- traal Bureau voor de Statistiek. | Family histort of prostate cancer | Incidence rate ratio | -Age  -Alcohol consumption  -Meat consumption  -History of cigarrete smoking  -Fruit consumption |
| Giles, Staples, and Berry (1993) | Melbourne, Victoria, Australia | 1980-1989 | Cohort | Victorian population | FF: 2,865 FF w/ cancer: 50 | Victorian Cancer Registry | ICD-9 | Employment records | Age group (<65, >=65) -Time since start of employment (<15 years, 15-29, >=30) -Employment duration (<15 years, 15-29, >=30) | SIR | Age -Calendar year |
| Glass, Del Monaco, Pircher, Vander Hoorn, and Sim (2019) | Australia | 1998-2010 | Cohort | Australian general population | All female volunteer FF: 16,320 FF cases: 421 | Australian Cancer Database | ICD-10 | Fire agencies | Employment (paid vs. paid who attended incidents, and volunteer vs. volunteer who attended incidents) -Number of incidents attended (all incidents, all fire incidents, structural fires, landscape fires, vehicle fires) -Era of first service (Pre-1970, 1970-1994, Post-1995) | SIR | Age  -Calendar year |
| Morton (1984) | U.S. and non-U.S. | 1963-1977 | Cohort | Expected cases (comparative risk estimation) | FF cases: 4 | Cases were identified primarily by searching the records of the 24 hospitals in the four counties. | No specified – 1 specific site: Leukemia | US. Census Bureau classification and hospital records | Lymphatic and nonlymphatic leukemia | SIR | N/A |
| Ide (2014) | Central and West Scotland | 1984-2005 | Cohort | Scotland general population | Firefighters N= 2200 | Strathclyde Fire and Rescue Service (SF&RS), Information Services Division, NHS National Services Scotland | ICD-9 | Service medical records and death certificates | Age of diagnosis | N/A | N/A |
| Bigert C, Martinsen JI, Gustavsson P, Sparén P (2020). | Sweden | 1961-2009 | Cohort | Firefighters included in the Nordic Occupational Cancer (NOCCA) project. (Firefighters from all the Nordic countries). | FF: 8136 male firefighters.  Control: 16,000 Firefighters included in the Nordic Occupational Cancer (NOCCA) project. | Swedish Cancer Registry | ICD-10 | Computerized census records | Employment duration (1–9 years, 10–19 years, 20–29 years, 30 + years)  -Calendar period of follow-up (1961–1975, 1976–1990, 1991–2009) | SIR | Age -Vital status (dead-alive) |
| Marjerrison N, Jakobsen J, Grimsrud TK, et al. (2022). | Norway | 1960-2018 | Cohort | Norway male population | n = 3881 male firefighters | the Cancer Registry of Norway (CRN) | ICD-10 | The Norwegian Population Register and the Cause of Death Registry. | By year of first employment (static; <1950, 1950–1969 and ≥1970), to reflect how occupational carcinogenic exposures have changed over time; by employment duration (dynamic; <10, 10–19, 20–29, and ≥30 years), as a proxy for cumulative exposure; and by time since first employment (dynamic; <20, 20–39 and ≥40 years) to account for the latency period of cancer. | SIR | Emigrated Dead Alive  Year of birth <1950 1950–1969 ≥1970  Age at first employment (years) <30, 30–49, ≥50 |
| Webber MP, Singh A, Zeig-Owens R, et al.  (2021) | U.S | 2001-2016 | Cohort | US male population | N=915 cancer cases in 841 FDNY firefighters and n=1002 cases in 909 CFHS firefighters | Fire Department of the City of New York (FDNY) &  Career Firefighter Health Study (CFHS | ICD-10 | 15 state cancer registries and he National Cancer Institute’s Surveillance, Epidemi- ology and End Results Program | -Race /ethnicit  -5 years age groups  -calendar year (2001 to 2016) | SIR | Smoking |

| **Supplementary Table 2. Characteristics of Cancer Mortality Studies included in Meta-Analytic Analysis** | | | | | | | | | | | |
| --- | --- | --- | --- | --- | --- | --- | --- | --- | --- | --- | --- |
| **Reference** | **Firefighter Catchment Area** | **Enrollment Period** | **Study Design** | **Comparison Group** | **# FF and # controls/ comparison** | **Cancer Data Source** | **Cancer coding** | **Occupational coding source** | **Additional Exposure/ stratification variables** | **Statistical Measure** | **Covariates** |
| Ahn and Jeong (2015) | South Korea | 1992-2007 | Cohort | Korean male population | FF: 29,453  Non-FF: 3,989 | National Statistical Office (NSO) data | ICD-10 | Employment records Death certificate records | All ERs, firefighters, non-firefighters -Firefighters stratified by employment duration (<10 years, 10-20 years, >=20 years) | SMR | Intensity of exposure |
| Amadeo et al. (2015) | France | 1979-2008 | Cohort | General French male population. | FF: 10,829 | National database containing medical causes of death (INSERM-CépiDC: Institut National de la Santé et de la Recherche Médicale, centre d'épidémiologie sur les causes médicales de décès). | ICD-9 | Professional staff records | N/A | SMR | Age -Calendar year |
| Aronson, Tomlinson, and Smith (1994) | Canada | 1950-1989 | Cohort | Male Ontario population | FF: 5,414 | Canadian Mortality Data Base (CMDB) at Statistics Canada, Ontario Cancer Treatment and Research Foundation | ICD-9 | Subjects were recruited from the six fire departments within Metropolitan Toronto | Years of employment -Years since first exposure/employment -Age [<60 and >60 years] | SMR | Age -Calendar year |
| Bates et al. (2001) | New Zealand | 1977-1995 | Cohort | General New Zealand population | FF: 4,305 | New Zealand Health Information Service (NZHIS) | ICD-9 | New Zealand Health Information Service (NZHIS) | 5-year calendar period -5-year age-bands | SMR | N/A |
| Beaumont et al. (1991) | San Francisco, California | 1940-1982 | Cohort | United States death rates for comparison | FF: 3,066 | California State Registrar's Office and California Automated Mortality Linkage System | ICD-8 | Employment records Certification records Death certificate records | Time since first employment (years) -Length of employment (years) | SMR | The rate ratios were standardized for age, year, sex, and race. |
| Glass et al. (2017) | Australia | 1998-2010 | Cohort | General Australian population | FF: 144,512 FF w/ cancer: 11,548 (calculated) | Australian Cancer Database (ACD) | ICD-10 | Various fire agencies | Type of volunteer firefighter -Incident types -Number of incidents -Duration of services -Era | SMR | Age -Calendar period |
| Glass, Del Monaco, et al. (2016) | Fiskville, Victoria, Australia | 1971-1999 | Cohort | General Australian population, Victorian (state) population | FF:611 FF cases: 69 (calculated from Table 1) | Victorian Cancer Registry (VCR), the Australian Cancer Database (ACD), the National Death Index (NDI) | ICD-10 | Employment records and death registries | Risk of exposure group (low, medium, high) | SMR | RIR adjusted for: -Age  -Calendar period |
| Glass, Pircher, et al. (2016) | Australia | 1980-2011 | Cohort | Australian general population | FF:30,057 | The cohort was linked to the National Death Index (NDI) and the Australian Cancer Database (ACD), both held by the Australian Institute of Health and Welfare (AIHW). | ICD-9 | Fire agencies | Full-time vs. part-time -Incident type -Duration of employment groups (>3 months <10 years, 10-20 years and 20+ years) -Era first employment (pre-1970, 1970-1994, post-1995) | SMR | Age -Calendar period |
| Guidotti (1993) | Alberta (Edmonton & Calgary),  Canada | 1927-1987 | Cohort | Male residents of the province of Alberta | FF:3328 | Alberta Health Care Insurance Plan, death certificates, Canadian Mortality Data Base, Death certificates | ICD-9 | Employment records | Decade of entry -Duration of employment  -Exposure opportunity -City | SMR | N/A |
| Ma et al. (2005) | Florida | 1972-1999 | Cohort | Florida general population (corresponding to gender) | FF:36,813 | Office of Vital Statistics of the Florida Department of Health | ICD-9 | Certification records | Gender | SMR | Age -Calendar year |
| Musk, Monson, Peters, and Peters (1978) | Boston | 1915-1975 | Cohort | All Massachusetts males and US white males. | FF w/ cancer: 367 | Death certificates | ICD-7 | Employment records | Active vs retired other variables for all causes and accidental deaths not for cancer SMRs | SMR | N/A |
| K. U. Petersen, Pedersen, Bonde, Ebbehøj, and Hansen (2018) | Denmark | 1970-2014 | Cohort | 1: random sample of male Danish employees (other than firefighters) 2: males ever employed by Danish military | FF: 11,775 FF w/cancer: 296 | Supplementary Pension Fund Register, the Danish Civil Registration System and the Danish Register of Causes of Death | ICD-8 and ICD-10 | Danish Civil Registration System (CRS) | Type (full time, part time/volunteer)  -Duration (< 1, ≥ 1, ≥ 10 and ≥ 20 years) of firefighting employment | SMR | Age (5-year intervals) -Calendar year (5-year intervals) |
| Rosenstock, Demers, Heyer, and Barnhart (1990) | Portland, Seattle, Tacoma | 1945-1984 | Cohort | US population, and police in Seattle, Tacoma, and Portland | FF:4,392 | Department and pension board records, state motor vehicle department records, state death records, and the national death index | ICD-9 | Not reported | N/A | SMR | Age (5-year intervals) -Calendar year (5-year intervals) |
| Wende (1996) | Buffalo, New York | 1950-1982 | Cohort | U.S. white male population | FF:1,957 FF w/cancer: 158 | Multiple sources including New York State tumor registry | ICD-8 | Employment records | Age started working as firefighter (<=24, 25-29, 30+) -Year started working as firefighter (1930-1939, 1940-1954, 1955-1979)  -Latency years from onset of work to death (<20, 20-34, 35-44, 45+) -Number of years worked as firefighter (1-9, 10-19, 20-29, 30-39, 40+) -Calendar year of death (1950-1959, 1960-1969, 1970-1979, 1980-1988) -Age at death (30-44, 45-59, 60-74, 75+) | SMR | Age |
| Berg (1975) | U.S. and U.K. | 1949-1963 | Cohort | Other occupations | FF cases: 39 | Death certificates from US and Great Britain | Not specified – 1 specific cancer, Bowel | Death certificate records | N/A | SMR | N/A |
| Eliopulos, Armstrong, Spickett, and Heyworth (1984) | Western Australia | 1939-1978 | Cohort | Western Australian males | FF: 990 FF w/cancer:30 | Death certificates | ICD-8 | Death certificates | Era of first employment -Time since first employment -Duration of first employment | SMR | N/A |
| Mastromatteo (1959) | Ontario, Canada | 1921-1953 | Cohort | 1: general Ontario male population   2: city male population | FF:1500 FF w/cancer: 34 | Death certificates | Not specified | Superannuation and Benefit Fund, City Fire Department. | N/A | SMR | N/A |
| Glass et al. (2019) | Australia | 1998-2011 | Cohort | Australian general population | All female volunteer FF:37,973 FF w/cancer:268 | National Death Index | ICD-10 | Fire agencies | Employment (paid vs. paid who attended incidents, and volunteer vs. volunteer who attended incidents) -Number of incidents attended (all incidents, all fire incidents, structural fires, landscape fires, vehicle fires) -Era of first service (pre-1970, 1970-1994, Post-1995) | SMR | Age -Calendar year |
| Blair, Walrath, and Rogot (1985) | U.S. | 1954-1970 | Cohort | Other occupations | FF w/ cancer:8 | United States have recently been reviewed by the Occupational Cancer Risk Subcommittee | ICD-7 | Questionnaire | Smoking | SMR | Year of follow-up  -Each given age |
| Dolin (1992) | U.K. | 1965- 1980 | Cohort | England and Wales census | FF cases: 3 | Death certificates from the Office of Population Censuses and Surveys (OPCS) | Not specified – 1 site (bladder cancer) | 1971 census | N/A | SMR | Age  -Degree of urbanization |
| Pion (1995) | U.S. | 1982-1988 | Cohort | Controls matched for age, sex, race, and geographic location on an approximately 1:3 basis to persons selected from the remaining people enrolled in the database with no malignant melanoma. | FF: 16 FF cases: 7 | American Cancer Society's Cancer Prevention II Study | Not specified – 1 site: Malignant Melanoma cancer | American Cancer Society's Cancer Prevention II Study | N/A | SMR | Age -Sex -Race -Geographic location |
| Zhao G, Erazo B, Ronda E, Brocal F, Regidor E (2020). | Spain | 2001-2011 | Cohort | Spain general population | FF: 27, 365. Non-FF:  N=9,579,759 | The Spanish National Statistics Institute Census 2001. | ICD-10 | Population registry and mortality registry | All other occupations. | aSMR | Age (20-24, 25-29, 30-34 in 4-years increments until 60-64). |
| Pinkerton L, Bertke SJ, Yiin J, Dahm M, Kubale T, Hales T, Purdue M, Beaumont JJ, Daniels R (2020). | United States – San Francisco, Chicago, Philadelphia | 1950-2016 | Cohort | US general population | FF: N=29,992 | The National Death Index-Plus, the Social Security Administration Death Master File and the Internal Revenue Service. | ICD-10 | Employment record from each firefighter department of San Francisco (SFFD), Chicago (CFD) or Philadelphia (PFD). | Three separate exposure surrogates (exposed-days, fire runs and fire-hours) linked to job exposure matrices based on job, location, and fire-fighting apparatus assignments  -Employment duration (<10, 10 to <20, 20 to <30, 30+ years).  -Time since exposure (lag to <20, 20 to <30, 30+ years), age at exposure (<40, 40+ years) and exposure period (<1970, 1970+) | SMR | Race -Vital status (dead-alive) -Gender |
| McDowell (1986) | U.K. | 1971-1980 | Cohort | England and Wales male population | 2,434  cases, F= 6 cases | N/A | ICD-9 | Death certificates | -Occupation | N/A | N/A |
| Ide (2014) | Central and West Scotland | 1984-2005 | Cohort | Scotland general population | Firefighters N= 2200 | Strathclyde Fire and Rescue Service (SF&RS), Information Services Division, NHS National Services Scotland | ICD-9 | Service medical records and death certificates | Age of diagnosis | N/A | N/A |

| **Supplementary Table 3. Cancer Incidence Findings Summary** | | | | | |
| --- | --- | --- | --- | --- | --- |
| **Reference** | **Firefighter Catchment Area / Enrollment Period** | **Included all cancers combined and cancer subtypes** | **Overall risk** | **Significantly increased risk** | **Significantly decreased risk** |
| Ahn, Jeong, and Kim (2012) | South Korea  1980-2007 | All, parotid gland, esophagus, stomach, small intestine, colon & rectum, liver & intrahepatic bile ducts, gallbladder & extrahepatic ducts, pancreas, larynx, bronchus & lung, bone & articular cartilage, prostate, kidney, bladder, brain, thyroid, non-hodgkins lymhoma, leukemia, lymphohematopoieticca | SIR: 0.97  95% CI (0.88-1.06) | Colorectal Kidney Bladder Non-Hodgkin's lymphoma | * |
| Bates (2007) | California  1988-2003 | Subtypes: esophagus, stomach, cecum, colo-rectal, pancreas, lung & bronchus, melanoma-skin, prostate, testis, bladder, kidney & renal pelvis, brain, thyroid, non-hodgkin's lymphoma, multiple myeloma, leukemias | OR** | Testicular Melanoma Brain Esophageal Prostate | *For no control exclusions:*  Colorectal Bladder |
| Bates et al. (2001) | New Zealand  1977-1995 | All and subtypes (esophagus, stomach, colon, rectum, pancreas, lung, melanoma, prostate, testis, bladder, kidney, brain, myeloleukemia) | *Period 1977-1996*  SIR: 0.95, 95% CI (0.8-1.1) *Period 1990-96* SIR 1.01, 96% CI (0.8-1.3) | *Period 1977-1996:* kidney  *Period 1990-96:*  Stomach Testicular | * |
| Bigert et al. (2016) | Non-US: Canada, China, Czech Republic, France, Germany, Hungary, Italy, New Zealand, Poland, Romania, Russia, Slovakia, Spain, Sweden, United Kingdom  1985-2010 | Lung cancer cell type - adenocarcinoma, squamous cell carcinoma, small cell carcinoma, other/unspecified | OR: 1.03 95% CI (0.77 – 1.38) | * | * |
| R. D. Daniels et al. (2015) | San Francisco, California - Chicago, Illinois - Pennsylvania, Philadelphia 1950-2009 | All and subtypes bladder, colorectal, oesophageal, lung, and prostate cancers; leukaemia; and non-Hodgkin's lymphoma (NHL) | HR 0.96  95% CI (0.87-1.05) | *For CDF* group:*  Lung | * |
| Robert D Daniels et al. (2014) | San Francisco, California - Chicago, Illinois - Pennsylvania, Philadelphia 1950-2009 | All cancers and subtypes brain, stomach, oesophagus, intestines, rectum, kidney, bladder, prostate, testes, leukaemia, multiple myeloma and NHL | SIR: 1.09 95% CI (1.06 to 1.12) | Oesophagus, intestine, large intestine, lung, kidney, bladder, mesothelioma, buccal, pharynx | Malignancy other male genital  Multiple myeloma |
| Delahunt, Bethwaite, and Nacey (1995) | New Zealand  1978-1986 | Renal cell carcinoma | RR: 3.51 95% CI (2.09-5.92) | Renal cell carcinoma | * |
| P. A. Demers et al. (1994) | Seattle and Tacoma, Washington  1974-1989 | Subtypes by histoly: oral and pharynx, esophagus, stomach, colon, rectum, pancreas, sinus, larynx, lung, trachea, and bronchus, melanoma of skin, breast, prostate, bladder, kidney, occular melanoma, brain, thyroid, Hodgkin's disease, non-Hodgkins lymphoma, multiple myeloma, leukemia | SIR: 1.1 95% CI (0.9-1.2) | Prostate | * |
| Elci, Akpinar-Elci, Alavanja, and Dosemeci (2003) | Turkey, Istanbul  1979-1984 | Subtypes and subtypes by histology: lung cancer, squamous cells, smallcell, large cells, adenocarci-nomas, and other types of lung cancers | OR: 6.8  95% CI (1.3-37.4) | Lung | * |
| Firth, Cooke, and Herbison (1996) | New Zealand  1972-1984 | Subtype*s:* buccal cavity, oesophagus, stomach, colon, rectum, liver, pancreas, larynx, lung, melanoma, prostate, testis, bladder, other urinary, eye, brain, lymphosarcoma, Hodgkin's disease, leukaemia | SIR: 1348 95% CI (254-3991) | Laryngeal | * |
| Gaertner, Trpeski, and Johnson (2004) | Newfoundland, Prince Edward Island, Nova Scotia, Manitoba, Alberta, Saskatchewan, and British Columbia  1994-1997 | Bladder cancer | OR: 1.51 95% CI (0.59-3.84) | * | * |
| Glass, Del Monaco, Pircher, Vander Hoorn, and Sim (2017) | Australia  1998-2010 | All and subtypes lip, oral cavity, and pharynx, lip, digestive organs, oesophagus, stomach, colorectal, colon, rectum, liver, pancreas, respiratory, larynx, lung, melanoma, mesothelioma, male, reproductive, prostate, testis, urinary tract, kidney, bladder, brain and other CNS, brain, thyroid and other endocrine, thyroid, unknown site, lymphohaematopoetic, Hodgkin's disease, NHL, follicular NHL, diffuse NHL, myeloma, leukaemia, MDS, all other (bone and connective tissue, eye, rare lymphohaematopoetic conditions and cancer of multiple sites) male breasts) | *Volunteer Male FF:*  SIR 0.86  95% CI (0.84-0.88) | *Paid FF:*  Prostate Melanoma *Volunteer FF:*  Prostate | Digestive organs, stomach, colon, liver, respiratory, lung, mesothelioma, urinary tract, bladder, unkown sites, lymphohaematopoetic, Non-Hodgkin's Lymphoma, and diffuse Non-Hodgkin's Lymphoma |
| Glass, Del Monaco, Pircher, Vander Hoorn, and Sim (2016) | Fiskville, Victoria, Australia 1971-1999 | Lip, buccal cavity and pharynx, digestive, respiratory system, melanoma, male reproductive, prostate, testis, urinary tract, brain and nervous system, lymphohaematopoietic, other and unknown, overall cancer | SIR: 1.85 95% CI (1.20-2.73) | Testicular Melanoma  Brain and nervous system | * |
| Glass, Pircher, Del Monaco, Hoorn, and Sim (2016) | Australia 1980-2011 | All cancers and subtypes (Lip, oral cavity and pharynx, digestive organs, colorectal, respiratory, lung, melanoma, male reproductive, prostate, testis, urinary tract, kidney, Lympho-haematopoietic, non-Hodgkin lymphoma) | All FF SIR 1.09  95% CI (1.03 to 1.14) | Melanoma  Prostate  Male reproductive | Live Respiratory Lung |
| Harris et al. (2018) | Alberta, British Columbia, Manitoba, New Brunswick, Newfoundland, Nova Scotia, Ontario, Prince Edward Island, Quebec, Saskatchewan, Territories  1992-2010 | All cancer and subtypes (lip, oral, esophagus, stomach, colon, rectum, liver, pancreas, bladder, kidney, brain, thyroid, nasal, larynx, lung, mesothelioma, bone, melanoma, breast, Hodgkin, myeloma, NHL, leukemia, prostate, early prostate, testis) | HR: 1.06  95% CI (0.97-1.16) | Melanoma  Hodgkin's lymphoma Prostate | * |
| Ide (2014) | Central and West Scotland  1984-2005 | All cancers, melanoma, lymphoma, testicle, kidney, large bowel , lung, basal cell carcinoma, bladder, brain | IR (SD)   86.5 (64.2) | Melanoma Kidney | Lung Lymphoma |
| Kang, Davis, Hunt, and Kriebel (2008) | Massachusetts  1987-2003 | Lip, Buccal cavity, Nasopharynx , Esophagus, Stomach, Colon, Rectum, Liver, Pancreas, Larynx , Lung , Skin melanoma, Soft tissue sarcoma, Breast, Prostate, Testicular, Kidney, Bladder, Brain, Thyroid, Leukemia, Non-Hodgkins Lymphoma, Hodgkins, Lymphoma Multiple myeloma | SMORS*** | Colon Brain | Skin melanoma Esophagus |
| Krishnan et al. (2003) | San Francisco, California  1991-1994 1997-1999 | Subtypes and subtypes by histology: gliomas Histologic distribution: glioblastoma, anaplastic astrocytoma, astrocytoma, oligoastrocytoma (mixed), oligodendroglioma, ependymoma, other | *Longest held occupation* OR: 5.88, 95% CI (0.70-49.01) *Ever employed* OR: 2.85, 95% CI (0.77-10.58) | * | * |
| Kullberg et al. (2018) | Stockholm, Sweden  1958-2012 | All and subtypes Malignant tumor, Lip, Esophagus, Stomach, Colon, Rectum and anus, Biliary passages of liver, Pancreas, Bronchus and lung primary, Pleura, Prostate, Kidney, Urinary organs, Malignant melanoma of skin, Non-melanoma skin cancer, Brain nervous system, Endocrine glands, Connective tissue muscle, All haematopoietic cancer, Malignant non-Hodgkin lymphoma, Hodgkin's disease, Multiple myeloma, plasmocytoma, Leukemia | SIR: 0.81  95% CI (0.71-0.91) | Stomach | Prostate Malignant melanoma of the skin |
| Lenahan (2018) | New Jersey (4 municipal cities)  1981-2015 | All, buccal and pharynx, lip, tongue, other buccal, pharynx, colorectal, large intestine, rectum, digestive & peritoneum excluding colorectal, esophagus, stomach, small intestine, gallbladder, pancreas, peritoneum & other unspecified, respiratory & intrathoracic organs, larynx, trachea, bronchus, lung, other respiratory, breast (male), male genital organs, prostate, testes, other male genital, urinary, kidney, bladder & other urinary, endocrine, thyroid, other solid tumors ? - don't code at allmelanoma (skin) melanoma in situ ? - don't code at allmesothelioma/pleura, connective, brain & other nervous, eye, lymphatic & hematopoietic, hodgkin's disease, non-hodgkin's disease multiple myeloma, leukemia other lympho-hematopoietic | SIR: 0.72  95% CI (0.67-0.77). | Eye | * |
| Ma, Fleming, Lee, Trapido, Gerace (2006) | Florida  1981-1999 | Buccal, Digestive, Esophagus, Stomach, Colon, Rectum, Liver , Pancreas, Respiratory, Larynx, Lung/bronchus, Bone, Skin,† Bladder, Kidney, Eye, Brain/central nervous system, Thyroid, All lymphopoietic‡, Non-Hodgkin‡, Hodgkin‡, Leukemia‡ Prostate, Testes, Breast, Soft tissue, sarcoma | *Females FF* SIR 1.63, 95% CI (1.22-2.14)  *Males FF*  SIR 0.84, 95% CI (0.79 – 0.90) | *Males FF*:  Badder, Testicular, Thyroid  *Female FF:*  Cervical, Non Hodgkin's lymphoma, Thyroid | Males FF: buccal, digestive, stomach, lung/bronchus, brain/central nervous system, and all lymphopoietic |
| Moir et al. (2016) | San Francisco, Chicago, New York, Philadelphia 1996-2009 | Colon, thyrod, melanoma, hematologic, prostate, lung | RR: 0.96  95%CI ( 0.83–1.12) | Thyroid  Prostate | Lung |
| Paget-Bailly et al. (2013) | Departments of France (Bas-Rhin, Calvados, Doubs, Haut-Rhin, Herault, Isere, Loire-Atlantique, Manche, Somme, Vendee)  2001-2007 | Subtypes and subtypes by histology: lip, OC, and pharynx, nasal cavity and accessory sinuses, and larynx | OR:3.9 95% CI (1.4 -11.2) | Head  Neck | * |
| K. K. U. Petersen, Pedersen, Bonde, Ebbehoej, and Hansen (2018) | Denmark  1968-2014 | All (minus other skin), lip, tongue, mouth, salivary glands pharynx, esophagus, stomacH, colon, rectum, liver, gallbladder, pancreas, anus, nasal cavity, larynx, lung, heart and mediastinum, bones, melanoma, other skin, mesothelioma/pleura, connective tissue, prostate testis, other male genital, kidney, renal pelvis, bladder, eye, meninges, brain, other central nervous system, thyroid, hodgkin's lymphoma, non-hodgkin's lymphoma, myeloma, lymphatic leukaemia, myeloid leukaemia, ill-defined/unspecified other | SIR 1.02  95% CI (0.96 to 1.09) | Melanoma of the skin Other skin Prostate Heart andmediastinum | Colon |
| Pukkala et al. (2014) | Denmark, Finland, Iceland, Norway. Sweden  1965-2005 | Lip, Tongue, Salivary glands, Oral cavity, Pharynx, Oesophagus, Stomach, Small intestine, Colon, Rectum, rectosigma, Primary liver, Gallbladder, Pancreas, Larynx, Lung, Adenocarcinoma, Squamous cell carcinoma, Small cell carcinoma Skin melanoma, Mesothelioma, Soft tissue, Penis, Prostate, Testicular, Kidney, Bladder, Brain, Glioma Thyroid, Non-Hodgkin, lymphoma, Multiple myeloma, Leukaemia, Acute myeloid, Non-melanoma skin cancer | SIR: 1.06  95% CI (1.02-1.11) | Prostate Skin melanoma Non-melanoma skin Adenocarcinoma of the lung | Testicular  Lung |
| Sama, Martin, Davis, and Kriebel (1990) | Massachusetts  1982-1986 | Colon, rectum, pancreas, lung, bronchus, and trachea, melanoma of skin, bladder, brain and other nervous system, non-Hodgkin's lymphoma, leukemia | SMORS** | Melanoma Lymphiama Bladder | * |
| J. Sritharan et al. (2017) | Canadian provinces: Newfoundland, Alberta, British Columbia, Manitoba, Saskatchewan, Nova Scotia, Prince Edward Island, Ontario  1994-1997 | Prostate | mRE: 1.17  95% CI (1.08–1.28) | Prostate | * |
| Stang, Jockel, Baumgardt-Elms, and Ahrens (2003) | Five German geographic regions (cities of Bremen, Essen, Hamburg, Saarbru¨cken, and the Federal State of Saarlan 1995-1997 | Testicular | *Ever mployed*  OR1 4.0 95% CI (0.7-27.4) OR2 4.3 95% CI (0.7-30.5) | * | * |
| Jeavana Sritharan et al. (2018) | Ontario, Quebec, Manitoba, Saskatchewan, Alberta, British Columbia, Yukon, NWT, Nunavut, Newfoundland, Prince Edward Island, Nova Scotia, New Brunswick 1991-2010 | Prostate | HR: 1.17 95% CI: (1.01–1.36) | Prostate | * |
| Tornling, Gustavsson, and Hogstedt (1994) | Stockholm, Sweden  1958-1986 | All, stomach, colon, rectum and anus, liver, pancreas, bronchus and lung, melanoma, other skin, prostate, kidney, brain, all hematomietic | SIR: 100  95% CI (83-119) | Stomach  Brain | All hematomietic |
| Tsai et al. (2015) | California  1988-2007 | Lip, tongue, salivary gland, gum and other mouth, pharynx, esophagus, stomach, colorectal, liver, pancreas, larynx, lung and bronchus, soft tissue including heart, melanoma, mesothelioma/pleura, prostate, testis, bladder, kidney, brain, thyroid, hodgkin's lymphoma, non-hodgkin's lymphoma, multiple myeloma, leukemia, chronic lymphocytic leukemia, acute myeloid/monocytic leukemia, chronic myeloid leukemia | OR** | Melanoma Multiple myeloma Acute myeloid leukemia Esophagus Prostate Brain Kidney  Urinary blander Tongue Non-specific, non-small cell  Leukemia | Larynx |
| Zeegers, Friesema, Goldbohm, and van den Brandt (2004) | Netherlands  1986-1993 | Prostate and subtypes skin | RR 0.57 95% CI (0.05 5.92) | * | * |
| Zeig-Owens et al. (2011) | New York, New York  1996-2002 | All cancers, esophagus, stomach, colon, pancreas, lung, melanoma, prostate, testicular, bladder, kidney, thyroid, hodgkin's lymphoma, non-hodgkin's lymphoma, multiple myeloma, leukemia, and subtypes prostate, thyroid, non-Hodgkin lymphoma | SIR: 1.02  95% CI 1·21 (0·98–1·49) | Thyroid | * |
| Bates and Lane (1995) | Wellington, New Zealand  1980-1991 | Testicular | SIR: 16.7 95% CI (1.9-60) | Testicular | * |
| Giles, Staples, and Berry (1993) | Melbourne, Victoria, Australia  1980-1989 | Upper aerodigestive, colorectal, pancreas, lung, melanoma, prostate, testis, urinary tract, non-hodgkin's lymphoma, other haematopoietic, leukemia | SIR: 1.13  95% CI (0.84-1.48) | Colorectal | * |
| P. Demers, Martinsen, J. I., Weiderpass, E., Kjaerheim, K., Lynge, E., Sparen, P., & Pukkala, E. (2011) | Denmark, Finland, Norway, Sweden 1960-1990 | Other skin, melanoma, colon, prostate, mesothelioma/pleura | SIR: 1.03 95% CI (0.97-1.09) | Non-melanoma skin cancer, lung adenocarcinoma, malignant melanoma, colon cancer, prostate cancer, and mesothelioma | * |
| Fang, Le, and Band (2011) | British Columbia, Canada  1983-1990 | Colon cancer | *Ever FF* OR: 0.95, 95% CI (0.40-2.25) *Usual FF* OR: 1.14, 95% CI (0.50-2.60) | Colon | * |
| Glass, Del Monaco, Pircher, Vander Hoorn, and Sim (2019) | Australia  1998-2010 | All lip, oral cavity and pharynx, digestive organs, colorectal, colon, rectum, respiratory, lung, melanoma, mesothelioma/pleura, breast, female reproductive, cervix, urinary tract, kidney, brain and other cns, brain, thyroid and other endocrine, thyroid, unknown site, lymphohematopoetic, non-hodgkin's lymphoma, myeloma, leukemia, all other cancers | *Volunteer Female FF*  SIR: 0.97  95% CI (.91-1.03) | Melanoma | Cervix |
| Karami et al. (2012) | Illinois, Chicago - Detroit, Michigan 2002-2007 | Kidney and subtype renal cell carcinoma | *Ever FF* OR: 1.4, 95% CI (0.4-4.7) *<5 years FF* OR: 3.2, 95% CI (0.0-8.8E + 09) *5+ years FF* OR: 1.1, 95% CI (0.3-4.8) | * | * |
| Morton (1984) | U.S. and non-U.S.  1963-1977 | Leukemia | SIR 346 P < .01 | Nonlymphatic leukemia | * |
| Corbin et al. (2011) | New Zealand  2007-2008 | Lung | *Not semi-Bayes adjusted*  OR: 0.76 95% CI (0.17-3.45) | * | * |
| Krstev BSD (1998) | Georgia, Michigan, New Jersey  1986-1989 | Prostate | OR: 3.85  95%CI (1.34–11.10) | *White FF:* Prostate cancer | * |
| Lee et al (2020) | Florida  1981-2014 | All, oral cavity and pharynx, esophagus, larynx, stomach, colon, rectum, pancreas, liver, bones and joints, soft tissue including heart, lung, melanoma, breast, cervix uteri, prostate, testes, penis, bladder 188 kidney and renal pelvis, eye and orbit, brain, thyroid, hodgkin's lymphoma, non-hodgkin's lymphoma, myeloid, multiple myeloma, acute monocytic leukemia, acute myeloid leukemia, acute lymphocytic leukemia, chronic lymphocytic leukemia, chronic myeloid leukemia, mesothelioma/pleura, other | OR** | *Male FF:*  Melanoma, prostate, testicular, thyroid and late-stage colon  *Female FF:*  Brain  Thyroid | *Male FF:*  Larynx, Liver, Lung, Oral cavity and pharynx, Acute Myeloid Leukemia,  Myeloid *Female FF:*  Brain  Thyroid |
| Krstev (1998) | Colorado, Georgia, Idaho, Indi-ana, Kansas, Kentucky, Maine, Missouri, Nebraska, Nevada,New Hampshire, New Jersey, New Mexico, North Carolina, Ohio, Oklahoma, Rhode Island, South Carolina, Tennessee,Utah, Washington, West Virginia, Wisconsin, Vermont 1984-1993 | Prostate | MOR: 1.2 95% CI 1.0–1.4 | Prostate | * |
| Alguacil (2003) | Sweden  1989 | Pancreas | *Male FF* SIR 135.7 95% CI (0.59-2.08) *Female FF* SIR 136.2 95% CI (0.55 to 2.26) | * | * |
| Goodman (1995) | California  1972-1990 | Melanoma | OR: 2.1  95% CI (1.4-3.2) | Cutaneous melanoma | * |
| GreeneKS (2008) | San Francisco  2006-2007 | Bladder | OR** | * | * |
| Carozza (2000) | San Francisco  1991-1994 | Brain and subtype gliomas | OR: 2.7 95% CI (0.3-26.1) | * | * |
| De Roos (2003) | Arizona, Massachusetts, Philadelphia  1994-1998 | Brain and subtype gliomas | OR: 0.3 95% CI (0.1, 1.7) | * | * |
| Sritharan (2019) | Ontario, Canada  1983-2015 | Prostate | HR:1.62  95% CI (1.47–1.78) | Prostate | * |
| Huebner (1992) | California, Georgia, New Jersey  1984-1985 | Subtypes oral and pharyngeal | OR: 0.65 95% CI (0.23-1.85) | * | * |
| Guenel (1990) | Denmark  1970-1980 | Larynx | RR: 5.45** | * | * |
| Burns (1991) | Detroit Metropolitan Area  1980 | Lung and bronchus | OR: 1.33 95% CI (0.53-3.35) | * | * |
| Pion (1995) | U.S. 1982-1988 | Subtypes malignant melanoma | OR: 2.29 95% CI (0.85-6.16) | * | * |
| Band (2004) | British Columbia  1983-1990 | Subtypes non-hodgkin's lymphoma and follicular | OR: 3.64 95% CI (1.22-10.81) | Follicular | * |
| Bigert C, Martinsen JI, Gustavsson P, Sparén P (2020) | Sweden  1961-2009 | Pharynx, esophagus, stomach, colon, rectum, liver, pancreas, larynx, lung, mesothelioma, prostate, testes, kidney, bladder, melanoma skin, non-melanoma, skin, non-hodgkin lymphoma, multiple myeloma, leukemia, chronic lymphatic leukemia | SIR 1.03 95% CI (0.97-1.09) | Non-melanoma skin | * |
| Webber MP, Singh A, Zeig-Owens R, et al (2021) | New York, United States  2001-2016 | All cancers, prostate, lung, kidney, non-hodgkins lymphoma, melanoma, thyroid | *FDNY*** FF:* SIR: 1.15, 95% CI (1.08 -1.23) *CFHS*** FF*  SIR: 1.05, 95% CI (0.98 to 1.12) | *FDNY*** FF:*  Prostate, melanoma, Non Hodgkin's lymphom, and thyroid  *CFHS*** FF:*  Prostate  Melanoma | *Both FDNY and CFHS FF:*  Lung |
| Marjerrison N, Jakobsen J, Grimsrud TK, et al. (2022) | Norway 1960-2018 | All cancers, larynx, lung, melanoma, non-melanoma skin, mesothelioma, kidney, urinary tract, hodgkins, lymphoma, non-hodgkins, lymphoma, multiple, myeloma, leukemia | SIR: 1.15  95% CI (1.07-1.23) | Urinary tract Mesothelioma Laryngeal | * |

*Statistically increase/decrease not found.

** Overall risk not calculated.

***Fire departments in Chicago (CFD), Philadelphia (PFD) and San Francisco (SFFD); Fire Department of the City of New York (FDNY), Career Firefighter Health Study (CFHS).

| Supplementary Table 4. Cancer Mortality Findings Summary | | | | | |
| --- | --- | --- | --- | --- | --- |
| Reference | Firefighter Catchment Area / Enrollment Period | Included Cancers (All or Specific Cancer) | Overall risk | Significally increased risk | Significantly decreased risk |
| Ahn and Jeong (2015) | South Korea  1992-2007 | All, stomach, colorectal, liver & intrahepatic bile duct, bronchus & lung, leukemia, lymphohematopoietic | SMR: 0.58  95%CI ( 0.50–0.68) | Leukemia | Stomach |
| Amadeo et al. (2015) | France  1979-2008 | All and subtypes rectum and anus, pancreas, stomach, lip, oral cavity and pharynx, larynx and trachea, liver, esophagus, lymph/haematopoietic tissue, bronchus and lung, breast, bladder, colon, skin, kidney, prostate | SMR: 0.95 95% CI (0.88-1.02) | * | Bronchus Lung Prostate |
| Aronson, Tomlinson, and Smith (1994) | Canada  1950-1989 | Subtypes by histology Pharynx, Esophagus, Stomach, Colon, Rectum and rectosigmoid junction, Liver and bile ducts, Pancreas, Larynx, Trachea, bronchus, and lung, Malignant melanoma, Prostate, Testis, Bladder, Kidney and ureter, Brain and other nervous system, Lymphatic and hematopoietic tissue, Lymphosarcoma, Hodgkin's disease, Multiple myeloma, Lymphatic leukemia, Myeloid leukemia | SMR: 105  95% CI (91-120) | Brain Other nervous system Other malignant neoplasms | * |
| Baris et al. (2001) | Philadelphia, Pennsylvania  1925-1986 | All and subtypes Buccal cavity and pharynx, Esophagus, Stomach, Colon, Rectum, Liver, Pancreas, Larynx, Lung, Skin, Prostate, Bladder, Kidney, Brain, Non-Hodgkin's lymphoma, Multiple myeloma, Leukemia, Benign neoplasms | SMR: 1.10  95% CI (1.00-1.20) | Colon cancer | * |
| Bates et al. (2001) | New Zealand  1977-1995 | All, subtypes Esophagus, Stomach, Colon, Rectum, Pancreas, Lung, Melanoma, Prostate, Testis, Bladder, Kidney, Brain, Myeloleukemia | SMR: 0.81  95% CI (0.6-1.1) | * | * |
| Beaumont et al. (1991) | San Francisco, California  1940-1982 | All and subtypes Buccal cavity and pharynx, Lip, Tongue, Pharynx, Digestive organs and peritoneum, Esophagus, Stomach, Intestine except rectum, Rectum, Biliary passages, liver, gall bladder, Pancreas, Respiratory system, Larynx, Trachea, bronchus, and lung, Genital organs, Prostate, Urinary organs, Kidney, Bladder and other urinary, Other and unspecified malignant neoplasms, Skin, Brain and other nervous system, Lymphatic and hematopoietic tissue, Lymphosarcoma and reticulosarcoma, Leukemia and aleukemia | SMR: 0.95  95% CI (0.84 1.08) | Digestive organs Peritoneum Esophageal | Genital orgarms Prostate |
| R. D. Daniels et al. (2015) | San Francisco, California Illinois, Chicago Pennsylvania, Philadelphia 1950-2009 | All cancer, colorectal, esophagus, lung, prostate, non-hodgkins lymphoma, leukemia, and subtypes bladder, colorectal, oesophageal, lung, and prostate cancers; leukaemia; and non-Hodgkin's lymphoma (NHL). | HR: 0.95 95% CI (0.90- 0.99) | *For CFD*** and PFD***:* Lung Leukemia | *For CFD*** and PFD***:*  Colorectal Prostate |
| Robert D Daniels et al. (2014) | San Francisco, California Illinois, Chicago Pennsylvania, Philadelphia 1950-2009 | All cancers, buccal and pharynx, esophagus, stomach, intestines, large intestine, small intestine, rectum, lung, breast, prostate, other male genital, testes, kidney, bladder, brain, non-hodgkin's lymphoma, leukemia, multiple myeloma, other cancers, mesothelioma/pleura | SMR: 1.14 95% CI (1.10 to 1.18) | Oesophagus Intestine/Large intestine Rectum Lung Kidney Mesothelioma Buccal Pharynx cancer | * |
| Deschamps, Momas, and Festy (1995) | Paris, France  1977-1991 | Pharynx, digestive, respiratory, genito-urinary, other | SMR: 0.89  95% CI (0.53-1.40) | * | * |
| Feuer and Rosenman (1986) | New Jersey  1974-1980 | All cancer, digestive, respiratory, skin, leukemia | PMR: 1.00 P < 0.05. | Leukemia  Skin | * |
| Figgs, Dosemeci, and Blair (1995) | Colorado, Georgia, Idaho, Indiana, Kansas, Kentucky, Maine, Missouri, Nebraska, Nevada, New Hampshire, New Jersey, New Mexico, North Carolina, Ohio, Oklahoma, Rhode Island, South Carolina, Tennessee, Utah, Washington, West Virginia, Wisconsin, and Vermont 1984-1989 | Subtypes Non-Hodgkin's Lymphoma | OR: 5.6 95% CI (2.5-12.3) | Non-Hodgkin’s Lymphoma | * |
| Glass et al. (2017) | Australia  1998-2010 | All and subtypes lip, oral cavity, and pharynx, lip, digestive organs, oesophagus, stomach, colorectal, colon, rectum, liver, pancreas, respiratory, larynx, lung, melanoma, mesothelioma , male reproductive, prostate, testis, urinary tract, kidney, bladder, brain and other CNS, brain, thyroid and other endocrine, thyroid, unknown site, lymphohaematopoetic, Hodgkin's disease, NHL, follicular NHL, diffuse NHL, myeloma, eukaemia, MDS, all other (bone and connective tissue, eye, rare lymphohaematopoetic conditions and cancer of multiple sites) male breasts | *All Volunteer FF*: SMR 0.59  95% CI (0.57-0.62) *Volunteers FF in incidents:  SMR: 0.59  95% CI (0.55 to 0.62)* | * | * |
| Glass, Del Monaco, et al. (2016) | Fiskville, Victoria, Australia 1971-1999 | All cancer, lip, buccal cavity and pharynx, digestive, respiratory system, melanoma, male reproductive, prostate, testis, urinary tract, brain and nervous system, lymphohaematopoietic, other and unknown | *Low risk group:* SMR: 0.29  95% CI (0.01–1.64) Medium risk group: *Paid FF:* SMR 0.89  95% CI(0.24–2.27) *Volunteer FF:* SMR 0.85 95% CI (0.28–1.99) High risk group: SMR: 1.47,  95% CI (0.54–3.19) | Testicular  Melanoma  Brain and nervous system | * |
| Glass, Pircher, et al. (2016) | Australia 1980-2011 | Lip, oral cavity and pharynx, digestive organs, colorectal, respiratory, lung, melanoma, male reproductive, prostate, testis, urinary tract, kidney, Lympho-haematopoietic, non-Hodgkin lymphoma | SMR: 0.81  95% CI (0.74-0.89) | * | All cancer mortality reduced |
| Grimes, Hirsch, and Borgeson (1991) | Honolulu, Hawaii 1969-1988 | Digestive, stomach, colon, respiratory, genito-urinary, prostate, brain and other CNS, lymphatic system | RR: 1.9 95% CI (0.96-1.49) | Brain and other NSC Lympathic system Prostate Genito-urinary system | * |
| Guidotti (1993) | Alberta (Edmonton & Calgary),  Canada  1927-1987 | Oral, stomach, colon and rectum, pancreas, lung , skin, prostate, bladder, kidney and ureter, brain, leukemia, lymphoma, myeloma | SMR 126.6 95% CI (102.0-155.2) | Kidney Ureter | * |
| Hansen (1990) | Denmark 1970-1980 | All cancers, lung cancer, other | SMR: 117  95% CI (72-178) | Lung Non-pulmonary | * |
| Ide (2014) | Central and West Scotland 1984-2005 | All cancers, melanoma, lymphoma, testicle, kidney, large bowel, lung, basal cell carcinoma, bladder, brain | MR (SD) 20.4 (27.4) | Kidney Renal  Testical Bladder  Brain Melanoma Lung | * |
| Ma et al. (2005) | Florida 1972-1999 | Buccal/ pharynx, digestive, esophagus, stomach, colon, rectum, liver, pancreas, respiratory, larynx, lung and bronchus, bone, skin, bladder, brain/ CNS, thyroid, all lymphopoietic, lymphosarcoma, hodgkin's, leukemia, prostate, breast | SMR: 0.85  95% CI (0.77-0.94) | Male breast Thyroid | * |
| Ma, Lee, Fleming, and Dosemeci (1998) | Colorado, Georgia, Idaho, Indiana, Kansas, Kentucky, Maine, Missouri, Nebraska, Nevada, New Hampshire, New Jersey, New Mexico, North Carolina, Ohio, Oklahoma, Rhode Island, South Carolina, Tennessee, Utah, Vermont, Washington, West Virginia, and Wisconsin 1984-1993 | All, lip, salivary glands, nasopharynx, esophagus, stomach, colon, rectum, liver, biliary tract, pancreas, nasal cavity, larynx, bronchus and lung, pleura, bones and joints, soft tissue sarcoma, melanoma of skin, other skin, prostate, testis, bladder, kidney and renal pelvis, ureter, brain and central nervous system, thyroid glands, non-Hodgkin's lymphoma, hodgkin's disease, multiple myeloma, leukemia 204-208 | MOR: 1.1  95% CI (1.1­-1.2) | *White FF:*  Prostate, lip, pancreas, soft tissue sarcoma, melanoma, kidney and renal pelvis, bronchus and lung cancer, non-Hodgkin's lymphoma *Black FF:*  Prostate, brain and central nervous system cancer, colon, and nasopharynx. | * |
| Musk, Monson, Peters, and Peters (1978) | Boston 1915-1975 | All cancers 140-208digestive 150-159respiratory 160-165genitourinary 185-189brain and other central nervous system 191-192lymphatic and haemopoietic 200-208other 140-149, 166-184, 190, 193-199 | SMR: 86** | * | * |
| K. U. Petersen, Pedersen, Bonde, Ebbehøj, and Hansen (2018) | Denmark  1970-2014 | Oral cavity and esophagus, stomach, intestines, except rectum, rectum, larynx, trachea and lungs, bones and skin, prostate, lymphatic and blood forming tissues, other | SMR: 0.99  95% CI (0.89–1.09) | *Full time FF:*  Stomach *Part-time/volunteer FF:*  Prostate | * |
| Rosenstock, Demers, Heyer, and Barnhart (1990) | Portland, Seattle, Tacoma 1945-1984 | Trachea, bronchus, and lung | SMR: 109  95% CI (72-155) | * | * |
| Jeavana Sritharan et al. (2018) | Canada  1991-2010 | Prostate | HR: 1.17  95% CI (1.01–1.36) | * | * |
| Tornling et al. (1994) | Stockholm, Sweden 1951-1986 | All, stomach, colon, rectum and anus, liver, pancreas, bronchus and lung, melanoma, other skin, prostate, kidney, brain, all hematomietic 200-208 | SMR: 82  95% CI (72-91) | Stomach  Brain | * |
| Vena and Fiedler (1987) | Buffalo, New York  1950-1979 | Digestive organs and peritoneum , esophagus, stomach, colon, rectum, liver, pancreas, respiratory system, prostate, bladder, kidney, brain and other CNS, lymphatic and hematopoietic tissue | SMR: 1.09 95% CI (0.89- 1.32) | Colon  Bladder | * |
| Wende (1996) | Buffalo, New York  1950-1982 | Cancer of buccal cavity and pharynx, digestive organs and peritoneum, esophagus, stomach, large intestine, rectum, liver - primary only, pancreas, respiratory system, larynx , lung - primary and secondary, bone, skin, prostate, testis , bladder, kidney, eye, brain and other central nervous system, thyroid, Lymphosarcoma and reticulosarcoma, Hodgkin's disease, Leukemia and aleukemia, other lymphatic tissue, all lymphopoietic cancer | SMR: 1.08  95% CI (0.92-1.26) | Large intestine Rectum Bladder Digestive organs Peritoneum | * |
| Muegge et al. (2018) | Indiana  1985-2013 | All cancers, buccal cavity and pharynx, other parts of the buccal cavity, pharynx, pancreas, kidney, other and unspecified sites, connective tissue, brain and other parts of the nervous system | OR: 1.19  95% CI (1.08-1.30) | Buccal cavity Pharynx and other parts of the buccal cavity Pancreas kidney, Connective tissue Brain and other parts of the nervous system  Unspecified sites | * |
| Berg (1975) | U.S. and U.K.  1949-1963 | Colonic and rectal, subtypes bowel cancer | SMR: 279** | * | * |
| Burnett, Halperin, Lalich, and Sestito (1994) | Alaska, Colorado, Georgia, Idaho, Indiana. Kansas, Kentucky, Maine, Missouri, Nebraska, Nevada, New Hampshire, New Jersey, New Mexico, New York except New York City, North Carolina, Ohio, Oklahoma, Pennsylvania, Rhode Island, South Carolina, Tennessee, Utah, Vermont, Washington, West, Virginia, and Wisconsin.  1984-1990 | Rectum,lung , skin, bladder, kidney, brain and nervous system, lymphatic and hematopoetic, non-Hodgkin's lymphomas, multiple myeloma, leukemia | PMR 110 95% CI (106-114) | Lymphatic and hematopoetic Non-Hodgkin’s lymphomas Multiple myeloma Kidney Skin Rectum | * |
| Eliopulos, Armstrong, Spickett, and Heyworth (1984) | Western Australia  1939-1978 | Respiratory, stomach, intestinal, other digestive cancer, genitourinary cancer and lymphohaematopoietic cancer | SMR 1.09 95% CI (0.74-1.56) | * | * |
| Heyer, Weiss, Demers, and Rosenstock (1990) | Seattle, Washington  1945-1983 | All, digestive, esophagus, stomach, intestine, rectal, respiratory, lung, brain and nervous system, benign or unspecified brain, lymphohaematopoetic, leukemia, other lymphohaematopoetic | SMR:96  95% CI (77-118) | Leukemia Lung Other lymphatic/hematopoietic | * |
| Mastromatteo (1959) | Ontario, Canada  1921-1953 | All cancers and other malignant tumors | P <0.001** | * | * |
| Ernester et al. (1979) | Alameda and San Francisco, California  1968-1972 | Prostate | OR: 3.0** | * | * |
| Coggon, Harris, Brown, Rice, and Palmer (2009) | United Kingdom and Wales  1991-2000 | Peritoneum, pleura, non-melanoma cancer of the skin | PMR: 174 95% CI (116-251) | Pleura  Non-Melanoma of the skin | * |
| Glass et al. (2019) | Australia  1998-2011 | All, lip, oral cavity and pharynx, digestive organs, colorectal, colon, rectum, respiratory, lung, melanoma, mesothelioma/pleura, breast, female reproductive, cervix, urinary tract, kidney, brain and other cns, brain, thyroid and other endocrine, thyroid, unknown site, lymphohematopoetic, non-hodgkin's lymphoma, myeloma, leukemia, all other cancers | *Volunteer Female FF:* SMR: 0.75  95% CI (0.66-0.84) | All cancers combined | * |
| Blair, Walrath, and Rogot (1985) | United States 1954-1970 | Intestines, lung and bronchus (excludes trachea) | SMR 141  P<0.05 | * | * |
| Krstev (1998) | Colorado, Georgia, Idaho, Indi-ana, Kansas, Kentucky, Maine, Missouri, Nebraska, Nevada,New Hampshire, New Jersey, New Mexico, North Carolina,Ohio, Oklahoma, Rhode Island, South Carolina, Tennessee,Utah, Washington, West Virginia, Wisconsin, Vermont 1984-1993 | Prostate | MOR: 1.2 95%CI (1.0–1.4) | Prostate | * |
| Dolin (1992) | U.K.  1965- 1980 | Bladder 188 | SMR; 79  95% CI(16-231) | * | * |
| Pion (1995) | U.S.  1982-1988 | Subtypes malignant melanoma | OR 2.29  95% CI (0.85-6.16) | * | * |
| McDowell (1986) | U.K.  1971-1980 | Testicular | RR: 0.59 95% CI (61-43) | * | Testicular |
| Zhao G, Erazo B, Ronda E, Brocal F, Regidor E (2020). | Spain  2001-2011 | All cancers; mouth and pharynx, hypopharynx, esophagus, stomach, colon, rectum, liver, pancreas, larynx, lung, bone, melanoma, mesotheliom, breast, prostate, kidney, renal pelvis, bladder, cns, thyroid, lyphomas, hodgkin's, lymphoma, leukemia, all other cancers | MMR 1.00  95% CI (0.89–1.12) | Hypopharynx Renal Pelvis Larynx cancer | Pancreas |
| Pinkerton L, Bertke SJ, Yiin J, Dahm M, Kubale T, Hales T, Purdue M, Beaumont JJ, Daniels R (2020). | United States – San Francisco, Chicago, Philadelphia  1950-2016 | All cancers, esophagus, stomach, intestine, rectum, lung, breast, prostate, other male genital, kidney, bladder, skin, mesothelioma, brain, non-hodgkin lymphoma, leukemia, multiple myeloma | SMR: 1.12  95% CI (1.08 to 1.16) | Mesothelioma Non-Hodgkin's lymphoma  Esophagus Intestine Rectum Lung Kidney | * |

*Statistically increase/decrease not found.

** Overall risk not calculated.

***Fire departments in Chicago (CFD), Philadelphia (PFD) and San Francisco (SFFD); Fire Department of the City of New York (FDNY), Career Firefighter Health Study (CFHS).

| **Papers Excluded due to Temporal and Geographical Overlap** | | | | | |
| --- | --- | --- | --- | --- | --- |
| **Study ID** | **Study Reference** | **Incidence (I) or Mortality (M)** | **Enrollment Period** | **Population** | **Overlap** |
| BarisGT2001 | Baris D, Garrity TJ, Telles JL, Heineman EF, Olshan A, Zahm SH. Cohort mortality study of Philadelphia firefighters. Am J Ind Med. 2001;39(5):463-476. doi:10.1002/ajim.1040 | Mortality | 1925-1986 | Philadelphia | Excluded due to geographical overlap with PinkertonBY2020 |
| Bates 2007 | Bates MN. Registry-based case-control study of cancer in California firefighters. Am J Ind Med. 2007;50(5):339-344. doi:10.1002/ajim.20446 | Incidence | 1983-2003 | California | Excluded due to geographical overlap with BeaumontCJ1991 |
| DanielsBD2015 | Daniels RD, Bertke S, Dahm MM, et al. Exposure-response relationships for select cancer and non-cancer health outcomes in a cohort of U.S. firefighters from San Francisco, Chicago and Philadelphia (1950-2009). Occup Environ Med. 2015;72(10):699-706. doi:10.1136/oemed-2014-102671 | Incidence | 1950-2009 | San Francisco, Chicago, Philadelphia | Excluded due to geographical and years of overlap with PinkertonBY2020 |
| DanielsKY2014 | Daniels RD, Kubale TL, Yiin JH, et al. Mortality and cancer incidence in a pooled cohort of US firefighters from San Francisco, Chicago and Philadelphia (1950-2009). Occup Environ Med. 2014;71(6):388-397. doi:10.1136/oemed-2013-101662 | Both | 1950-2009 | California, Chicago, Philadelphia | Excluded due to geographical overlap with PinkertonBY2020 |
| DeschampsMF1995 | Deschamps S, Momas I, Festy B. Mortality amongst Paris fire-fighters. Eur J Epidemiol. 1995;11(6):643-646. doi:10.1007/BF01720297 | Mortality | 1977-1991 | Paris | Excluded due to geographical overlap with AmadeoMM2015 |
| GreeneKS2008 | Greene, Kirsten & Konety, Badrinath & Stoller, Marshall. (2008). Results from the San Francisco Firefighters bladder cancer screening study. Journal of Urology - J UROL. 179. 323-323. 10.1016/S0022-5347(08)60945-3 | Incidence | 2006-2007 | San Francisco | Excluded due to years of overlap with PinkertonBY2020 |
| Hansen1990 | Hansen ES. A cohort study on the mortality of firefighters. Br J Ind Med. 1990;47(12):805-809. doi:10.1136/oem.47.12.805 | Mortality | 1970-1980 | Denmark | Excluded due to geographical overlap with PetersenPB2018 |
| KrishnanFC2003 | Krishnan G, Felini M, Carozza SE, Miike R, Chew T, Wrensch M. Occupation and adult gliomas in the San Francisco Bay Area. J Occup Environ Med. 2003;45(6):639-647. doi:10.1097/01.jom.0000069245.06498.48 | Incidence | 1991-1994 & 1997-1999 | San Francisco | Excluded due to years of overlap with PinkertonBY2020 |
| KullbergAG2018 | Kullberg C, Andersson T, Gustavsson P, Selander J, Tornling G, Gustavsson A, Bigert C. Cancer incidence in Stockholm firefighters 1958-2012: an updated cohort study. Int Arch Occup Environ Health. 2018 Apr;91(3):285-291. doi: 10.1007/s00420-017-1276-1. Epub 2017 Nov 21. PMID: 29164319; PMCID: PMC5845066. | Incidence | 1958-2012 | Stockholm | Exclude due to geographical overlap with PukkalaMW2014 |
| MaFL2006 | Ma F, Fleming LE, Lee DJ, Trapido E, Gerace TA. Cancer incidence in Florida professional firefighters, 1981 to 1999. J Occup Environ Med. 2006;48(9):883-888. doi:10.1097/01.jom.0000235862.12518.04 | Incidence | 1981-1999 | Florida | Excluded due to geographical overlap with MaFL2005 |
| MoirZD2016 | Moir W, Zeig-Owens R, Daniels RD, et al. Post-9/11 cancer incidence in World Trade Center-exposed New York City firefighters as compared to a pooled cohort of firefighters from San Francisco, Chicago and Philadelphia (9/11/2001-2009). Am J Ind Med. 2016;59(9):722-730. doi:10.1002/ajim.22635 | Incidence | 2001-2009 | New York City | Excluded due to overlap with WebberSZ2021 |
| TornlinGH1994 | Tornling G, Gustavsson P, Hogstedt C. Mortality and cancer incidence in Stockholm fire fighters. Am J Ind Med. 1994;25(2):219-228. doi:10.1002/ajim.4700250208 | Both | 1931-1983 | Stockholm | Excluded Incidence due to geographical overlap with PukkalaMW2014 |
| VenaF1987 | Vena JE, Fiedler RC. Mortality of a municipal-worker cohort: IV. Fire fighters. Am J Ind Med. 1987;11(6):671-684. doi:10.1002/ajim.4700110608 | Mortality | 1950-1979 | Buffalo | Excluded due to geographical overlap with Wende1996 |
| Bates1995 | Bates MN, Lane L. Testicular cancer in fire fighters: a cluster investigation. N Z Med J. 1995;108(1006):334-337. | Incidence | 1980-1991 | New Zealand | Excluded due to geographical overlap with BatesFG2001 |
| HeyerWD1990 | Heyer N, Weiss NS, Demers P, Rosenstock L. Cohort mortality study of Seattle fire fighters: 1945-1983. Am J Ind Med. 1990;17(4):493-504. doi:10.1002/ajim.4700170407 | Mortality | 1945-1983 | Seattle | Excluded due to geographical overlap with RosenstockDH1990 and DemersHR1994 |
| DemersMW2011 | Demers P, Martinsen JI, Weiderpass E, et al Cancer incidence among Nordic firefighters Occupational and Environmental Medicine 2011;68:A19-A20. | Incidence | 1960, 1970, 1980, and 1990 censuses | Denmark, Finland, Iceland, Norway, Sweden | Excluded due to geographical overlap with PukkalaMW2014 and MarjerrisonJG2022 |
| AlguacilPG2003 | Alguacil J, Pollán M, Gustavsson P. Occupations with increased risk of pancreatic cancer in the Swedish population. Occup Environ Med. 2003;60(8):570-576. doi:10.1136/oem.60.8.570 | Incidence | 1960 and 1970 censuses | Sweden | Exclude due to geographical overlap with PukkalaMW2014 |
| CarozzaWM2000 | Carozza SE, Wrensch M, Miike R, et al. Occupation and adult gliomas. Am J Epidemiol. 2000;152(9):838-846. doi:10.1093/aje/152.9.838 | Incidence | 1991-1994 | 6 San Francisco Bay area counties (Alamada, Contra Costa, Marin, San Mateo, San Franciso, Santa Clara) | Excluded due to geographical overlap with BeaumontCJ1991 |
| GuenelEL1990 | Guenel P, Engholm G, Lynge E. Laryngeal cancer in Denmark: a nationwide longitudinal study based on register linkage data. Br J Ind Med. 1990;47(7):473-479. doi:10.1136/oem.47.7.473 | Incidence | 1970 census, FU through 1980 | Denmark | Excluded due to geographical overlap with PetersenPB2018 |

**References**

*Bold references for the papers included in the final analysis (k=38), which excluded case control designs (k=31), and duplicate reports based on geographical and temporal overlap (k=19).

***Ahn, Y. S., & Jeong, K. S. (2015). Mortality due to malignant and non-malignant diseases in Korean professional emergency responders. *PLoS One, 10*(3), 1-14. doi:10.1371/journal.pone.0120305**

***Ahn, Y. S., Jeong, K. S., & Kim, K. S. (2012). Cancer morbidity of professional emergency responders in Korea. *Am J Ind Med, 55*(9), 768-778. doi:10.1002/ajim.22068**

Alguacil J, P. M., Gustavsson P. (2003). Occupations with increased risk of pancreatic cancer in the Swedish population. *Occupational and Environmental Medicine*, 570–576. doi:10.1136/oem.60.8.570

***Amadeo, B., Marchand, J.-L., Moisan, F., Donnadieu, S., Gaëlle, C., Simone, M.-P., . . . Brochard, P. (2015). French firefighter mortality: analysis over a 30-year period. *American Journal of Industrial Medicine, 58*(4), 437-443. doi:10.1002/ajim.22434**

***Aronson, K. J., Tomlinson, G. A. and Smith, L. (1994). Mortality among fire fighters in metropolitan Toronto. *American Journal of Industrial Medicine, 26*(1), 89-101. doi:10.1002/ajim.4700260108**

Band PR, Le ND, Fang R, Gallagher R. Identification of occupational cancer risks in British Columbia: a population-based case-control study of 769 cases of non-Hodgkin's lymphoma analyzed by histopathology subtypes. *J Occup Environ Med*. 2004;46(5):479-489. doi:10.1097/01.jom.0000126028.99599.36

Baris, D., Garrity, T. J., Telles, J. L., Heineman, E. F., Olshan, A., & Zahm, S. H. (2001). Cohort mortality study of Philadelphia firefighters. *American Journal of Industrial Medicine, 39*(5), 463-476. doi:<https://doi.org/10.1002/ajim.1040>

Bates, M. N. (2007). Registry-based case–control study of cancer in California firefighters. *American Journal of Industrial Medicine, 50*(5), 339-344. doi:<https://doi.org/10.1002/ajim.20446>

***Bates, M. N., Fawcett, J., Garrett, N., Arnold, R., Pearce, N., & Woodward, A. (2001). Is testicular cancer an occupational disease of fire fighters? *American Journal of Industrial Medicine, 40*(3), 263-270. doi:10.1002/ajim.1097**

Bates, M. N., & Lane, L. (1995). Testicular cancer in fire fighters: a cluster investigation. *The New Zealand Medical Journal, 108*(1006), 334-337.

***Beaumont, J. J., Chu, G. S., Jones, J. R., Schenker, M. B., Singleton, J. A., Piantanida, L. G., & Reiterman, M. (1991). An epidemiologic study of cancer and other causes of mortality in San Francisco firefighters. *American Journal of Industrial Medicine, 19*(3), 357-372. doi:10.1002/ajim.4700190309**

***Berg, J. W., & Howell, M. A. (1975). Occupation and bowel cancer. *Journal of Toxicology and Environmental Health, 1*(1), 75-89. doi:10.1080/15287397509529309**

***Bigert, C., Martinsen, J. I., Gustavsson, P., & Sparén, P. (2020). Cancer incidence among Swedish firefighters: an extended follow-up of the NOCCA study. *Int Arch Occup Environ Health, 93*(2), 197-204. doi:10.1007/s00420-019-01472-x**

***Blair, A., Walrath, J., & Rogot, E. (Mortality patterns among U.S. veterans by occupation. I. Cancer). 1985. *Journal of the National Cancer Institute, 75*(6), 1039-1047.**

Burns PB, Swanson GM. The Occupational Cancer Incidence Surveillance Study (OCISS): risk of lung cancer by usual occupation and industry in the Detroit metropolitan area. *Am J Ind Med*. 1991;19(5):655-671. doi:10.1002/ajim.4700190510

Burnett, C. A., Halperin, W. E., Lalich, N. R., & Sestito, J. P. (1994). Mortality among firefighters: a 27 state survey. *American Journal of Industrial Medicine, 26*, 831-833.

Carozza SE, Wrensch M, Miike R, et al. Occupation and adult gliomas. Am J Epidemiol. 2000;152(9):838-846. doi:10.1093/aje/152.9.838.

Coggon, D., Harris, E. C., Brown, T., Rice, S., & Palmer, K. T. (2009). Occupational mortality in England and Wales, 1991-2000. *Office for National Statistics*. Retrieved from <https://www.legislation.gov.uk/>

Corbin, M., McLean, D., Mannetje, A., Dryson, E., Walls, C., McKenzie, F., . . . Pearce, N. (2011). Lung cancer and occupation: A New Zealand cancer registry-based case–control study. *American Journal of Industrial Medicine, 54*(2), 89-101. doi:10.1002/ajim.20906

Daniels, R. D., Bertke, S., Dahm, M. M., Yiin, J. H., Kubale, T. L., Hales, T. R., . . . Pinkerton, L. E. (2015). Exposure-response relationships for select cancer and non-cancer health outcomes in a cohort of U.S. firefighters from San Francisco, Chicago and Philadelphia (1950-2009). *Occupational and Environmental Medicine, 72*(10), 699-706. doi:0.1136/oemed-2014-102671

Daniels, R. D., Kubale, T. L., Yiin, J. H., Dahm, M. M., Hales, T. R., Baris, D., . . . Pinkerton, L. E. (2014). Mortality and cancer incidence in a pooled cohort of US firefighters from San Francisco, Chicago and Philadelphia (1950-2009). *Occup Environ Med, 71*(6), 388-397. doi:10.1136/oemed-2013-101662

Delahunt, B., Bethwaite, P. B., & Nacey, J. N. (1995). Occupational risk for renal cell carcinoma. A case-control study based on the New Zealand Cancer Registry. *British Journal of Urology: Official Journal of the British Association of Urological Surgeons, 75*(5), 578-582. doi:10.1111/j.1464-410x.1995.tb07410.x

Demers, P., Martinsen, J. I., Weiderpass, E., Kjærheim, K., Lynge, E., Sparén, P., & Pukkala, E. (2011). Cancer incidence among Nordic firefighters. *Occupational and Environmental Medicine, 68*(Suppl 1), A19-A20. doi:10.1136/oemed-2011-100382.60

***Demers, P. A., Checkoway, H., Vaughan, T. L., Weiss, N. S., Heyer, N. J., & Rosenstock, L. (1994). Cancer incidence among firefighters in Seattle and Tacoma, Washington (United States). *Cancer Causes & Control, 5*(2), 129-135. doi:10.1007/BF01830258**

Deschamps, S., Momas, I., & Festy, B. (1995). Mortality Amongst Paris Fire-Fighters. *European Journal of Epidemiology, 11*(6), 643-646. Retrieved from <https://www.jstor.org/stable/3582167>

De Roos, A. J., Stewart, P. A., Linet, M. S., Heineman, E. F., Dosemeci, M., Wilcosky, T., Shapiro, W. R., Selker, R. G., Fine, H. A., Black, P. M., & Inskip, P. D. (2003). Occupation and the Risk of Adult Glioma in the United States. *Cancer Causes & Control*, *14*(2), 139–150. http://www.jstor.org/stable/3553626

***Dolin, P. J., & Cook-Mozaffari, P. (1992). Occupation and bladder cancer: a death-certificate study. *British Journal of Cancer, 66*(3), 568-578. doi:10.1038/bjc.1992.316**

Elci, O. C., Akpinar-Elci, M., Alavanja, M., & Dosemeci, M. (2003). Occupation and the risk of lung cancer by histologic types and morphologic distribution: A case control study in Turkey. *Monaldi Archives for Chest Disease - Pulmonary Series, 59*(3), 183-188.

***Eliopulos, E., Armstrong, B. K., Spickett, J. T., & Heyworth, F. (1984). Mortality of firefighters in Western Australia. *British Journal of Industrial Medicine, 41*, 183-187. doi:10.1136/oem.41.2.183**

Ernester, V. L., Selvin, S., Brown, S. M., Sacks, S. T., Winkelstein Jr., W., & Austin, D. F. (1979). Occupation and prostatic cancer. A review and retrospective analysis based on death certificates in two California counties. *Journal of Occupational and Environmental Medicine, 21*(3), 175-183.

Fang, R., Le, N., & Band, P. (2011). Identification of occupational cancer risks in British Columbia, Canada: a population-based case—control study of 1,155 cases of colon cancer. *International Journal of Environmental Research and Public Health, 8*(10), 3821-3843. doi:10.3390/ijerph8103821

Feuer, E., & Rosenman, K. (1986). Mortality in police and firefighters in New Jersey. *American Journal of Industrial Medicine, 9*(6), 517-527. doi:10.1002/ajim.4700090603

Figgs, L. W., Dosemeci, M., & Blair, A. (1995). United States non-Hodgkin's lymphoma surveillance by occupation 1984-1989: a twenty-four state death certificate study. *American Journal of Industrial Medicine, 27*(6), 817-835. doi:10.1002/ajim.4700270606

***Firth, H. M., Cooke, K. R., & Herbison, G. P. (1996). Male cancer incidence by occupation: New Zealand, 1972-1984. *International Journal of Epidemiology, 25*(1), 14-21. doi:10.1093/ije/25.1.14**

Gaertner, R. R., Trpeski, L., & Johnson, K. C. (2004). A case-control study of occupational risk factors for bladder cancer in Canada. *Cancer Causes & Control, 15*(10), 1007-1019. doi:10.1007/s10552-004-1448-7

***Giles, G., Staples, M., & Berry, J. (1993). *Cancer incidence in Melbourne Metropolitan Fire Brigade members, 1980 - 1989*. Retrieved from Canada: Retrieved from** [**https://www.ncbi.nlm.nih.gov/pubmed/8334236**](https://www.ncbi.nlm.nih.gov/pubmed/8334236)

***Glass, D. C., Del Monaco, A., Pircher, S., Vander Hoorn, S., & Sim, M. R. (2016). Mortality and cancer incidence at a fire training college. *Occupational Medicine, 66*(7), 536-542. doi:oi:10.1093/occmed/kqw**

***Glass, D. C., Del Monaco, A., Pircher, S., Vander Hoorn, S., & Sim, M. R. (2017). Mortality and cancer incidence among male volunteer Australian firefighters. *Occupational and Environmental Medicine, 74*(9), 628-638. doi:10.1136/oemed-2016-104088**

***Glass, D. C., Del Monaco, A., Pricher, S., Vander Hoorn, S., & Sim, M. R. (2019). Mortality and cancer incidence among female Australian firefighters. *Occupational and Environmental Medicine, 0*, 1-7. doi:10.1136/oemed-2018-105336**

***Glass, D. C., Pircher, S., Del Monaco, A., Hoorn, S. V., & Sim, M. R. (2016). Mortality and cancer incidence in a cohort of male paid Australian firefighters. *Occup Environ Med, 73*(11), 761-771. doi:10.1136/oemed-2015-103467**

Greene, K., Konety, B., & Stoller, M. (2008). Results from the San Francisco Firefighters bladder cancer screening study. *Journal of Urology, 179*(4), 323-323. doi:10.1016/S0022-5347(08)60945-3

Goodman KJ, Bible ML, London S, Mack TM. Proportional melanoma incidence and occupation among white males in Los Angeles County (California, United States). *Cancer Causes Control*. 1995;6(5):451-459. doi:10.1007/BF00052186

Grimes, G., Hirsch, D. and Borgeson, D. (1991). Risk of death among Honolulu fire fighters. *Hawaii Medical Journal, 50*(3), 82-85.

Guenel, P., Engholm, G., & Lynge, E. (1990). Laryngeal cancer in Denmark: a nationwide longitudinal study based on register linkage data. *Br J Ind Med, 47*(7), 473-479. doi:10.1136/oem.47.7.473

***Guidotti, T. L. (1993). Mortality of urban firefighters in Alberta, 1927-1987. *American Journal of Industrial Medicine, 23*(6), 921-940. doi:10.1002/ajim.4700230608**

Hansen, E. S. (1990). A cohort study on the mortality of firefighters. *British Journal of Industrial Medicine, 47*(12), 805-809. doi:10.1136/oem.47.12.805

Harris, M. A., Kirkham, T. L., MacLeod, J. S., Tjepkema, M., Peters, P. A., & Demers, P. A. (2018). Surveillance of cancer risks for firefighters, police, and armed forces among men in a Canadian census cohort. *American Journal of Industrial Medicine, 61*(10), 815-823. doi:10.1002/ajim.22891

Heyer, N., Weiss, N. S., Demers, P., & Rosenstock, L. (1990). Cohort mortality study of Seattle Fire Fighters: 1945 - 1983. *American Journal of Industrial Medicine, 17*, 493 -504. doi:10.1002/ajim.4700170407

Huebner WW, Schoenberg JB, Kelsey JL, et al. Oral and pharyngeal cancer and occupation: a case-control study. *Epidemiology*. 1992;3(4):300-309. doi:10.1097/00001648-199207000-00005

***Ide, C. W. (2014). Cancer incidence and mortality in serving whole-time Scottish firefighters 1984-2005. *Occup Med (Lond), 64*(6), 421-427. doi:10.1093/occmed/kqu080**

Kang, D., Davis, L. K., Hunt, P., & Kriebel, D. (2008). Cancer incidence among male Massachusetts firefighters, 1987-2003. *Am J Ind Med, 51*(5), 329-335. doi:10.1002/ajim.20549

Karami, S., Colt, J. S., Schwartz, K., Davis, F. G., Ruterbusch, J. J., Munuo, S. S., . . . Purdue, M. P. (2012). A case–control study of occupation/industry and renal cell carcinoma risk. *BMC Cancer, 12*, 2- 12. doi:10.1186/1471-2407-12-344

Krishnan, G., Felini, M., Carozza, S. E., Miike, R., Chew, T., & Wrensch, M. (2003). Occupation and adult gliomas in the San Francisco Bay Area. *Journal of Occupational and Environmental Medicine, 45*(6), 639-647. doi:10.1097/01.jom.0000069245.06498.48

Krstev, S., Baris, D., Stewart, P., Dosemeci, M., Swanson, G.M., Greenberg, R.S., Schoenberg, J.B., Schwartz, A.G., Liff, J.M. and Hayes, R.B. (1998), Occupational risk factors and prostate cancer in U.S. Blacks and Whites. Am. J. Ind. Med., 34: 421-430. https://doi.org/10.1002/(SICI)1097-0274(199811)34:5<421::AID-AJIM2>3.0.CO;2-T

Krstev S, Baris D, Stewart PA, Hayes RB, Blair A, Dosemeci M. Risk for prostate cancer by occupation and industry: a 24-state death certificate study. *Am J Ind Med*. 1998;34(5):413-420. doi:10.1002/(sici)1097-0274(199811)34:5<413::aid-ajim1>3.0.co;2-r

Kullberg, C., Andersson, T., Gustavsson, P., Selander, J., Tornling, G., Gustavsson, A., & Bigert, C. (2018). Cancer incidence in Stockholm firefighters 1958-2012: An updated cohort study. International Archives of Occupational and Environmental Health, 9(3), 285-291. doi:10.1007/s00420-017-1276-1

Lee DJ, Koru-Sengul T, Hernandez MN, et al. Cancer risk among career male and female Florida firefighters: Evidence from the Florida Firefighter Cancer Registry (1981-2014). *Am J Ind Med*. 2020;63(4):285-299. doi:10.1002/ajim.23086

***Lenahan, P., Gochfeld, M., Meng, Q., Robson, M., & Fagliano, J. (2018). *A 30-year study of cancer incidence in firefighters and police officers in New Jersey's four largest municipalities*. Dissertation. Graduate Program in Public Health. Rutgers, The State University of New Jersey. New Jersey.**

Ma, F., Fleming, L. E., Lee, D. J., Trapido, E., & Gerace, T. A. (2006). Cancer incidence in Florida professional firefighters, 1981 to 1999. *Journal of Occupational and Environmental Medicine, 48*(9), 883-888. doi:10.1097/01.jom.0000235862.12518.04

***Ma, F., Fleming, L. E., Lee, D. J., Trapido, E., Gerace, T. A., Lai, H., & Lai, S. (2005). Mortality in Florida professional firefighters, 1972 to 1999. *Am J Ind Med, 47*(6), 509-517. doi:10.1002/ajim.20160**

Ma, F., Lee, D. J., Fleming, L. E., & Dosemeci, M. (1998). Race-specific cancer mortality in US firefighters: 1984-1993. *Journal of Occupational and Environmental Medicine, 40*(12), 1134-1138. doi:10.1097/00043764-199812000-00014

***Marjerrison, N., Jakobsen, J., Grimsrud, T. K., Hansen, J., Martinsen, J. I., Nordby, K. C., . . . Kjærheim, K. (2022). Cancer incidence in sites potentially related to occupational exposures: 58 years of follow-up of firefighters in the Norwegian Fire Departments Cohort. *Scand J Work Environ Health*. doi:10.5271/sjweh.4009**

***Mastromatteo, E. (1959). Mortality in city firemen, II: A study of mortality in firemen of a city fire department. *A.M.A. Archives of Industrial Health, 20*, 227-233.**

***McDowall, M. E., & Balarajan, R. (1986). Testicular cancer mortality in England and Wales 1971-80: variations by occupation. *Journal of Epidemiology and Community Health, 40*, 26-29. doi:10.1136/jech.40.1.26**

Moir, W., Zeig-Owens, R., Daniels, R. D., Hall, C. B., Webber, M. P., Jaber, N., . . . Prezant, D. J. (2016). Post-9/11 cancer incidence in World Trade Center-exposed New York City firefighters as compared to a pooled cohort of firefighters from San Francisco, Chicago and Philadelphia (9/11/2001-2009). *American Journal of Industrial Medicine, 59*(9), 722-730. doi:10.1002/ajim.22635

***Morton, W., & Marjanovic, D. (1984). Leukemia incidence by occupation in the Portland- Vancouver metropolitan area. *American Journal of Industrial Medicine, 6*(3), 185-205. doi:10.1002/ajim.4700060304**

Muegge, C. M., Zollinger, T. W., Song, Y., Wessel, J., Monahan, P. O., & Moffatt, S. M. (2018). Excess mortality among Indiana firefighters, 1985-2013. *American Journal of Industrial Medicine, 61*(12), 961-967. doi:10.1002/ajim.22918

***Musk, A. W., Monson, R. R., Peters, J. M., & Peters, R. K. (1978). Mortality among Boston firefighters, 1915--1975. *Br J Ind Med, 35*(2), 104-108. doi:10.1136/oem.35.2.104**

Paget-Bailly, S., Guida, F., Carton, M., Menvielle, G., Radoi, L., Cyr, D., . . . Luce, D. (2013). Occupation and head and neck cancer risk in men: Results from the ICARE study, a French population-based case-control study. *Journal of Occupational and Environmental Medicine, 55*(9), 1065-1073. doi:10.1097/JOM.0b013e318298fae4

***Petersen, K. K. U., Pedersen, J. E., Bonde, J. P., Ebbehoej, N. E., & Hansen, J. (2018). Long-term follow-up for cancer incidence in a cohort of Danish firefighters. *Occupational and Environmental Medicine, 75*(4), 263-269. doi:10.1136/oemed-2017-104660**

***Petersen, K. U., Pedersen, J. E., Bonde, J. P., Ebbehøj, N. E., & Hansen, J. (2018). Mortality in a cohort of Danish firefighters; 1970-2014. *Int Arch Occup Environ Health, 91*(6), 759-766. doi:10.1007/s00420-018-1323-6**

***Pinkerton, L., Bertke, S. J., Yiin, J., Dahm, M., Kubale, T., Hales, T., . . . Daniels, R. (2020). Mortality in a cohort of US firefighters from San Francisco, Chicago and Philadelphia: an update. *Occup Environ Med, 77*(2), 84-93. doi:10.1136/oemed-2019-105962**

***Pion, I. A., Rigel, D. S., Garfinkel, L., Silverman, M. K., & Kopf, A. W. (1995). Occupation and the risk of malignant melanoma. *CANCER Supplement, 75*(2), 637-644. doi:10.1002/1097-0142(19950115)75:2+<637::aid-cncr2820751404>3.0.co;2-#**

***Pukkala, E., Martinsen, J. I., Weiderpass, E., Kjaerheim, K., Lynge, E., Tryggvadottir, L., . . . Demers, P. A. (2014). Cancer incidence among firefighters: 45 years of follow-up in five Nordic countries. *Occup Environ Med, 71*(6), 398-404. doi:10.1136/oemed-2013-101803**

***Rosenstock, L., Demers, P., Heyer, N. J., & Barnhart, S. (1990). Respiratory mortality among firefighters. *British Journal of Industrial Medicine, 47*(7), 462-465. doi:10.1136/oem.47.7.462**

Sama, S. R., Martin, T. R., Davis, L. K., & Kriebel, D. (1990). Cancer incidence among Massachusetts firefighters, 1982-1986. *American Journal of Industrial Medicine, 18*(1).

Sritharan, J., Demers, P. A., Harris, S. A., Cole, D. C., Peters, C. E., The Canadian Cancer Registries Epidemiology Research Group, & Villeneuve, P. J. (2017). Occupation and risk of prostate cancer in Canadian men: A case-control study across eight Canadian provinces. *Cancer Epidemiology, 48*, 96-103. doi:10.1016/j.canep.2017.04.006

Sritharan, J., MacLeod, J., Harris, S., Cole, D. C., Harris, A., Tjepkema, M., . . . Demers, P. A. (2018). Prostate cancer surveillance by occupation and industry: the Canadian Census Health and Environment Cohort (CanCHEC). *Cancer Medicine, 7*(4), 1468-1478. doi:10.1002/cam4.1358

Sritharan J, MacLeod JS, McLeod CB, Peter A, Demers PA. Prostate cancer risk by occupation in the Occupational Disease Surveillance System (ODSS) in Ontario, Canada. Risque de cancer de la prostate par profession dans le Système de surveillance des maladies professionnelles de l’Ontario, Canada. *Health Promot Chronic Dis Prev Can*. 2019;39(5):178-186. doi:10.24095/hpcdp.39.5.02.

Stang, A., Jockel, K. H., Baumgardt-Elms, C., & Ahrens, W. (2003). Firefighting and risk of testicular cancer: results from a German population-based case-control study. *American Journal of Industrial Medicine, 43*(3), 291-294. doi:10.1002/ajim.10178

Tornling, G. r., Gustavsson, P., & Hogstedt, C. (1994). Mortality and cancer incidence in stockholm fire fighters. *American Journal of Industrial Medicine, 25*(2), 219-228. doi:<https://doi.org/10.1002/ajim.4700250208>

Tsai, R. J., Luckhaupt, S. E., Schumacher, P., Cress, R. D., Deapen, D. M., & Calvert, G. M. (2015). Risk of cancer among firefighters in California, 1988–2007. *American Journal of Industrial Medicine, 58*(7), 715-729. doi:<https://doi.org/10.1002/ajim.22466>

Vena, J. E., & Fiedler, R. C. (1987). Mortality of a municipal-worker cohort: IV. Fire fighters. *American Journal of Industrial Medicine, 11*(6), 671-684. doi:10.1002/ajim.4700110608

***Webber, M. P., Singh, A., Zeig-Owens, R., Salako, J., Skerker, M., Hall, C. B., . . . Prezant, D. J. (2021). Cancer incidence in World Trade Center-exposed and non-exposed male firefighters, as compared with the US adult male population: 2001-2016. *Occupational and environmental medicine, 78*(10), 707-714. doi:10.1136/oemed-2021-107570**

***Wende, K. E. (1996). *A study of mortality among city of Buffalo firefighters*. Graduate School of State University of New York at Buffalo.**

***Zeegers, M. P., Friesema, I. H., Goldbohm, R. A., & van den Brandt, P. A. (2004). A prospective study of occupation and prostate cancer risk. *Journal of Occupational and Environmental Medicine, 46*(3), 271-279. doi:10.1097/01.jom.0000116961.48464.6b**

***Zeig-Owens, R., Webber, M. P., Hall, C. B., Schwartz, T., Jaber, N., Weakley, J., . . . Prezant, D. J. (2011). Early assessment of cancer outcomes in New York City firefighters after the 9/11 attacks: an observational cohort study. *Lancet, 378*(9794), 898-905. doi:10.1016/S0140-6736(11)60989-6**

***Zhao, G., Erazo, B., Ronda, E., Brocal, F., & Regidor, E. (2020). Mortality Among Firefighters in Spain: 10 Years of Follow-up. *Ann Work Expo Health, 64*(6), 614-621. doi:10.1093/annweh/wxaa036**
