# Supplementary material for: Cancer risk and mortality among firefighters: a meta-analytic review
Source: Front Oncol. 2023 May 12;13:1130754. doi: 10.3389/fonc.2023.1130754 (PMC10213433; doi:10.3389/fonc.2023.1130754)
Supplement: Supplementary file 2 [file DataSheet_2.docx]

**Appendix 1**

**Fire MeSH terms**

Fire[All Fields] OR fire'[All Fields] OR fire''[All Fields] OR fire's[All Fields] OR fire1[All Fields] OR fire2[All Fields] OR fire264[All Fields] OR fire3[All Fields] OR fire717[All Fields] OR firea[All Fields] OR fireal[All Fields] OR firealarm[All Fields] OR firearig[All Fields] OR firearigt[All Fields] OR firearinger[All Fields] OR firearm[All Fields] OR firearm's[All Fields] OR firearmlegislation[All Fields] OR firearms[All Fields] OR firearms'[All Fields] OR firearmsrelated[All Fields] OR firears[All Fields] OR firearsmateriale[All Fields] OR firearsopgorelse[All Fields] OR firearsrapport[All Fields] OR firearsreglen[All Fields] OR fireaxe[All Fields] OR fireback[All Fields] OR firebag[All Fields] OR fireball[All Fields] OR fireball'[All Fields] OR fireball's[All Fields] OR fireballs[All Fields] OR firebase[All Fields] OR firebase's[All Fields] OR firebases[All Fields] OR firebases'[All Fields] OR firebaug[All Fields] OR firebaugh[All Fields] OR firebaugh's[All Fields] OR firebeetle[All Fields] OR firebell[All Fields] OR firebelly[All Fields] OR fireberry[All Fields] OR firebird[All Fields] OR firebird2[All Fields] OR firebird2trade[All Fields] OR firebirdbio[All Fields] OR fireblight[All Fields] OR firebomb[All Fields] OR firebombed[All Fields] OR firebombing[All Fields] OR firebombings[All Fields] OR firebox[All Fields] OR fireboxes[All Fields] OR firebrace[All Fields] OR firebrand[All Fields] OR firebrands[All Fields] OR firebrat[All Fields] OR firebrat's[All Fields] OR firebrats[All Fields] OR firebreak[All Fields] OR firebreak'[All Fields] OR firebreaks[All Fields] OR firebreaks'[All Fields] OR firebreather[All Fields] OR firebreathers[All Fields] OR firebreathers'[All Fields] OR firebreathing[All Fields] OR firebrick[All Fields] OR firebricks[All Fields] OR firebrigade[All Fields] OR firebright1[All Fields] OR firebrob[All Fields] OR firebrowse[All Fields] OR firebrowser[All Fields] OR firebug[All Fields] OR firebug's[All Fields] OR firebugs[All Fields] OR fireburg[All Fields] OR fireburn[All Fields] OR firebush[All Fields] OR firecenter[All Fields] OR firechief[All Fields] OR fireclay[All Fields] OR fireclays[All Fields] OR fireco[All Fields] OR fireco's[All Fields] OR firecode[All Fields] OR firecomb[All Fields] OR firecon[All Fields] OR firecount[All Fields] OR firecracker[All Fields] OR firecrackers[All Fields] OR firecrackers'[All Fields] OR firecrown[All Fields] OR firecrowns[All Fields] OR firect[All Fields] OR firectly[All Fields] OR fired[All Fields] OR fired'[All Fields] OR fired''[All Fields] OR firedagersmarsjen[All Fields] OR firedamp[All Fields] OR firedb[All Fields] OR firedebrispak[All Fields] OR firedel[All Fields] OR fireden[All Fields] OR firedepartment[All Fields] OR firedev[All Fields] OR firedlich[All Fields] OR firedman[All Fields] OR firedness[All Fields] OR firedobbelt[All Fields] OR firedoc[All Fields] OR firedock[All Fields] OR firedock's[All Fields] OR firedreich's[All Fields] OR firedrich[All Fields] OR firee[All Fields] OR fireex[All Fields] OR fireeye[All Fields] OR fireezing[All Fields] OR fireface[All Fields] OR **firefighers**[All Fields] OR **firefighers'**[All Fields] OR **firefighing**[All Fields] OR firefight[All Fields] OR **firefighter**[All Fields] OR **firefighter'**[All Fields] OR **firefighter's**[All Fields] OR firefighternearmiss[All Fields] OR firefighters[All Fields] OR **firefighters'**[All Fields] OR firefighting[All Fields] OR firefighting's[All Fields] OR firefights[All Fields] OR firefinch[All Fields] OR firefinches[All Fields] OR firefish[All Fields] OR fireflash500[All Fields] OR firefleye[All Fields] OR fireflies[All Fields] OR fireflies'[All Fields] OR firefly[All Fields] OR firefly'[All Fields] OR firefly's[All Fields] OR firefly2280[All Fields] OR fireflyfyh2008[All Fields] OR fireflytm[All Fields] OR fireflytrade[All Fields] OR fireflyxchen[All Fields] OR fireforce[All Fields] OR firefox[All Fields] OR firefoxjun[All Fields] OR firefree[All Fields] OR firefreebird[All Fields] OR firefront[All Fields] OR firegenerated[All Fields] OR firegoose[All Fields] OR fireground[All Fields] OR firegrounds[All Fields] OR fireguard[All Fields] OR fireguards[All Fields] OR fireguide[All Fields] OR firegun[All Fields] OR firehall[All Fields] OR firehammer[All Fields] OR firehawk[All Fields] OR firehawktrade[All Fields] OR fireheart[All Fields] OR fireheller[All Fields] OR firehiwot[All Fields] OR firehole[All Fields] OR firehose[All Fields] OR firehouse[All Fields] OR firehouses[All Fields] OR firehs[All Fields] OR firehttp[All Fields] OR firehun[All Fields] OR fireintolerant[All Fields] OR fireinvestigationsuk[All Fields] OR fireisi[All Fields] OR firejj217[All Fields] OR firek[All Fields] OR firek2j[All Fields] OR firekit[All Fields] OR firekite[All Fields] OR firelancet[All Fields] OR fireland[All Fields] OR firelands[All Fields] OR fireld[All Fields] OR fireleg[All Fields] OR fireless[All Fields] OR firelight[All Fields] OR firelighter[All Fields] OR firelighters[All Fields] OR fireline[All Fields] OR firelines[All Fields] OR firelit[All Fields] OR firelog[All Fields] OR firelogs[All Fields] OR firely[All Fields] OR firem[All Fields] OR firemagictrade[All Fields] OR firemail[All Fields] OR firemal[All Fields] OR **fireman**[All Fields] OR fireman**'**[All Fields] OR fireman's[All Fields] OR firemani[All Fields] OR firemanm[All Fields] OR firemann[All Fields] OR firemap[All Fields] OR firemark[All Fields] OR firemaster[All Fields] OR firemaster550[All Fields] OR firemat[All Fields] OR firemath[All Fields] OR firemed[All Fields] OR **firemen**[All Fields] OR firemen's[All Fields] OR firemine[All Fields] OR firemni[All Fields] OR firemouth[All Fields] OR firemouths[All Fields] OR firempong[All Fields] OR firen[All Fields] OR firence[All Fields] OR firend[All Fields] OR firend20942[All Fields] OR firendly[All Fields] OR firendo[All Fields] OR firendship[All Fields] OR fireness[All Fields] OR firenet[All Fields] OR firenge[All Fields] OR firengi[All Fields] OR firens[All Fields] OR firense[All Fields] OR firensia[All Fields] OR firenstein[All Fields] OR firent[All Fields] OR firenyi[All Fields] OR firenz[All Fields] OR firenza[All Fields] OR firenzani[All Fields] OR firenze[All Fields] OR firenze'[All Fields] OR firenze,[All Fields] OR firenzecenter[All Fields] OR firenzedipartimento[All Fields] OR firenzefirenze[All Fields] OR firenzeinstituto[All Fields] OR firenzuo[All Fields] OR firenzuola's[All Fields] OR firenzuoli[All Fields] OR firenzuolif[All Fields] OR fireocc[All Fields] OR fireogfyrre[All Fields] OR fireogtredive[All Fields] OR fireoved[All Fields] OR **fireperson**[All Fields] OR **firepersons**[All Fields] OR firepit[All Fields] OR firepits[All Fields] OR fireplace[All Fields] OR fireplaces[All Fields] OR fireplay[All Fields] OR fireplex[All Fields] OR fireplextrade[All Fields] OR firepol[All Fields] OR firepolished[All Fields] OR firepond[All Fields] OR firepot[All Fields] OR firepower[All Fields] OR firepro[All Fields] OR fireprone[All Fields] OR fireproneness[All Fields] OR fireproof[All Fields] OR fireproofed[All Fields] OR fireproofing[All Fields] OR fireprot[All Fields] OR fireprot's[All Fields] OR fireproxies[All Fields] OR firepsych[All Fields] OR firequency[All Fields] OR firequent[All Fields] OR firer[All Fields] OR firer's[All Fields] OR firerangers[All Fields] OR fireregulatoryvariation[All Fields] OR **firerescue**[All Fields] OR firers[All Fields] OR fires[All Fields] OR fires'[All Fields] OR firesafe[All Fields] OR firesafety[All Fields] OR firesale[All Fields] OR firescapes[All Fields] OR firescars[All Fields] OR firescope[All Fields] OR firescotland[All Fields] OR firescu[All Fields] OR firese[All Fields] OR fireseeds[All Fields] OR fireseedsnorth[All Fields] OR firesen[All Fields] OR firesert[All Fields] OR fireservice[All Fields] OR firesetter[All Fields] OR firesetters[All Fields] OR firesetters'[All Fields] OR firesetting[All Fields] OR firesheds[All Fields] OR firesheets[All Fields] OR fireside[All Fields] OR fireside'[All Fields] OR firesides[All Fields] OR firesidetox[All Fields] OR firesignal[All Fields] OR firesinger[All Fields] OR firesite[All Fields] OR firesmart[All Fields] OR firesmoke[All Fields] OR firesorb[All Fields] OR firespot[All Fields] OR firespread[All Fields] OR firest[All Fields] OR firestar[All Fields] OR firestein[All Fields] OR firestein's[All Fields] OR firestem[All Fields] OR firestem2d[All Fields] OR firester[All Fields] OR firestine[All Fields] OR fireston[All Fields] OR firestone[All Fields] OR firestone's[All Fields] OR firestonem[All Fields] OR firestorm[All Fields] OR firestorm'[All Fields] OR firestorms[All Fields] OR firestove[All Fields] OR firesum[All Fields] OR fireswept[All Fields] OR firet[All Fields] OR firetadeas[All Fields] OR firetag[All Fields] OR firetail[All Fields] OR firetailed[All Fields] OR firetails[All Fields] OR fireteanu[All Fields] OR firetec[All Fields] OR firetest[All Fields] OR firethorn[All Fields] OR firethorne[All Fields] OR firetip[All Fields] OR firetips[All Fields] OR firetj[All Fields] OR firetrains[All Fields] OR firetrap[All Fields] OR firetraps[All Fields] OR firetree[All Fields] OR firetruck[All Fields] OR firetti[All Fields] OR firetto[All Fields] OR firetube[All Fields] OR firetype[All Fields] OR fireugers[All Fields] OR fireusing[All Fields] OR firev6[All Fields] OR firevicius[All Fields] OR firevoxel[All Fields] OR firevp[All Fields] OR firew[All Fields] OR firewach[All Fields] OR firewalkers[All Fields] OR firewalkers'[All Fields] OR firewalking[All Fields] OR firewall[All Fields] OR firewall'[All Fields] OR firewall's[All Fields] OR firewalled[All Fields] OR firewalls[All Fields] OR firewalls'[All Fields] OR firewatchers[All Fields] OR firewater[All Fields] OR fireweed[All Fields] OR firewell[All Fields] OR firewire[All Fields] OR firewise[All Fields] OR firewmekbib[All Fields] OR firewoks[All Fields] OR firewood[All Fields] OR firewoods[All Fields] OR firework[All Fields] OR fireworker[All Fields] OR fireworks[All Fields] OR fireworks'[All Fields] OR fireworm[All Fields] OR fireworm's[All Fields] OR fireworms[All Fields] OR fireworms'[All Fields] OR firewtegegne[All Fields] OR firex[All Fields] OR firexon2[All Fields] OR firey[All Fields] OR firez[All Fields] OR fireze[All Fields] OR firezione[All Fields] OR firezyme[All Fields]

Fire inspector, fire inspectors, fire rescue, fire-rescue, paramedic, paramedics, firefighter, fire fighter
