# Supplementary material for: Cancer risk and mortality among firefighters: a meta-analytic review
Source: Front Oncol. 2023 May 12;13:1130754. doi: 10.3389/fonc.2023.1130754 (PMC10213433; doi:10.3389/fonc.2023.1130754)
Supplement: Supplementary file 3 [file DataSheet_3.pdf]

Codebook

Data Dictionary Codebook

03/29/2022 12:37pm

Collapse all instruments

	#	Variable / Field Name	Field Label<br>*Field Note*	Field Attributes (Field Type, Validation, Choices, Calculations, etc.)
Instrument: **Characteristics** (characteristics) Collapse				
	1	record_id	Record ID	text
	2	char_coder	Coder's name<br>(Please type your last name)<br>*please use only lowercase letters*	text
	3	characteristics_reject	Study rejected?	yesno<br>1 Yes<br>0 No
	4	reason<br>Show the field ONLY if:<br>[characteristics_reject]="1"	Why was it rejected?	notes
	5	character_si	Section Header: *Study Characteristics*<br>Study Id:<br>Last name of first author (first letter - cap) + first letter (cap) of second author's last name + first letter (cap) of third author's last name (if only two authors exist, just use Last name of first author + first letter of second author's last name)_pubyear<br>Ahn, Y.S. & Jeong, K.S. 2015 = AhnJ2015<br>Ahn, Y.S., Jeong, K.S, & Kim, K. S. 2012 = AhnJK2012<br><br>if unpublished use 0000 as year	notes, Required
	6	pub_type	Publication type	radio<br>1 Unpublished<br>2 Published<br>3 Other
	7	otherpubtype<br>Show the field ONLY if:<br>[pub_type] = '3'	Other (Please list what type...e.g. report, dissertation, government report)	notes
	8	results	Please copy and paste the abstract RESULTS section here.	notes
	9	design	Study design	radio<br>1 Longitudinal<br>2 Cross-sectional<br>3 Mixed<br>4 Case-control<br>5 Cohort<br>6 Other (Notes)<br>7 Not reported
	10	period	Period of data collection given?	yesno<br>1 Yes<br>0 No
	11	period_startday<br>Show the field ONLY if:<br>[period]='1'	Period of data collection start<br>Day:<br>*please type in DD format*	text

	12	period_startmonth<br><br>Show the field ONLY if:<br>[period]='1'	Period of data collection start<br>Month:<br>*please type in MM format*	text
	13	period_startyear<br><br>Show the field ONLY if:<br>[period]='1'	Period of data collection start<br>Year:<br>*please type in YYYY format*	text
	14	period_endday<br><br>Show the field ONLY if:<br>[period]='1'	Period of data collection end<br>Day:<br>*please type in DD format*	text
	15	period_endmonth<br><br>Show the field ONLY if:<br>[period]='1'	Period of data collection end<br>Month:<br>*please type in MM format*	text
	16	period_endyear<br><br>Show the field ONLY if:<br>[period]='1'	Period of data collection end<br>Year:<br>*please type in YYYY format*	text
	17	controlinfo<br><br>Show the field ONLY if:<br>[design] = '4'	Information on the controls	notes
	18	con_agetiming<br><br>Show the field ONLY if:<br>[design]='4'	Control group:<br>Age	checkbox<br>1 | con_agetiming___1 | At cohort start date<br>2 | con_agetiming___2 | At cohort end date<br>3 | con_agetiming___3 | At death<br>4 | con_agetiming___4 | At diagnosis<br>5 | con_agetiming___5 | At hire<br>6 | con_agetiming___6 | Not reported
	19	con_agecsm<br><br>Show the field ONLY if:<br>[con_agetiming(1)] = '1'	Control group<br>Age at cohort start date: Mean	text (number)
	20	con_agecssd<br><br>Show the field ONLY if:<br>[con_agetiming(1)] = '1'	Control group<br>Age at cohort start date : Standard Deviation<br>*If variance is given please convert to standard deviation*	text
	21	con_agecsmin<br><br>Show the field ONLY if:<br>[con_agetiming(1)] = '1'	Control group<br>Age at cohort start date: Min	text (number)
	22	con_agecsmax<br><br>Show the field ONLY if:<br>[con_agetiming(1)] = '1'	Control group<br>Age at cohort start date: Max	text
	23	con_agefreq<br><br>Show the field ONLY if:<br>[con_agetiming(1)] = '1'	Control group<br>Age at cohort start<br>Frequencies and percentages	notes
	24	con_agecem<br><br>Show the field ONLY if:<br>[con_agetiming(2)] = '1'	Control group<br>Age at cohort end date: Mean	text (number)
	25	con_agecesd<br><br>Show the field ONLY if:<br>[con_agetiming(2)] = '1'	Control group<br>Age at cohort end date : Standard Deviation<br>*If variance is given please convert to standard deviation*	text
	26	con_agecemin<br><br>Show the field ONLY if:<br>[con_agetiming(2)] = '1'	Control group<br>Age at cohort end date: Min	text (number)
	27	con_agecemax<br><br>Show the field ONLY if:<br>[con_agetiming(2)] = '1'	Control group<br>Age at cohort end date: Max	text

	28	con_ageendfreq<br><br>Show the field ONLY if:<br>[con_agetiming(2)] = '1'	Control group<br>Age at cohort end<br>Frequencies and percentages	notes
	29	con_agedeathm<br><br>Show the field ONLY if:<br>[con_agetiming(3)] = '1'	Control group<br>Age at death: Mean	text (number)
	30	con_agedeathsd<br><br>Show the field ONLY if:<br>[con_agetiming(3)] = '1'	Control group<br>Age at death : Standard Deviation<br>*If variance is given please convert to standard deviation*	text
	31	con_agedeathmin<br><br>Show the field ONLY if:<br>[con_agetiming(3)] = '1'	Control group<br>Age at death: Min	text (number)
	32	con_agedeathmax<br><br>Show the field ONLY if:<br>[con_agetiming(3)] = '1'	Control group<br>Age at death: Max	text
	33	con_agedeathfreq<br><br>Show the field ONLY if:<br>[con_agetiming(3)] = '1'	Control group<br>Age at death<br>Frequencies and percentages	notes
	34	con_agedxm<br><br>Show the field ONLY if:<br>[con_agetiming(4)] = '1'	Control group<br>Age at dx: Mean	text (number)
	35	con_agedxsd<br><br>Show the field ONLY if:<br>[con_agetiming(4)] = '1'	Control group<br>Age at dx : Standard Deviation<br>*If variance is given please convert to standard deviation*	text
	36	con_agedxmin<br><br>Show the field ONLY if:<br>[con_agetiming(4)] = '1'	Control group<br>Age at dx: Min	text (number)
	37	con_agedxmax<br><br>Show the field ONLY if:<br>[con_agetiming(4)] = '1'	Control group<br>Age at dx: Max	text
	38	con_agedxfreq<br><br>Show the field ONLY if:<br>[con_agetiming(4)] = '1'	Control group<br>Age at dx<br>Frequencies and percentages	notes
	39	con_agehirem<br><br>Show the field ONLY if:<br>[con_agetiming(5)]='1'	Control group<br>Age at hire: Mean	text
	40	con_agehiresd<br><br>Show the field ONLY if:<br>[con_agetiming(5)]='1'	Control group<br>Age at hire: SD<br>*if variance is reported please convert it to standard deviation and type here*	text
	41	con_agehiremin<br><br>Show the field ONLY if:<br>[con_agetiming(5)]='1'	Control group<br>Age at hire: Min	text
	42	con_agehiremax<br><br>Show the field ONLY if:<br>[con_agetiming(5)]='1'	Control group<br>Age at hire: Max	text
	43	con_agehirefreq<br><br>Show the field ONLY if:<br>[con_agetiming(5)] = '1'	Control group<br>Age at hire<br>Frequencies and percentages	notes
	44	con_employcont<br><br>Show the field ONLY if:<br>[design]='4'	Is duration of employment in years collected categorically or continuously?	radio<br>0 Categorically<br>1 Continuous

	45	con_employm<br><br>Show the field ONLY if:<br>[con_employcont]='1'	Control group<br>Employment in years<br>Mean:	text
	46	con_employsd<br><br>Show the field ONLY if:<br>[con_employcont]='1'	Control group<br>Employment in years<br>SD:<br>*if variance is reported please convert it to standard deviation and type here*	text
	47	con_employmin<br><br>Show the field ONLY if:<br>[con_employcont]='1'	Control group<br>Employment in years<br>Min:	text (number)
	48	con_employmax<br><br>Show the field ONLY if:<br>[con_employcont]='1'	Control group<br>Employment in years<br>Max:	text (number)
	49	con_durationcat<br><br>Show the field ONLY if:<br>[con_employcont]='0'	Type in the categories best fitting for employment in years for the control group	notes
	50	con_employstatus<br><br>Show the field ONLY if:<br>[design] = '4'	Control group<br>Employment status	radio<br>1 Part-time<br>2 Full-time<br>3 Other (Notes)<br>4 Not reported
	51	con_otheremploystatus<br><br>Show the field ONLY if:<br>[con_employstatus]='3'	Control group<br>other status	notes
	52	con_incidenttype<br><br>Show the field ONLY if:<br>[design]='4'	Control Group<br>Incident type attended	checkbox<br>1 con_incidenttype___1 All fires<br>2 con_incidenttype___2 Landscape fires<br>3 con_incidenttype___3 Vehicle fires<br>4 con_incidenttype___4 Structural<br>5 con_incidenttype___5 Other (Notes)<br>6 con_incidenttype___6 Not specified
	53	con_otherincident<br><br>Show the field ONLY if:<br>[con_incidenttype(5)]='1'	Control group<br>other incident type	notes
	54	other_studydesign<br><br>Show the field ONLY if:<br>[design] = '6'	Other (Notes)	notes
	55	eraemploy<br><br>Show the field ONLY if:<br>[design]='4'	Era of employment:<br>When they were first certified or first started working as a firefighter so that we can accurately document how long they have been exposed.	descriptive
	56	con_eratype<br><br>Show the field ONLY if:<br>[design]='4'	Control group<br>Era of first employment	radio<br>1 Continuous<br>2 Categorical
	57	con_eram<br><br>Show the field ONLY if:<br>[con_eratype]='1'	Control group<br>Era of first employment<br>Mean	text (number)
	58	con_erasd<br><br>Show the field ONLY if:<br>[con_eratype]='1'	Control group<br>Era of employment<br>SD<br>*if variance is reported please convert it to standard deviation and type here*	text (number)

<table>
<tr><td></td><td>59</td><td>con_eramin

Show the field ONLY if:
[con_eratype]='1'</td><td>Control group
Era of employment
Minimum</td><td>text (number)</td></tr>
<tr><td></td><td>60</td><td>con_eramax

Show the field ONLY if:
[con_eratype]='1'</td><td>Control group
Era of employment
Maximum</td><td>text (number)</td></tr>
<tr><td></td><td>61</td><td>con_eracat

Show the field ONLY if:
[con_eratype]='2'</td><td>Type in the categories for Control group
Era of employment</td><td>notes</td></tr>
<tr><td></td><td>62</td><td>con_gender

Show the field ONLY if:
[design]='4'</td><td>Control group
Gender</td><td>checkbox<table>
<tr><td>1</td><td>con_gender___1</td><td>Male</td></tr>
<tr><td>2</td><td>con_gender___2</td><td>Female</td></tr>
<tr><td>3</td><td>con_gender___3</td><td>Other (Please specify)</td></tr>
<tr><td>4</td><td>con_gender___4</td><td>Not reported</td></tr>
</table></td></tr>
<tr><td></td><td>63</td><td>othergender_2

Show the field ONLY if:
[con_gender(3)] = '1'</td><td>Other</td><td>notes</td></tr>
<tr><td></td><td>64</td><td>con_malen

Show the field ONLY if:
[con_gender(1)]='1'</td><td>Control group
Male n=</td><td>text (number)</td></tr>
<tr><td></td><td>65</td><td>con_malepct

Show the field ONLY if:
[con_gender(1)]='1'</td><td>Control group
Male %=</td><td>text</td></tr>
<tr><td></td><td>66</td><td>con_femalen

Show the field ONLY if:
[con_gender(2)] = '1'</td><td>Control group
Female n=</td><td>text (number)</td></tr>
<tr><td></td><td>67</td><td>con_femalepct

Show the field ONLY if:
[con_gender(2)] = '1'</td><td>Control group
Female %=</td><td>text</td></tr>
<tr><td></td><td>68</td><td>con_othergendern

Show the field ONLY if:
[con_gender(3)] = '1'</td><td>Control group
Other n=</td><td>text (number)</td></tr>
<tr><td></td><td>69</td><td>con_othergenderpct

Show the field ONLY if:
[con_gender(3)]='1'</td><td>Control group
Other %=</td><td>text (number)</td></tr>
<tr><td></td><td>70</td><td>con_race

Show the field ONLY if:
[design]='4'</td><td>Control group
Race/ Ethnicity</td><td>checkbox<table>
<tr><td>1</td><td>con_race___1</td><td>White</td></tr>
<tr><td>2</td><td>con_race___2</td><td>Black</td></tr>
<tr><td>3</td><td>con_race___3</td><td>Asian</td></tr>
<tr><td>4</td><td>con_race___4</td><td>Other</td></tr>
<tr><td>5</td><td>con_race___5</td><td>Unknown</td></tr>
<tr><td>6</td><td>con_race___6</td><td>Not reported</td></tr>
<tr><td>7</td><td>con_race___7</td><td>Notes</td></tr>
<tr><td>8</td><td>con_race___8</td><td>Hispanic</td></tr>
</table>
Custom alignment: LH</td></tr>
<tr><td></td><td>71</td><td>con_whiten

Show the field ONLY if:
[con_race(1)]='1'</td><td>Control group
White n=</td><td>text
Custom alignment: RH</td></tr>
<tr><td></td><td>72</td><td>con_whitepct

Show the field ONLY if:
[con_race(1)]='1'</td><td>Control group
White %=</td><td>text
Custom alignment: RH</td></tr>
</table>

	73	con_blackn<br><br>Show the field ONLY if:<br>[con_race(2)] = '1'	Control group<br>Black n=	text<br>Custom alignment: RH
	74	con_blackpct<br><br>Show the field ONLY if:<br>[con_race(2)] = '1'	Control group<br>Black %=	text<br>Custom alignment: RH
	75	con_asiann<br><br>Show the field ONLY if:<br>[con_race(3)] = '1'	Control group<br>Asian n=	text<br>Custom alignment: RH
	76	con_asianpct<br><br>Show the field ONLY if:<br>[con_race(3)] = '1'	Control group<br>Asian %=	text<br>Custom alignment: RH
	77	con_otherrace<br><br>Show the field ONLY if:<br>[con_race(4)] = '1'	Control group<br>Other<br>*please list the other race*	text<br>Custom alignment: RH
	78	con_othern<br><br>Show the field ONLY if:<br>[con_race(4)] = '1'	Control group<br>other n=	text (number)<br>Custom alignment: RH
	79	con_otherpct<br><br>Show the field ONLY if:<br>[con_race(4)] = '1'	Control group<br>other %=	text (number)
	80	con_unknownn<br><br>Show the field ONLY if:<br>[con_race(5)] = '1'	Control group<br>Unknown n=	text (number)<br>Custom alignment: RH
	81	con_unknownpct<br><br>Show the field ONLY if:<br>[con_race(5)]='1'	Control group<br>Unknown %=	text (number)<br>Custom alignment: RH
	82	con_hispanicn<br><br>Show the field ONLY if:<br>[con_race(8)]='1'	Control group<br>Hispanic n=	text (number)<br>Custom alignment: RH
	83	con_hispanicpct<br><br>Show the field ONLY if:<br>[con_race(8)]='1'	Control group<br>Hispanic %=	text<br>Custom alignment: RH
	84	con_notes<br><br>Show the field ONLY if:<br>[con_race(7)] = '1'	Control group race<br>Notes	notes
	85	con_smoking<br><br>Show the field ONLY if:<br>[design]='4'	Control group<br>Smoking Status	radio<br>0 Never smoker<br>1 Former smoker<br>2 Current smoker<br>3 Ever smoker<br>4 Not reported
	86	con_cigpackyears<br><br>Show the field ONLY if:<br>[con_smoking] = '1' or [con_smoking] = '2' or [con_smoking] = '3'	Control group<br>Are the cigarette pack years collected continuously or categorically?	radio<br>1 Continuously<br>2 Categorically<br>3 Not reported
	87	con_cigpackyearsm<br><br>Show the field ONLY if:<br>[con_cigpackyears] = '1'	Control group<br>Cigarette pack years<br>Mean=	text

	88	con_cigpackyearssd<br><br>Show the field ONLY if:<br>[con_cigpackyears] = '1'	Control group<br>Cigarette pack years<br>SD=<br>*if variance is reported please convert it to standard deviation and type here*	text (number)
	89	con_cigpackyearsmin<br><br>Show the field ONLY if:<br>[con_cigpackyears] = '1'	Control group<br>Cigarette pack years<br>Min=	text (number)
	90	con_cigpackyearsmax<br><br>Show the field ONLY if:<br>[con_cigpackyears] = '1'	Control group<br>Cigarette pack years<br>Max=	text
	91	con_cigpackyearscat<br><br>Show the field ONLY if:<br>[con_cigpackyears]='2'	Control group<br>Cigarette pack years	radio<br>1 | < 10 pack years<br>2 | 10-20 pack years<br>3 | 21+ pack years
	92	location	Location	checkbox<br>1 | location___1 | United States (US)<br>2 | location___2 | Non-US
	93	states<br><br>Show the field ONLY if:<br>[location(1)] = '1'	Which state(s) are included?	checkbox<br>1 | states___1 | Alabama<br>2 | states___2 | Alaska<br>3 | states___3 | Arizona<br>4 | states___4 | Arkansas<br>5 | states___5 | California<br>6 | states___6 | Colorado<br>7 | states___7 | Connecticut<br>8 | states___8 | Delaware<br>9 | states___9 | Florida<br>10 | states___10 | Georgia<br>11 | states___11 | Hawaii<br>12 | states___12 | Idaho<br>13 | states___13 | Illinois<br>14 | states___14 | Indiana<br>15 | states___15 | Iowa<br>16 | states___16 | Kansas<br>17 | states___17 | Kentucky<br>18 | states___18 | Louisiana<br>19 | states___19 | Maine<br>20 | states___20 | Maryland<br>21 | states___21 | Massachusetts<br>22 | states___22 | Michigan<br>23 | states___23 | Minnesota<br>24 | states___24 | Mississippi<br>25 | states___25 | Missouri<br>26 | states___26 | Montana<br>27 | states___27 | Nebraska<br>28 | states___28 | Nevada<br>29 | states___29 | New Hampshire<br>30 | states___30 | New Jersey<br>31 | states___31 | New Mexico

32	states___32	New York
33	states___33	North Carolina
34	states___34	North Dakota
35	states___35	Ohio
36	states___36	Oklahoma
37	states___37	Oregon
38	states___38	Pennsylvania
39	states___39	Rhode Island
40	states___40	South Carolina
41	states___41	South Dakota
42	states___42	Tennessee
43	states___43	Texas
44	states___44	Utah
45	states___45	Vermont
46	states___46	Virginia
47	states___47	Washington
48	states___48	West Virginia
49	states___49	Wisconsin
50	states___50	Wyoming

Custom alignment: LH

94	alabamacities<br><br>Show the field ONLY if:<br>[states(1)] = '1'	Which cities in Alabama?	checkbox

1	alabamacities___1	Alabaster
2	alabamacities___2	Albertville
3	alabamacities___3	Alexander City
4	alabamacities___4	Andalusia
5	alabamacities___5	Anniston
6	alabamacities___6	Arab
7	alabamacities___7	Athens
8	alabamacities___8	Atmore
9	alabamacities___9	Attalla
10	alabamacities___10	Auburn
11	alabamacities___11	Bay Minette
12	alabamacities___12	Bessemer
13	alabamacities___13	Birmingham
14	alabamacities___14	Boaz
15	alabamacities___15	Center Point
16	alabamacities___16	Chickasaw
17	alabamacities___17	Clanton
18	alabamacities___18	Cullman
19	alabamacities___19	Daphne
20	alabamacities___20	Decatur
21	alabamacities___21	Demopolis
22	alabamacities___22	Dothan
23	alabamacities___23	Enterprise
24	alabamacities___24	Eufaula
25	alabamacities___25	Fairfield
26	alabamacities___26	Fairhope
27	alabamacities___27	Florence

28	alabamacities___28	Foley
29	alabamacities___29	Forestdale
30	alabamacities___30	Fort Payne
31	alabamacities___31	Fort Rucker
32	alabamacities___32	Fultondale
33	alabamacities___33	Gadsden
34	alabamacities___34	Gardendale
35	alabamacities___35	Greenville
36	alabamacities___36	Guntersville
37	alabamacities___37	Hamilton
38	alabamacities___38	Hartselle
39	alabamacities___39	Helena
40	alabamacities___40	Homewood
41	alabamacities___41	Hoover
42	alabamacities___42	Hueytown
43	alabamacities___43	Huntsville
44	alabamacities___44	Irondale
45	alabamacities___45	Jacksonville
46	alabamacities___46	Jasper
47	alabamacities___47	Lanett
48	alabamacities___48	Leeds
49	alabamacities___49	Madison
50	alabamacities___50	Millbrook
51	alabamacities___51	Mobile
52	alabamacities___52	Monroeville
53	alabamacities___53	Montgomery
54	alabamacities___54	Moody
55	alabamacities___55	Mountain Brook
56	alabamacities___56	Muscle Shoals
57	alabamacities___57	Northport
58	alabamacities___58	Opelika
59	alabamacities___59	Opp
60	alabamacities___60	Oxford
61	alabamacities___61	Ozark
62	alabamacities___62	Pelham
63	alabamacities___63	Pell City
64	alabamacities___64	Phenix City
65	alabamacities___65	Pleasant Grove
66	alabamacities___66	Prattville
67	alabamacities___67	Prichard
68	alabamacities___68	Rainbow City
69	alabamacities___69	Roanoke
70	alabamacities___70	Russellville
71	alabamacities___71	Saks
72	alabamacities___72	Saraland
73	alabamacities___73	Scottsboro
74	alabamacities___74	Selma
75	alabamacities___75	Sheffield

				<table><tr><td>76</td><td>alabamacities___76</td><td>Smiths</td></tr><tr><td>77</td><td>alabamacities___77</td><td>Southside</td></tr><tr><td>78</td><td>alabamacities___78</td><td>Sylacauga</td></tr><tr><td>79</td><td>alabamacities___79</td><td>Talladega</td></tr><tr><td>80</td><td>alabamacities___80</td><td>Tarrant</td></tr><tr><td>81</td><td>alabamacities___81</td><td>Theodore</td></tr><tr><td>82</td><td>alabamacities___82</td><td>Tillmans Corner</td></tr><tr><td>83</td><td>alabamacities___83</td><td>Troy</td></tr><tr><td>84</td><td>alabamacities___84</td><td>Trussville</td></tr><tr><td>85</td><td>alabamacities___85</td><td>Tuscaloosa</td></tr><tr><td>86</td><td>alabamacities___86</td><td>Tuscumbia</td></tr><tr><td>87</td><td>alabamacities___87</td><td>Tuskegee</td></tr><tr><td>88</td><td>alabamacities___88</td><td>Valley</td></tr><tr><td>89</td><td>alabamacities___89</td><td>Vestavia Hills</td></tr><tr><td>90</td><td>alabamacities___90</td><td>Not listed</td></tr></table><br>Custom alignment: LH
	95	alaskacities<br>Show the field ONLY if:<br>[states(2)] = '1'	Which cities in Alaska?	checkbox<table><tr><td>1</td><td>alaskacities___1</td><td>Anchorage</td></tr><tr><td>2</td><td>alaskacities___2</td><td>Cordova</td></tr><tr><td>3</td><td>alaskacities___3</td><td>Fairbanks</td></tr><tr><td>4</td><td>alaskacities___4</td><td>Haines</td></tr><tr><td>5</td><td>alaskacities___5</td><td>Homer</td></tr><tr><td>6</td><td>alaskacities___6</td><td>Juneau</td></tr><tr><td>7</td><td>alaskacities___7</td><td>Ketchikan</td></tr><tr><td>8</td><td>alaskacities___8</td><td>Kodiak</td></tr><tr><td>9</td><td>alaskacities___9</td><td>Kotzebue</td></tr><tr><td>10</td><td>alaskacities___10</td><td>Nome</td></tr><tr><td>11</td><td>alaskacities___11</td><td>Palmer</td></tr><tr><td>12</td><td>alaskacities___12</td><td>Seward</td></tr><tr><td>13</td><td>alaskacities___13</td><td>Sitka</td></tr><tr><td>14</td><td>alaskacities___14</td><td>Skagway</td></tr><tr><td>15</td><td>alaskacities___15</td><td>Valdez</td></tr><tr><td>16</td><td>alaskacities___16</td><td>Not listed</td></tr></table><br>Custom alignment: LH

<table>
<tr>
<td></td>
<td>96</td>
<td>arizonac

Show the field ONLY if:
[states(3)] = '1'</td>
<td>Which cities in Arizona?</td>
<td>checkbox

1 | arizonac___1 | Alexander City
2 | arizonac___2 | Andalusia
3 | arizonac___3 | Anniston
4 | arizonac___4 | Athens
5 | arizonac___5 | Atmore
6 | arizonac___6 | Auburn
7 | arizonac___7 | Bessemer
8 | arizonac___8 | Birmingham
9 | arizonac___9 | Chickasaw
10 | arizonac___10 | Clanton
11 | arizonac___11 | Cullman
12 | arizonac___12 | Decatur
13 | arizonac___13 | Demopolis
14 | arizonac___14 | Dothan
15 | arizonac___15 | Enterprise
16 | arizonac___16 | Eufaula
17 | arizonac___17 | Florence
18 | arizonac___18 | Fort Payne
19 | arizonac___19 | Gadsden
20 | arizonac___20 | Greenville
21 | arizonac___21 | Guntersville
22 | arizonac___22 | Huntsville
23 | arizonac___23 | Jasper
24 | arizonac___24 | Marion
25 | arizonac___25 | Mobile
26 | arizonac___26 | Montgomery
27 | arizonac___27 | Opelika
28 | arizonac___28 | Ozark
29 | arizonac___29 | Phenix City
30 | arizonac___30 | Prichard
31 | arizonac___31 | Scottsboro
32 | arizonac___32 | Selma
33 | arizonac___33 | Sheffield
34 | arizonac___34 | Sylacauga
35 | arizonac___35 | Talladega
36 | arizonac___36 | Troy
37 | arizonac___37 | Tuscaloosa
38 | arizonac___38 | Tuscumbia
39 | arizonac___39 | Tuskegee
40 | arizonac___40 | Not listed

Custom alignment: LH</td>
</tr>
</table>

<table>
<tr><td></td><td>97</td><td>arkansasc

Show the field ONLY if:
[states(4)] = '1'</td><td>Which cities in Arkansas?</td><td>checkbox
<table>
<tr><td>1</td><td>arkansasc___1</td><td>Arkadelphia</td></tr>
<tr><td>2</td><td>arkansasc___2</td><td>Arkansas Post</td></tr>
<tr><td>3</td><td>arkansasc___3</td><td>Batesville</td></tr>
<tr><td>4</td><td>arkansasc___4</td><td>Benton</td></tr>
<tr><td>5</td><td>arkansasc___5</td><td>Blytheville</td></tr>
<tr><td>6</td><td>arkansasc___6</td><td>Camden</td></tr>
<tr><td>7</td><td>arkansasc___7</td><td>Conway</td></tr>
<tr><td>8</td><td>arkansasc___8</td><td>Crossett</td></tr>
<tr><td>9</td><td>arkansasc___9</td><td>El Dorado</td></tr>
<tr><td>10</td><td>arkansasc___10</td><td>Fayetteville</td></tr>
<tr><td>11</td><td>arkansasc___11</td><td>Forrest City</td></tr>
<tr><td>12</td><td>arkansasc___12</td><td>Fort Smith</td></tr>
<tr><td>13</td><td>arkansasc___13</td><td>Harrison</td></tr>
<tr><td>14</td><td>arkansasc___14</td><td>Helena</td></tr>
<tr><td>15</td><td>arkansasc___15</td><td>Hope</td></tr>
<tr><td>16</td><td>arkansasc___16</td><td>Hot Springs</td></tr>
<tr><td>17</td><td>arkansasc___17</td><td>Jacksonville</td></tr>
<tr><td>18</td><td>arkansasc___18</td><td>Jonesboro</td></tr>
<tr><td>19</td><td>arkansasc___19</td><td>Little Rock</td></tr>
<tr><td>20</td><td>arkansasc___20</td><td>Magnolia</td></tr>
<tr><td>21</td><td>arkansasc___21</td><td>Morrilton</td></tr>
<tr><td>22</td><td>arkansasc___22</td><td>Newport</td></tr>
<tr><td>23</td><td>arkansasc___23</td><td>North Little Rock</td></tr>
<tr><td>24</td><td>arkansasc___24</td><td>Osceola</td></tr>
<tr><td>25</td><td>arkansasc___25</td><td>Pine Bluff</td></tr>
<tr><td>26</td><td>arkansasc___26</td><td>Rogers</td></tr>
<tr><td>27</td><td>arkansasc___27</td><td>Searcy</td></tr>
<tr><td>28</td><td>arkansasc___28</td><td>Stuttgart</td></tr>
<tr><td>29</td><td>arkansasc___29</td><td>Van Buren</td></tr>
<tr><td>30</td><td>arkansasc___30</td><td>West Memphis</td></tr>
<tr><td>31</td><td>arkansasc___31</td><td>Not listed</td></tr>
</table>
Custom alignment: LH</td></tr>
<tr><td></td><td>98</td><td>californiac

Show the field ONLY if:
[states(5)] = '1'</td><td>Which cities in California?</td><td>checkbox
<table>
<tr><td>1</td><td>californiac___1</td><td>Alameda</td></tr>
<tr><td>2</td><td>californiac___2</td><td>Alhambra</td></tr>
<tr><td>3</td><td>californiac___3</td><td>Anaheim</td></tr>
<tr><td>4</td><td>californiac___4</td><td>Antioch</td></tr>
<tr><td>5</td><td>californiac___5</td><td>Arcadia</td></tr>
<tr><td>6</td><td>californiac___6</td><td>Bakersfield</td></tr>
<tr><td>7</td><td>californiac___7</td><td>Barstow</td></tr>
<tr><td>8</td><td>californiac___8</td><td>Belmont</td></tr>
<tr><td>9</td><td>californiac___9</td><td>Berkeley</td></tr>
<tr><td>10</td><td>californiac___10</td><td>Beverly Hills</td></tr>
<tr><td>11</td><td>californiac___11</td><td>Brea</td></tr>
<tr><td>12</td><td>californiac___12</td><td>Buena Park</td></tr>
<tr><td>13</td><td>californiac___13</td><td>Burbank</td></tr>
<tr><td>14</td><td>californiac___14</td><td>Calexico</td></tr>
</table></td></tr>
</table>

15	californiac___15	Calistoga
16	californiac___16	Carlsbad
17	californiac___17	Carmel
18	californiac___18	Chico
19	californiac___19	Chula Vista
20	californiac___20	Claremont
21	californiac___21	Compton
22	californiac___22	Concord
23	californiac___23	Corona
24	californiac___24	Coronado
25	californiac___25	Costa Mesa
26	californiac___26	Culver City
27	californiac___27	Daly City
28	californiac___28	Davis
29	californiac___29	Downey
30	californiac___30	El Centro
31	californiac___31	El Cerrito
32	californiac___32	El Monte
33	californiac___33	Escondido
34	californiac___34	Eureka
35	californiac___35	Fairfield
36	californiac___36	Fontana
37	californiac___37	Fremont
38	californiac___38	Fresno
39	californiac___39	Fullerton
40	californiac___40	Garden Grove
41	californiac___41	Glendale
42	californiac___42	Hayward
43	californiac___43	Hollywood
44	californiac___44	Huntington Beach
45	californiac___45	Indio
46	californiac___46	Inglewood
47	californiac___47	Irvine
48	californiac___48	La Habra
49	californiac___49	Laguna Beach
50	californiac___50	Lancaster
51	californiac___51	Livermore
52	californiac___52	Lodi
53	californiac___53	Lompoc
54	californiac___54	Long Beach
55	californiac___55	Los Angeles
56	californiac___56	Malibu
57	californiac___57	Martinez
58	californiac___58	Marysville
59	californiac___59	Menlo Park
60	californiac___60	Merced
61	californiac___61	Modesto
62	californiac___62	Monterey

63	californiac___63	Mountain View
64	californiac___64	Napa
65	californiac___65	Needles
66	californiac___66	Newport Beach
67	californiac___67	Norwalk
68	californiac___68	Novato
69	californiac___69	Oakland
70	californiac___70	Oceanside
71	californiac___71	Ojai
72	californiac___72	Ontario
73	californiac___73	Orange
74	californiac___74	Oroville
75	californiac___75	Oxnard
76	californiac___76	Pacific Grove
77	californiac___77	Palm Springs
78	californiac___78	Palmdale
79	californiac___79	Palo Alto
80	californiac___80	Pasadena
81	californiac___81	Petaluma
82	californiac___82	Pomona
83	californiac___83	Port Hueneme
84	californiac___84	Rancho Cucamonga
85	californiac___85	Red Bluff
86	californiac___86	Redding
87	californiac___87	Redlands
88	californiac___88	Redondo Beach
89	californiac___89	Redwood City
90	californiac___90	Richmond
91	californiac___91	Riverside
92	californiac___92	Roseville
93	californiac___93	Sacramento
94	californiac___94	Salinas
95	californiac___95	San Bernardino
96	californiac___96	San Clemente
97	californiac___97	San Diego
98	californiac___98	San Fernando
99	californiac___99	San Francisco
100	californiac___100	San Gabriel
101	californiac___101	San Jose
102	californiac___102	San Juan Capistrano
103	californiac___103	San Leandro
104	californiac___104	San Luis Obispo
105	californiac___105	San Marino
106	californiac___106	San Mateo
107	californiac___107	San Pedro
108	californiac___108	San Rafael
109	californiac___109	San Simeon
110	californiac___110	Santa Ana

111	californiac___111	Santa Barbara
112	californiac___112	Santa Clara
113	californiac___113	Santa Clarita
114	californiac___114	Santa Cruz
115	californiac___115	Santa Monica
116	californiac___116	Santa Rosa
117	californiac___117	Sausalito
118	californiac___118	Simi Valley
119	californiac___119	Sonoma
120	californiac___120	South San Francisco
121	californiac___121	Stockton
122	californiac___122	Sunnyvale
123	californiac___123	Susanville
124	californiac___124	Thousand Oaks
125	californiac___125	Torrance
126	californiac___126	Turlock
127	californiac___127	Ukiah
128	californiac___128	Vallejo
129	californiac___129	Ventura
130	californiac___130	Victorville
131	californiac___131	Visalia
132	californiac___132	Walnut Creek
133	californiac___133	Watts
134	californiac___134	West Covina
135	californiac___135	Whittier
136	californiac___136	Woodland
137	californiac___137	Yorba Linda
138	californiac___138	Yuba City
139	californiac___139	Not listed

Custom alignment: LH

	99	coloradoc<br><br>Show the field ONLY if:<br>[states(6)] = '1'	Which cities in Colorado?	checkbox<br>1 coloradoc___1 Alamosa<br>2 coloradoc___2 Aspen<br>3 coloradoc___3 Aurora<br>4 coloradoc___4 Boulder<br>5 coloradoc___5 Breckenridge<br>6 coloradoc___6 Brighton<br>7 coloradoc___7 Canon City<br>8 coloradoc___8 Central City<br>9 coloradoc___9 Climax<br>10 coloradoc___10 Colorado Springs<br>11 coloradoc___11 Cortez<br>12 coloradoc___12 Cripple Creek<br>13 coloradoc___13 Denver<br>14 coloradoc___14 Durango<br>15 coloradoc___15 Englewood<br>16 coloradoc___16 Estes Park<br>17 coloradoc___17 Fort Collins<br>18 coloradoc___18 Fort Morgan<br>19 coloradoc___19 Georgetown<br>20 coloradoc___20 Glenwood Springs<br>21 coloradoc___21 Golden<br>22 coloradoc___22 Grand Junction<br>23 coloradoc___23 Greeley<br>24 coloradoc___24 Gunnison<br>25 coloradoc___25 La Junta<br>26 coloradoc___26 Leadville<br>27 coloradoc___27 Littleton<br>28 coloradoc___28 Longmont<br>29 coloradoc___29 Loveland<br>30 coloradoc___30 Montrose<br>31 coloradoc___31 Ouray<br>32 coloradoc___32 Pagosa Springs<br>33 coloradoc___33 Pueblo<br>34 coloradoc___34 Silverton<br>35 coloradoc___35 Steamboat Springs<br>36 coloradoc___36 Sterling<br>37 coloradoc___37 Telluride<br>38 coloradoc___38 Trinidad<br>39 coloradoc___39 Vail<br>40 coloradoc___40 Walsenburg<br>41 coloradoc___41 Westminster<br>42 coloradoc___42 Not listed<br><br>Custom alignment: LH
	100	connecticutc<br><br>Show the field ONLY if:<br>[states(7)] = '1'	Which cities in Connecticut?	checkbox<br>1 connecticutc___1 Ansonia<br>2 connecticutc___2 Berlin<br>3 connecticutc___3 Bloomfield

4	connecticutc___4	Branford
5	connecticutc___5	Bridgeport
6	connecticutc___6	Bristol
7	connecticutc___7	Coventry
8	connecticutc___8	Danbury
9	connecticutc___9	Darien
10	connecticutc___10	Derby
11	connecticutc___11	East Hartford
12	connecticutc___12	East Haven
13	connecticutc___13	Enfield
14	connecticutc___14	Fairfield
15	connecticutc___15	Farmington
16	connecticutc___16	Greenwich
17	connecticutc___17	Groton
18	connecticutc___18	Guilford
19	connecticutc___19	Hamden
20	connecticutc___20	Hartford
21	connecticutc___21	Lebanon
22	connecticutc___22	Litchfield
23	connecticutc___23	Manchester
24	connecticutc___24	Mansfield
25	connecticutc___25	Meriden
26	connecticutc___26	Middletown
27	connecticutc___27	Milford
28	connecticutc___28	Mystic
29	connecticutc___29	Naugatuck
30	connecticutc___30	New Britain
31	connecticutc___31	New Haven
32	connecticutc___32	New London
33	connecticutc___33	North Haven
34	connecticutc___34	Norwalk
35	connecticutc___35	Norwich
36	connecticutc___36	Old Saybrook
37	connecticutc___37	Orange
38	connecticutc___38	Seymour
39	connecticutc___39	Shelton
40	connecticutc___40	Simsbury
41	connecticutc___41	Southington
42	connecticutc___42	Stamford
43	connecticutc___43	Stonington
44	connecticutc___44	Stratford
45	connecticutc___45	Torrington
46	connecticutc___46	Wallingford
47	connecticutc___47	Waterbury
48	connecticutc___48	Waterford
49	connecticutc___49	Watertown
50	connecticutc___50	West Hartford
51	connecticutc___51	West Haven

				52 | connecticutc___52 | Westport<br>53 | connecticutc___53 | Wethersfield<br>54 | connecticutc___54 | Willimantic<br>55 | connecticutc___55 | Windham<br>56 | connecticutc___56 | Windsor<br>57 | connecticutc___57 | Windsor Locks<br>58 | connecticutc___58 | Winsted<br>59 | connecticutc___59 | Not listed<br><br>Custom alignment: LH
	101	delawarec<br><br>Show the field ONLY if:<br>[states(8)] = '1'	Which cities in Delaware?	checkbox<br>1 | delawarec___1 | Dover<br>2 | delawarec___2 | Lewes<br>3 | delawarec___3 | Milford<br>4 | delawarec___4 | New Castle<br>5 | delawarec___5 | Newark<br>6 | delawarec___6 | Smyrna<br>7 | delawarec___7 | Wilmington<br>8 | delawarec___8 | Not listed<br><br>Custom alignment: LH
	102	floridac<br><br>Show the field ONLY if:<br>[states(9)] = '1'	Which cities in Florida?	checkbox<br>1 | floridac___1 | Apalachicola<br>2 | floridac___2 | Bartow<br>3 | floridac___3 | Belle Glade<br>4 | floridac___4 | Boca Raton<br>5 | floridac___5 | Bradenton<br>6 | floridac___6 | Cape Coral<br>7 | floridac___7 | Clearwater<br>8 | floridac___8 | Cocoa Beach<br>9 | floridac___9 | Cocoa-Rockledge<br>10 | floridac___10 | Coral Gables<br>11 | floridac___11 | Daytona Beach<br>12 | floridac___12 | De Land<br>13 | floridac___13 | Deerfield Beach<br>14 | floridac___14 | Delray Beach<br>15 | floridac___15 | Fernandina Beach<br>16 | floridac___16 | Fort Lauderdale<br>17 | floridac___17 | Fort Myers<br>18 | floridac___18 | Fort Pierce<br>19 | floridac___19 | Fort Walton Beach<br>20 | floridac___20 | Gainesville<br>21 | floridac___21 | Hallandale Beach<br>22 | floridac___22 | Hialeah<br>23 | floridac___23 | Hollywood<br>24 | floridac___24 | Homestead<br>25 | floridac___25 | Jacksonville<br>26 | floridac___26 | Key West<br>27 | floridac___27 | Lake City

28	floridac___28	Lake Wales
29	floridac___29	Lakeland
30	floridac___30	Largo
31	floridac___31	Melbourne
32	floridac___32	Miami
33	floridac___33	Miami Beach
34	floridac___34	Naples
35	floridac___35	New Smyrna Beach
36	floridac___36	Ocala
37	floridac___37	Orlando
38	floridac___38	Ormond Beach
39	floridac___39	Palatka
40	floridac___40	Palm Bay
41	floridac___41	Palm Beach
42	floridac___42	Panama City
43	floridac___43	Pensacola
44	floridac___44	Pompano Beach
45	floridac___45	Saint Augustine
46	floridac___46	Saint Petersburg
47	floridac___47	Sanford
48	floridac___48	Sarasota
49	floridac___49	Sebring
50	floridac___50	Tallahassee
51	floridac___51	Tampa
52	floridac___52	Tarpon Springs
53	floridac___53	Titusville
54	floridac___54	Venice
55	floridac___55	West Palm Beach
56	floridac___56	White Springs
57	floridac___57	Winter Haven
58	floridac___58	Winter Park
59	floridac___59	Not listed

Custom alignment: LH

<table>
<tr>
<td></td>
<td>103</td>
<td>georgiac

Show the field ONLY if:
[states(10)] = '1'</td>
<td>Which cities in Georgia?</td>
<td>checkbox
<table>
<tr><td>1</td><td>georgiac___1</td><td>Albany</td></tr>
<tr><td>2</td><td>georgiac___2</td><td>Americus</td></tr>
<tr><td>3</td><td>georgiac___3</td><td>Andersonville</td></tr>
<tr><td>4</td><td>georgiac___4</td><td>Athens</td></tr>
<tr><td>5</td><td>georgiac___5</td><td>Atlanta</td></tr>
<tr><td>6</td><td>georgiac___6</td><td>Augusta</td></tr>
<tr><td>7</td><td>georgiac___7</td><td>Bainbridge</td></tr>
<tr><td>8</td><td>georgiac___8</td><td>Blairsville</td></tr>
<tr><td>9</td><td>georgiac___9</td><td>Brunswick</td></tr>
<tr><td>10</td><td>georgiac___10</td><td>Calhoun</td></tr>
<tr><td>11</td><td>georgiac___11</td><td>Carrollton</td></tr>
<tr><td>12</td><td>georgiac___12</td><td>Columbus</td></tr>
<tr><td>13</td><td>georgiac___13</td><td>Dahlonega</td></tr>
<tr><td>14</td><td>georgiac___14</td><td>Dalton</td></tr>
<tr><td>15</td><td>georgiac___15</td><td>Darien</td></tr>
<tr><td>16</td><td>georgiac___16</td><td>Decatur</td></tr>
<tr><td>17</td><td>georgiac___17</td><td>Douglas</td></tr>
<tr><td>18</td><td>georgiac___18</td><td>East Point</td></tr>
<tr><td>19</td><td>georgiac___19</td><td>Fitzgerald</td></tr>
<tr><td>20</td><td>georgiac___20</td><td>Fort Valley</td></tr>
<tr><td>21</td><td>georgiac___21</td><td>Gainesville</td></tr>
<tr><td>22</td><td>georgiac___22</td><td>La Grange</td></tr>
<tr><td>23</td><td>georgiac___23</td><td>Macon</td></tr>
<tr><td>24</td><td>georgiac___24</td><td>Marietta</td></tr>
<tr><td>25</td><td>georgiac___25</td><td>Milledgeville</td></tr>
<tr><td>26</td><td>georgiac___26</td><td>Plains</td></tr>
<tr><td>27</td><td>georgiac___27</td><td>Rome</td></tr>
<tr><td>28</td><td>georgiac___28</td><td>Savannah</td></tr>
<tr><td>29</td><td>georgiac___29</td><td>Toccoa</td></tr>
<tr><td>30</td><td>georgiac___30</td><td>Valdosta</td></tr>
<tr><td>31</td><td>georgiac___31</td><td>Warm Springs</td></tr>
<tr><td>32</td><td>georgiac___32</td><td>Warner Robins</td></tr>
<tr><td>33</td><td>georgiac___33</td><td>Washington</td></tr>
<tr><td>34</td><td>georgiac___34</td><td>Waycross</td></tr>
<tr><td>35</td><td>georgiac___35</td><td>Not listed</td></tr>
</table>
Custom alignment: LH</td>
</tr>
</table>

<table>
<tr><td></td><td>104</td><td>hawaiic

Show the field ONLY if:
[states(11)] = '1'</td><td>Which cities in Hawaii?</td><td>checkbox
<table><tr><td>1</td><td>hawaiic___1</td><td>Hanalei</td></tr><tr><td>2</td><td>hawaiic___2</td><td>Hilo</td></tr><tr><td>3</td><td>hawaiic___3</td><td>Honaunau</td></tr><tr><td>4</td><td>hawaiic___4</td><td>Honolulu</td></tr><tr><td>5</td><td>hawaiic___5</td><td>Kahului</td></tr><tr><td>6</td><td>hawaiic___6</td><td>Kaneohe</td></tr><tr><td>7</td><td>hawaiic___7</td><td>Kapaa</td></tr><tr><td>8</td><td>hawaiic___8</td><td>Kawaihae</td></tr><tr><td>9</td><td>hawaiic___9</td><td>Lahaina</td></tr><tr><td>10</td><td>hawaiic___10</td><td>Laie</td></tr><tr><td>11</td><td>hawaiic___11</td><td>Wahiawa</td></tr><tr><td>12</td><td>hawaiic___12</td><td>Wailuku</td></tr><tr><td>13</td><td>hawaiic___13</td><td>Waimea</td></tr><tr><td>14</td><td>hawaiic___14</td><td>Not listed</td></tr></table>
Custom alignment: LH</td></tr>
<tr><td></td><td>105</td><td>idaho

Show the field ONLY if:
[states(12)] = '1'</td><td>Which cities in Idaho?</td><td>checkbox
<table><tr><td>1</td><td>idaho___1</td><td>Blackfoot</td></tr><tr><td>2</td><td>idaho___2</td><td>Boise</td></tr><tr><td>3</td><td>idaho___3</td><td>Bonners Ferry</td></tr><tr><td>4</td><td>idaho___4</td><td>Caldwell</td></tr><tr><td>5</td><td>idaho___5</td><td>Coeur d'Alene</td></tr><tr><td>6</td><td>idaho___6</td><td>Idaho City</td></tr><tr><td>7</td><td>idaho___7</td><td>Idaho Falls</td></tr><tr><td>8</td><td>idaho___8</td><td>Kellogg</td></tr><tr><td>9</td><td>idaho___9</td><td>Lewiston</td></tr><tr><td>10</td><td>idaho___10</td><td>Moscow</td></tr><tr><td>11</td><td>idaho___11</td><td>Nampa</td></tr><tr><td>12</td><td>idaho___12</td><td>Pocatello</td></tr><tr><td>13</td><td>idaho___13</td><td>Priest River</td></tr><tr><td>14</td><td>idaho___14</td><td>Rexburg</td></tr><tr><td>15</td><td>idaho___15</td><td>Sun Valley</td></tr><tr><td>16</td><td>idaho___16</td><td>Twin Falls</td></tr><tr><td>17</td><td>idaho___17</td><td>Not listed</td></tr></table>
Custom alignment: LH</td></tr>
<tr><td></td><td>106</td><td>illinoisc

Show the field ONLY if:
[states(13)] = '1'</td><td>Which cities in Illinois?</td><td>checkbox
<table><tr><td>1</td><td>illinoisc___1</td><td>Alton</td></tr><tr><td>2</td><td>illinoisc___2</td><td>Arlington Heights</td></tr><tr><td>3</td><td>illinoisc___3</td><td>Arthur</td></tr><tr><td>4</td><td>illinoisc___4</td><td>Aurora</td></tr><tr><td>5</td><td>illinoisc___5</td><td>Belleville</td></tr><tr><td>6</td><td>illinoisc___6</td><td>Belvidere</td></tr><tr><td>7</td><td>illinoisc___7</td><td>Bloomington</td></tr><tr><td>8</td><td>illinoisc___8</td><td>Brookfield</td></tr><tr><td>9</td><td>illinoisc___9</td><td>Cahokia</td></tr><tr><td>10</td><td>illinoisc___10</td><td>Cairo</td></tr><tr><td>11</td><td>illinoisc___11</td><td>Calumet City</td></tr><tr><td>12</td><td>illinoisc___12</td><td>Canton</td></tr></table></td></tr>
</table>

13	illinoisc___13	Carbondale
14	illinoisc___14	Carlinville
15	illinoisc___15	Carthage
16	illinoisc___16	Centralia
17	illinoisc___17	Champaign
18	illinoisc___18	Charleston
19	illinoisc___19	Chester
20	illinoisc___20	Chicago
21	illinoisc___21	Chicago Heights
22	illinoisc___22	Cicero
23	illinoisc___23	Collinsville
24	illinoisc___24	Danville
25	illinoisc___25	Decatur
26	illinoisc___26	DeKalb
27	illinoisc___27	Des Plaines
28	illinoisc___28	Dixon
29	illinoisc___29	East Moline
30	illinoisc___30	East Saint Louis
31	illinoisc___31	Effingham
32	illinoisc___32	Elgin
33	illinoisc___33	Elmhurst
34	illinoisc___34	Evanston
35	illinoisc___35	Freeport
36	illinoisc___36	Galena
37	illinoisc___37	Galesburg
38	illinoisc___38	Glen Ellyn
39	illinoisc___39	Glenview
40	illinoisc___40	Granite City
41	illinoisc___41	Harrisburg
42	illinoisc___42	Herrin
43	illinoisc___43	Highland Park
44	illinoisc___44	Jacksonville
45	illinoisc___45	Joliet
46	illinoisc___46	Kankakee
47	illinoisc___47	Kaskaskia
48	illinoisc___48	Kewanee
49	illinoisc___49	La Salle
50	illinoisc___50	Lake Forest
51	illinoisc___51	Libertyville
52	illinoisc___52	Lincoln
53	illinoisc___53	Lisle
54	illinoisc___54	Lombard
55	illinoisc___55	Macomb
56	illinoisc___56	Mattoon
57	illinoisc___57	Moline
58	illinoisc___58	Monmouth
59	illinoisc___59	Mount Vernon
60	illinoisc___60	Mundelein

61	illinoisc___61	Naperville
62	illinoisc___62	Nauvoo
63	illinoisc___63	Normal
64	illinoisc___64	North Chicago
65	illinoisc___65	Oak Park
66	illinoisc___66	Oregon
67	illinoisc___67	Ottawa
68	illinoisc___68	Palatine
69	illinoisc___69	Park Forest
70	illinoisc___70	Park Ridge
71	illinoisc___71	Pekin
72	illinoisc___72	Peoria
73	illinoisc___73	Petersburg
74	illinoisc___74	Pontiac
75	illinoisc___75	Quincy
76	illinoisc___76	Rantoul
77	illinoisc___77	River Forest
78	illinoisc___78	Rock Island
79	illinoisc___79	Rockford
80	illinoisc___80	Salem
81	illinoisc___81	Shawneetown
82	illinoisc___82	Skokie
83	illinoisc___83	South Holland
84	illinoisc___84	Springfield
85	illinoisc___85	Streator
86	illinoisc___86	Summit
87	illinoisc___87	Urbana
88	illinoisc___88	Vandalia
89	illinoisc___89	Virden
90	illinoisc___90	Waukegan
91	illinoisc___91	Wheaton
92	illinoisc___92	Wilmette
93	illinoisc___93	Winnetka
94	illinoisc___94	Wood River
95	illinoisc___95	Zion
96	illinoisc___96	Not listed

Custom alignment: LH

<table>
<tr>
<td></td>
<td>107</td>
<td>indianac

Show the field ONLY if:
[states(14)] = '1'</td>
<td>Which cities in Indiana</td>
<td>checkbox
<table>
<tr><td>1</td><td>indianac___1</td><td>Anderson</td></tr>
<tr><td>2</td><td>indianac___2</td><td>Bedford</td></tr>
<tr><td>3</td><td>indianac___3</td><td>Bloomington</td></tr>
<tr><td>4</td><td>indianac___4</td><td>Columbus</td></tr>
<tr><td>5</td><td>indianac___5</td><td>Connersville</td></tr>
<tr><td>6</td><td>indianac___6</td><td>Corydon</td></tr>
<tr><td>7</td><td>indianac___7</td><td>Crawfordsville</td></tr>
<tr><td>8</td><td>indianac___8</td><td>East Chicago</td></tr>
<tr><td>9</td><td>indianac___9</td><td>Elkhart</td></tr>
<tr><td>10</td><td>indianac___10</td><td>Elwood</td></tr>
<tr><td>11</td><td>indianac___11</td><td>Evansville</td></tr>
<tr><td>12</td><td>indianac___12</td><td>Fort Wayne</td></tr>
<tr><td>13</td><td>indianac___13</td><td>French Lick</td></tr>
<tr><td>14</td><td>indianac___14</td><td>Gary</td></tr>
<tr><td>15</td><td>indianac___15</td><td>Geneva</td></tr>
<tr><td>16</td><td>indianac___16</td><td>Goshen</td></tr>
<tr><td>17</td><td>indianac___17</td><td>Greenfield</td></tr>
<tr><td>18</td><td>indianac___18</td><td>Hammond</td></tr>
<tr><td>19</td><td>indianac___19</td><td>Hobart</td></tr>
<tr><td>20</td><td>indianac___20</td><td>Huntington</td></tr>
<tr><td>21</td><td>indianac___21</td><td>Indianapolis</td></tr>
<tr><td>22</td><td>indianac___22</td><td>Jeffersonville</td></tr>
<tr><td>23</td><td>indianac___23</td><td>Kokomo</td></tr>
<tr><td>24</td><td>indianac___24</td><td>Lafayette</td></tr>
<tr><td>25</td><td>indianac___25</td><td>Madison</td></tr>
<tr><td>26</td><td>indianac___26</td><td>Marion</td></tr>
<tr><td>27</td><td>indianac___27</td><td>Michigan City</td></tr>
<tr><td>28</td><td>indianac___28</td><td>Mishawaka</td></tr>
<tr><td>29</td><td>indianac___29</td><td>Muncie</td></tr>
<tr><td>30</td><td>indianac___30</td><td>Nappanee</td></tr>
<tr><td>31</td><td>indianac___31</td><td>Nashville</td></tr>
<tr><td>32</td><td>indianac___32</td><td>New Albany</td></tr>
<tr><td>33</td><td>indianac___33</td><td>New Castle</td></tr>
<tr><td>34</td><td>indianac___34</td><td>New Harmony</td></tr>
<tr><td>35</td><td>indianac___35</td><td>Peru</td></tr>
<tr><td>36</td><td>indianac___36</td><td>Plymouth</td></tr>
<tr><td>37</td><td>indianac___37</td><td>Richmond</td></tr>
<tr><td>38</td><td>indianac___38</td><td>Santa Claus</td></tr>
<tr><td>39</td><td>indianac___39</td><td>Shelbyville</td></tr>
<tr><td>40</td><td>indianac___40</td><td>South Bend</td></tr>
<tr><td>41</td><td>indianac___41</td><td>Terre Haute</td></tr>
<tr><td>42</td><td>indianac___42</td><td>Valparaiso</td></tr>
<tr><td>43</td><td>indianac___43</td><td>Vincennes</td></tr>
<tr><td>44</td><td>indianac___44</td><td>Wabash</td></tr>
<tr><td>45</td><td>indianac___45</td><td>West Lafayette</td></tr>
<tr><td>46</td><td>indianac___46</td><td>Not listed</td></tr>
</table>

Custom alignment: LH</td>
</tr>
</table>

	108	iowac<br><br>Show the field ONLY if:<br>[states(15)] = '1'	Which cities in Iowa?	checkbox<br>1 | iowac___1 | Amana Colonies<br>2 | iowac___2 | Ames<br>3 | iowac___3 | Boone<br>4 | iowac___4 | Burlington<br>5 | iowac___5 | Cedar Falls<br>6 | iowac___6 | Cedar Rapids<br>7 | iowac___7 | Charles City<br>8 | iowac___8 | Cherokee<br>9 | iowac___9 | Clinton<br>10 | iowac___10 | Council Bluffs<br>11 | iowac___11 | Davenport<br>12 | iowac___12 | Des Moines<br>13 | iowac___13 | Dubuque<br>14 | iowac___14 | Estherville<br>15 | iowac___15 | Fairfield<br>16 | iowac___16 | Fort Dodge<br>17 | iowac___17 | Grinnell<br>18 | iowac___18 | Indianola<br>19 | iowac___19 | Iowa City<br>20 | iowac___20 | Keokuk<br>21 | iowac___21 | Mason City<br>22 | iowac___22 | Mount Pleasant<br>23 | iowac___23 | Muscatine<br>24 | iowac___24 | Newton<br>25 | iowac___25 | Oskaloosa<br>26 | iowac___26 | Ottumwa<br>27 | iowac___27 | Sioux City<br>28 | iowac___28 | Waterloo<br>29 | iowac___29 | Webster City<br>30 | iowac___30 | West Des Moines<br>31 | iowac___31 | Not listed<br><br>Custom alignment: LH

	109	kansasc<br><br>Show the field ONLY if:<br>[states(16)] = '1'	Which cities in Kansas?	checkbox<br>1 kansasc___1 Abilene<br>2 kansasc___2 Arkansas City<br>3 kansasc___3 Atchison<br>4 kansasc___4 Chanute<br>5 kansasc___5 Coffeyville<br>6 kansasc___6 Council Grove<br>7 kansasc___7 Dodge City<br>8 kansasc___8 Emporia<br>9 kansasc___9 Fort Scott<br>10 kansasc___10 Garden City<br>11 kansasc___11 Great Bend<br>12 kansasc___12 Hays<br>13 kansasc___13 Hutchinson<br>14 kansasc___14 Independence<br>15 kansasc___15 Junction City<br>16 kansasc___16 Kansas City<br>17 kansasc___17 Lawrence<br>18 kansasc___18 Leavenworth<br>19 kansasc___19 Liberal<br>20 kansasc___20 Manhattan<br>21 kansasc___21 McPherson<br>22 kansasc___22 Medicine Lodge<br>23 kansasc___23 Newton<br>24 kansasc___24 Olathe<br>25 kansasc___25 Osawatomie<br>26 kansasc___26 Ottawa<br>27 kansasc___27 Overland Park<br>28 kansasc___28 Pittsburg<br>29 kansasc___29 Salina<br>30 kansasc___30 Shawnee<br>31 kansasc___31 Smith Center<br>32 kansasc___32 Topeka<br>33 kansasc___33 Wichita<br>34 kansasc___34 Not listed<br><br>Custom alignment: LH

	110	kentuckyc<br><br>Show the field ONLY if:<br>[states(17)] = '1'	Which cities in Kentucky?	checkbox<br>1 kentuckyc___1 Ashland<br>2 kentuckyc___2 Barbourville<br>3 kentuckyc___3 Bardstown<br>4 kentuckyc___4 Berea<br>5 kentuckyc___5 Boonesborough<br>6 kentuckyc___6 Bowling Green<br>7 kentuckyc___7 Campbellsville<br>8 kentuckyc___8 Covington<br>9 kentuckyc___9 Danville<br>10 kentuckyc___10 Elizabethtown<br>11 kentuckyc___11 Frankfort<br>12 kentuckyc___12 Harlan<br>13 kentuckyc___13 Harrodsburg<br>14 kentuckyc___14 Hazard<br>15 kentuckyc___15 Henderson<br>16 kentuckyc___16 Hodgenville<br>17 kentuckyc___17 Hopkinsville<br>18 kentuckyc___18 Lexington<br>19 kentuckyc___19 Louisville<br>20 kentuckyc___20 Mayfield<br>21 kentuckyc___21 Maysville<br>22 kentuckyc___22 Middlesboro<br>23 kentuckyc___23 Newport<br>24 kentuckyc___24 Owensboro<br>25 kentuckyc___25 Paducah<br>26 kentuckyc___26 Paris<br>27 kentuckyc___27 Richmond<br>28 kentuckyc___28 Not listed<br><br>Custom alignment: LH

	111	louisianac<br><br>Show the field ONLY if:<br>[states(18)] = '1'	Which cities in Louisiana?	checkbox

1	louisianac___1	Abbeville
2	louisianac___2	Alexandria
3	louisianac___3	Bastrop
4	louisianac___4	Baton Rouge
5	louisianac___5	Bogalusa
6	louisianac___6	Bossier City
7	louisianac___7	Gretna
8	louisianac___8	Houma
9	louisianac___9	Lafayette
10	louisianac___10	Lake Charles
11	louisianac___11	Monroe
12	louisianac___12	Morgan City
13	louisianac___13	Natchitoches
14	louisianac___14	New Iberia
15	louisianac___15	New Orleans
16	louisianac___16	Opelousas
17	louisianac___17	Ruston
18	louisianac___18	Saint Martinville
19	louisianac___19	Shreveport
20	louisianac___20	Thibodaux
21	louisianac___21	Not listed

Custom alignment: LH

<table>
<tr><td></td><td>112</td><td>mainec

Show the field ONLY if:
[states(19)] = '1'</td><td>Which cities in Maine?</td><td>checkbox
<table>
<tr><td>1</td><td>mainec___1</td><td>Auburn</td></tr>
<tr><td>2</td><td>mainec___2</td><td>Augusta</td></tr>
<tr><td>3</td><td>mainec___3</td><td>Bangor</td></tr>
<tr><td>4</td><td>mainec___4</td><td>Bar Harbor</td></tr>
<tr><td>5</td><td>mainec___5</td><td>Bath</td></tr>
<tr><td>6</td><td>mainec___6</td><td>Belfast</td></tr>
<tr><td>7</td><td>mainec___7</td><td>Biddeford</td></tr>
<tr><td>8</td><td>mainec___8</td><td>Boothbay Harbor</td></tr>
<tr><td>9</td><td>mainec___9</td><td>Brunswick</td></tr>
<tr><td>10</td><td>mainec___10</td><td>Calais</td></tr>
<tr><td>11</td><td>mainec___11</td><td>Caribou</td></tr>
<tr><td>12</td><td>mainec___12</td><td>Castine</td></tr>
<tr><td>13</td><td>mainec___13</td><td>Eastport</td></tr>
<tr><td>14</td><td>mainec___14</td><td>Ellsworth</td></tr>
<tr><td>15</td><td>mainec___15</td><td>Farmington</td></tr>
<tr><td>16</td><td>mainec___16</td><td>Fort Kent</td></tr>
<tr><td>17</td><td>mainec___17</td><td>Gardiner</td></tr>
<tr><td>18</td><td>mainec___18</td><td>Houlton</td></tr>
<tr><td>19</td><td>mainec___19</td><td>Kennebunkport</td></tr>
<tr><td>20</td><td>mainec___20</td><td>Kittery</td></tr>
<tr><td>21</td><td>mainec___21</td><td>Lewiston</td></tr>
<tr><td>22</td><td>mainec___22</td><td>Lubec</td></tr>
<tr><td>23</td><td>mainec___23</td><td>Machias</td></tr>
<tr><td>24</td><td>mainec___24</td><td>Orono</td></tr>
<tr><td>25</td><td>mainec___25</td><td>Portland</td></tr>
<tr><td>26</td><td>mainec___26</td><td>Presque Isle</td></tr>
<tr><td>27</td><td>mainec___27</td><td>Rockland</td></tr>
<tr><td>28</td><td>mainec___28</td><td>Rumford</td></tr>
<tr><td>29</td><td>mainec___29</td><td>Saco</td></tr>
<tr><td>30</td><td>mainec___30</td><td>Scarborough</td></tr>
<tr><td>31</td><td>mainec___31</td><td>Waterville</td></tr>
<tr><td>32</td><td>mainec___32</td><td>York</td></tr>
<tr><td>33</td><td>mainec___33</td><td>Not listed</td></tr>
</table>

Custom alignment: LH</td></tr>
</table>

	113	marylandc<br><br>Show the field ONLY if:<br>[states(20)] = '1'	Which cities in Maryland?	checkbox

1	marylandc___1	Aberdeen
2	marylandc___2	Annapolis
3	marylandc___3	Baltimore
4	marylandc___4	Bethesda-Chevy Chase
5	marylandc___5	Bowie
6	marylandc___6	Cambridge
7	marylandc___7	Catonsville
8	marylandc___8	College Park
9	marylandc___9	Columbia
10	marylandc___10	Cumberland
11	marylandc___11	Easton
12	marylandc___12	Elkton
13	marylandc___13	Emmitsburg
14	marylandc___14	Frederick
15	marylandc___15	Greenbelt
16	marylandc___16	Hagerstown
17	marylandc___17	Hyattsville
18	marylandc___18	Laurel
19	marylandc___19	Oakland
20	marylandc___20	Ocean City
21	marylandc___21	Rockville
22	marylandc___22	Saint Marys City
23	marylandc___23	Salisbury
24	marylandc___24	Silver Spring
25	marylandc___25	Takoma Park
26	marylandc___26	Towson
27	marylandc___27	Westminster
28	marylandc___28	Not listed

Custom alignment: LH

	114	massachusettsc<br><br>Show the field ONLY if:<br>[states(21)] = '1'	Which cities in Massachusetts?	checkbox

1	massachusettsc___1	Abington
2	massachusettsc___2	Adams
3	massachusettsc___3	Amesbury
4	massachusettsc___4	Amherst
5	massachusettsc___5	Andover
6	massachusettsc___6	Arlington
7	massachusettsc___7	Athol
8	massachusettsc___8	Attleboro
9	massachusettsc___9	Barnstable
10	massachusettsc___10	Bedford
11	massachusettsc___11	Beverly
12	massachusettsc___12	Boston
13	massachusettsc___13	Bourne
14	massachusettsc___14	Braintree
15	massachusettsc___15	Brockton
16	massachusettsc___16	Brookline
17	massachusettsc___17	Cambridge

18	massachusettsc___18	Canton
19	massachusettsc___19	Charlestown
20	massachusettsc___20	Chelmsford
21	massachusettsc___21	Chelsea
22	massachusettsc___22	Chicopee
23	massachusettsc___23	Clinton
24	massachusettsc___24	Cohasset
25	massachusettsc___25	Concord
26	massachusettsc___26	Danvers
27	massachusettsc___27	Dartmouth
28	massachusettsc___28	Dedham
29	massachusettsc___29	Dennis
30	massachusettsc___30	Duxbury
31	massachusettsc___31	Eastham
32	massachusettsc___32	Edgartown
33	massachusettsc___33	Everett
34	massachusettsc___34	Fairhaven
35	massachusettsc___35	Fall River
36	massachusettsc___36	Falmouth
37	massachusettsc___37	Fitchburg
38	massachusettsc___38	Framingham
39	massachusettsc___39	Gloucester
40	massachusettsc___40	Great Barrington
41	massachusettsc___41	Greenfield
42	massachusettsc___42	Groton
43	massachusettsc___43	Harwich
44	massachusettsc___44	Haverhill
45	massachusettsc___45	Hingham
46	massachusettsc___46	Holyoke
47	massachusettsc___47	Hyannis
48	massachusettsc___48	Ipswich
49	massachusettsc___49	Lawrence
50	massachusettsc___50	Lenox
51	massachusettsc___51	Leominster
52	massachusettsc___52	Lexington
53	massachusettsc___53	Lowell
54	massachusettsc___54	Ludlow
55	massachusettsc___55	Lynn
56	massachusettsc___56	Malden
57	massachusettsc___57	Marblehead
58	massachusettsc___58	Marlborough
59	massachusettsc___59	Medford
60	massachusettsc___60	Milton
61	massachusettsc___61	Nahant
62	massachusettsc___62	Natick
63	massachusettsc___63	New Bedford
64	massachusettsc___64	Newburyport
65	massachusettsc___65	Newton

66	massachusettsc___66	North Adams
67	massachusettsc___67	Northampton
68	massachusettsc___68	Norton
69	massachusettsc___69	Norwood
70	massachusettsc___70	Peabody
71	massachusettsc___71	Pittsfield
72	massachusettsc___72	Plymouth
73	massachusettsc___73	Provincetown
74	massachusettsc___74	Quincy
75	massachusettsc___75	Randolph
76	massachusettsc___76	Revere
77	massachusettsc___77	Salem
78	massachusettsc___78	Sandwich
79	massachusettsc___79	Saugus
80	massachusettsc___80	Somerville
81	massachusettsc___81	South Hadley
82	massachusettsc___82	Springfield
83	massachusettsc___83	Stockbridge
84	massachusettsc___84	Stoughton
85	massachusettsc___85	Sturbridge
86	massachusettsc___86	Sudbury
87	massachusettsc___87	Taunton
88	massachusettsc___88	Tewksbury
89	massachusettsc___89	Truro
90	massachusettsc___90	Watertown
91	massachusettsc___91	Webster
92	massachusettsc___92	Wellesley
93	massachusettsc___93	Wellfleet
94	massachusettsc___94	West Bridgewater
95	massachusettsc___95	West Springfield
96	massachusettsc___96	Westfield
97	massachusettsc___97	Weymouth
98	massachusettsc___98	Whitman
99	massachusettsc___99	Williamstown
100	massachusettsc___100	Woburn
101	massachusettsc___101	Woods Hole
102	massachusettsc___102	Worcester
103	massachusettsc___103	Not listed

Custom alignment: LH

	115	michiganc<br>Show the field ONLY if:<br>[states(22)] = '1'	Which cities in Michigan?	checkbox

1	michiganc___1	Adrian
2	michiganc___2	Alma
3	michiganc___3	Ann Arbor
4	michiganc___4	Battle Creek
5	michiganc___5	Bay City
6	michiganc___6	Benton Harbor
7	michiganc___7	Bloomfield Hills
8	michiganc___8	Cadillac

9	michiganc___9	Charlevoix
10	michiganc___10	Cheboygan
11	michiganc___11	Dearborn
12	michiganc___12	Detroit
13	michiganc___13	East Lansing
14	michiganc___14	Eastpointe
15	michiganc___15	Ecorse
16	michiganc___16	Escanaba
17	michiganc___17	Flint
18	michiganc___18	Grand Haven
19	michiganc___19	Grand Rapids
20	michiganc___20	Grayling
21	michiganc___21	Grosse Pointe
22	michiganc___22	Hancock
23	michiganc___23	Highland Park
24	michiganc___24	Holland
25	michiganc___25	Houghton
26	michiganc___26	Interlochen
27	michiganc___27	Iron Mountain
28	michiganc___28	Ironwood
29	michiganc___29	Ishpeming
30	michiganc___30	Jackson
31	michiganc___31	Kalamazoo
32	michiganc___32	Lansing
33	michiganc___33	Livonia
34	michiganc___34	Ludington
35	michiganc___35	Mackinaw City
36	michiganc___36	Manistee
37	michiganc___37	Marquette
38	michiganc___38	Menominee
39	michiganc___39	Midland
40	michiganc___40	Monroe
41	michiganc___41	Mount Clemens
42	michiganc___42	Mount Pleasant
43	michiganc___43	Muskegon
44	michiganc___44	Niles
45	michiganc___45	Petoskey
46	michiganc___46	Pontiac
47	michiganc___47	Port Huron
48	michiganc___48	Royal Oak
49	michiganc___49	Saginaw
50	michiganc___50	Saint Ignace
51	michiganc___51	Saint Joseph
52	michiganc___52	Sault Sainte Marie
53	michiganc___53	Traverse City
54	michiganc___54	Trenton
55	michiganc___55	Warren
56	michiganc___56	Wyandotte

<table>
<tr><td></td><td></td><td></td><td></td><td><table><tr><td>57</td><td>michiganc___57</td><td>Ypsilanti</td></tr><tr><td>58</td><td>michiganc___58</td><td>Not listed</td></tr></table>
Custom alignment: LH</td></tr>
<tr><td></td><td>116</td><td>minnesotac

Show the field ONLY if:
[states(23)] = '1'</td><td>Which cities in Minnesota?</td><td>checkbox<table>
<tr><td>1</td><td>minnesotac___1</td><td>Albert Lea</td></tr>
<tr><td>2</td><td>minnesotac___2</td><td>Alexandria</td></tr>
<tr><td>3</td><td>minnesotac___3</td><td>Austin</td></tr>
<tr><td>4</td><td>minnesotac___4</td><td>Bemidji</td></tr>
<tr><td>5</td><td>minnesotac___5</td><td>Bloomington</td></tr>
<tr><td>6</td><td>minnesotac___6</td><td>Brainerd</td></tr>
<tr><td>7</td><td>minnesotac___7</td><td>Crookston</td></tr>
<tr><td>8</td><td>minnesotac___8</td><td>Duluth</td></tr>
<tr><td>9</td><td>minnesotac___9</td><td>Ely</td></tr>
<tr><td>10</td><td>minnesotac___10</td><td>Eveleth</td></tr>
<tr><td>11</td><td>minnesotac___11</td><td>Faribault</td></tr>
<tr><td>12</td><td>minnesotac___12</td><td>Fergus Falls</td></tr>
<tr><td>13</td><td>minnesotac___13</td><td>Hastings</td></tr>
<tr><td>14</td><td>minnesotac___14</td><td>Hibbing</td></tr>
<tr><td>15</td><td>minnesotac___15</td><td>International Falls</td></tr>
<tr><td>16</td><td>minnesotac___16</td><td>Little Falls</td></tr>
<tr><td>17</td><td>minnesotac___17</td><td>Mankato</td></tr>
<tr><td>18</td><td>minnesotac___18</td><td>Minneapolis</td></tr>
<tr><td>19</td><td>minnesotac___19</td><td>Moorhead</td></tr>
<tr><td>20</td><td>minnesotac___20</td><td>New Ulm</td></tr>
<tr><td>21</td><td>minnesotac___21</td><td>Northfield</td></tr>
<tr><td>22</td><td>minnesotac___22</td><td>Owatonna</td></tr>
<tr><td>23</td><td>minnesotac___23</td><td>Pipestone</td></tr>
<tr><td>24</td><td>minnesotac___24</td><td>Red Wing</td></tr>
<tr><td>25</td><td>minnesotac___25</td><td>Rochester</td></tr>
<tr><td>26</td><td>minnesotac___26</td><td>Saint Cloud</td></tr>
<tr><td>27</td><td>minnesotac___27</td><td>Saint Paul</td></tr>
<tr><td>28</td><td>minnesotac___28</td><td>Sauk Centre</td></tr>
<tr><td>29</td><td>minnesotac___29</td><td>South Saint Paul</td></tr>
<tr><td>30</td><td>minnesotac___30</td><td>Stillwater</td></tr>
<tr><td>31</td><td>minnesotac___31</td><td>Virginia</td></tr>
<tr><td>32</td><td>minnesotac___32</td><td>Willmar</td></tr>
<tr><td>33</td><td>minnesotac___33</td><td>Winona</td></tr>
</table>
Custom alignment: LH</td></tr>
</table>

	117	mississippic<br><br>Show the field ONLY if:<br>[states(24)] = '1'	Which cities in Mississippi?	checkbox<br>1 mississippic___1 Bay Saint Louis<br>2 mississippic___2 Biloxi<br>3 mississippic___3 Canton<br>4 mississippic___4 Clarksdale<br>5 mississippic___5 Columbia<br>6 mississippic___6 Columbus<br>7 mississippic___7 Corinth<br>8 mississippic___8 Greenville<br>9 mississippic___9 Greenwood<br>10 mississippic___10 Grenada<br>11 mississippic___11 Gulfport<br>12 mississippic___12 Hattiesburg<br>13 mississippic___13 Holly Springs<br>14 mississippic___14 Jackson<br>15 mississippic___15 Laurel<br>16 mississippic___16 Meridian<br>17 mississippic___17 Natchez<br>18 mississippic___18 Ocean Springs<br>19 mississippic___19 Oxford<br>20 mississippic___20 Pascagoula<br>21 mississippic___21 Pass Christian<br>22 mississippic___22 Philadelphia<br>23 mississippic___23 Port Gibson<br>24 mississippic___24 Starkville<br>25 mississippic___25 Tupelo<br>26 mississippic___26 Vicksburg<br>27 mississippic___27 West Point<br>28 mississippic___28 Yazoo City<br>29 mississippic___29 Not listed<br><br>Custom alignment: LH

	118	missouric<br><br>Show the field ONLY if:<br>[states(25)] = '1'	Which cities in Missouri?	checkbox<br>1 missouric___1 Boonville<br>2 missouric___2 Branson<br>3 missouric___3 Cape Girardeau<br>4 missouric___4 Carthage<br>5 missouric___5 Chillicothe<br>6 missouric___6 Clayton<br>7 missouric___7 Columbia<br>8 missouric___8 Excelsior Springs<br>9 missouric___9 Ferguson<br>10 missouric___10 Florissant<br>11 missouric___11 Fulton<br>12 missouric___12 Hannibal<br>13 missouric___13 Independence<br>14 missouric___14 Jefferson City<br>15 missouric___15 Joplin<br>16 missouric___16 Kansas City<br>17 missouric___17 Kirksville<br>18 missouric___18 Lamar<br>19 missouric___19 Lebanon<br>20 missouric___20 Lexington<br>21 missouric___21 Maryville<br>22 missouric___22 Mexico<br>23 missouric___23 Monett<br>24 missouric___24 Neosho<br>25 missouric___25 New Madrid<br>26 missouric___26 Rolla<br>27 missouric___27 Saint Charles<br>28 missouric___28 Saint Joseph<br>29 missouric___29 Saint Louis<br>30 missouric___30 Sainte Genevieve<br>31 missouric___31 Salem<br>32 missouric___32 Sedalia<br>33 missouric___33 Springfield<br>34 missouric___34 Warrensburg<br>35 missouric___35 West Plains<br>36 missouric___36 Not listed<br><br>Custom alignment: LH

	119	montanac<br><br>Show the field ONLY if:<br>[states(26)] = '1'	Which cities in Montana?	checkbox<br>1 montanac___1 Anaconda<br>2 montanac___2 Billings<br>3 montanac___3 Bozeman<br>4 montanac___4 Butte<br>5 montanac___5 Dillon<br>6 montanac___6 Fort Benton<br>7 montanac___7 Glendive<br>8 montanac___8 Great Falls<br>9 montanac___9 Havre<br>10 montanac___10 Helena<br>11 montanac___11 Kalispell<br>12 montanac___12 Lewistown<br>13 montanac___13 Livingston<br>14 montanac___14 Miles City<br>15 montanac___15 Missoula<br>16 montanac___16 Virginia City<br>17 montanac___17 Not listed<br><br>Custom alignment: LH
	120	nebraskac<br><br>Show the field ONLY if:<br>[states(27)] = '1'	Which cities in Nebraska?	checkbox<br>1 nebraskac___1 Beatrice<br>2 nebraskac___2 Bellevue<br>3 nebraskac___3 Boys Town<br>4 nebraskac___4 Chadron<br>5 nebraskac___5 Columbus<br>6 nebraskac___6 Fremont<br>7 nebraskac___7 Grand Island<br>8 nebraskac___8 Hastings<br>9 nebraskac___9 Kearney<br>10 nebraskac___10 Lincoln<br>11 nebraskac___11 McCook<br>12 nebraskac___12 Minden<br>13 nebraskac___13 Nebraska City<br>14 nebraskac___14 Norfolk<br>15 nebraskac___15 North Platte<br>16 nebraskac___16 Omaha<br>17 nebraskac___17 Plattsmouth<br>18 nebraskac___18 Red Cloud<br>19 nebraskac___19 Sidney<br>20 nebraskac___20 Not listed<br><br>Custom alignment: LH

<table>
<tr><td></td><td>121</td><td>nevadac

Show the field ONLY if:
[states(28)] = '1'</td><td>Which cities in Nevada?</td><td>checkbox<table>
<tr><td>1</td><td>nevadac___1</td><td>Boulder City</td></tr>
<tr><td>2</td><td>nevadac___2</td><td>Carson City</td></tr>
<tr><td>3</td><td>nevadac___3</td><td>Elko</td></tr>
<tr><td>4</td><td>nevadac___4</td><td>Ely</td></tr>
<tr><td>5</td><td>nevadac___5</td><td>Fallon</td></tr>
<tr><td>6</td><td>nevadac___6</td><td>Genoa</td></tr>
<tr><td>7</td><td>nevadac___7</td><td>Goldfield</td></tr>
<tr><td>8</td><td>nevadac___8</td><td>Henderson</td></tr>
<tr><td>9</td><td>nevadac___9</td><td>Las Vegas</td></tr>
<tr><td>10</td><td>nevadac___10</td><td>North Las Vegas</td></tr>
<tr><td>11</td><td>nevadac___11</td><td>Reno</td></tr>
<tr><td>12</td><td>nevadac___12</td><td>Sparks</td></tr>
<tr><td>13</td><td>nevadac___13</td><td>Virginia City</td></tr>
<tr><td>14</td><td>nevadac___14</td><td>Winnemucca</td></tr>
<tr><td>15</td><td>nevadac___15</td><td>Not listed</td></tr>
</table>Custom alignment: LH</td></tr>
<tr><td></td><td>122</td><td>newhampc

Show the field ONLY if:
[states(29)] = '1'</td><td>Which cities in New Hampshire?</td><td>checkbox<table>
<tr><td>1</td><td>newhampc___1</td><td>Berlin</td></tr>
<tr><td>2</td><td>newhampc___2</td><td>Claremont</td></tr>
<tr><td>3</td><td>newhampc___3</td><td>Concord</td></tr>
<tr><td>4</td><td>newhampc___4</td><td>Derry</td></tr>
<tr><td>5</td><td>newhampc___5</td><td>Dover</td></tr>
<tr><td>6</td><td>newhampc___6</td><td>Durham</td></tr>
<tr><td>7</td><td>newhampc___7</td><td>Exeter</td></tr>
<tr><td>8</td><td>newhampc___8</td><td>Franklin</td></tr>
<tr><td>9</td><td>newhampc___9</td><td>Hanover</td></tr>
<tr><td>10</td><td>newhampc___10</td><td>Hillsborough</td></tr>
<tr><td>11</td><td>newhampc___11</td><td>Keene</td></tr>
<tr><td>12</td><td>newhampc___12</td><td>Laconia</td></tr>
<tr><td>13</td><td>newhampc___13</td><td>Lebanon</td></tr>
<tr><td>14</td><td>newhampc___14</td><td>Manchester</td></tr>
<tr><td>15</td><td>newhampc___15</td><td>Nashua</td></tr>
<tr><td>16</td><td>newhampc___16</td><td>Peterborough</td></tr>
<tr><td>17</td><td>newhampc___17</td><td>Plymouth</td></tr>
<tr><td>18</td><td>newhampc___18</td><td>Portsmouth</td></tr>
<tr><td>19</td><td>newhampc___19</td><td>Rochester</td></tr>
<tr><td>20</td><td>newhampc___20</td><td>Salem</td></tr>
<tr><td>21</td><td>newhampc___21</td><td>Somersworth</td></tr>
<tr><td>22</td><td>newhampc___22</td><td>Not listed</td></tr>
</table>Custom alignment: LH</td></tr>
<tr><td></td><td>123</td><td>newjerseyc

Show the field ONLY if:
[states(30)] = '1'</td><td>Which cities in New Jersey?</td><td>checkbox<table>
<tr><td>1</td><td>newjerseyc___1</td><td>Asbury Park</td></tr>
<tr><td>2</td><td>newjerseyc___2</td><td>Atlantic City</td></tr>
<tr><td>3</td><td>newjerseyc___3</td><td>Bayonne</td></tr>
<tr><td>4</td><td>newjerseyc___4</td><td>Bloomfield</td></tr>
<tr><td>5</td><td>newjerseyc___5</td><td>Bordentown</td></tr>
<tr><td>6</td><td>newjerseyc___6</td><td>Bound Brook</td></tr>
</table></td></tr>
</table>

7	newjerseyc___7	Bridgeton
8	newjerseyc___8	Burlington
9	newjerseyc___9	Caldwell
10	newjerseyc___10	Camden
11	newjerseyc___11	Cape May
12	newjerseyc___12	Clifton
13	newjerseyc___13	Cranford
14	newjerseyc___14	East Orange
15	newjerseyc___15	Edison
16	newjerseyc___16	Elizabeth
17	newjerseyc___17	Englewood
18	newjerseyc___18	Fort Lee
19	newjerseyc___19	Glassboro
20	newjerseyc___20	Hackensack
21	newjerseyc___21	Haddonfield
22	newjerseyc___22	Hoboken
23	newjerseyc___23	Irvington
24	newjerseyc___24	Jersey City
25	newjerseyc___25	Lakehurst
26	newjerseyc___26	Lakewood
27	newjerseyc___27	Long Beach
28	newjerseyc___28	Long Branch
29	newjerseyc___29	Madison
30	newjerseyc___30	Menlo Park
31	newjerseyc___31	Millburn
32	newjerseyc___32	Millville
33	newjerseyc___33	Montclair
34	newjerseyc___34	Morristown
35	newjerseyc___35	Mount Holly
36	newjerseyc___36	New Brunswick
37	newjerseyc___37	New Milford
38	newjerseyc___38	Newark
39	newjerseyc___39	Ocean City
40	newjerseyc___40	Orange
41	newjerseyc___41	Parsippany-Troy Hills
42	newjerseyc___42	Passaic
43	newjerseyc___43	Paterson
44	newjerseyc___44	Perth Amboy
45	newjerseyc___45	Plainfield
46	newjerseyc___46	Princeton
47	newjerseyc___47	Ridgewood
48	newjerseyc___48	Roselle
49	newjerseyc___49	Rutherford
50	newjerseyc___50	Salem
51	newjerseyc___51	Somerville
52	newjerseyc___52	South Orange Village
53	newjerseyc___53	Totowa
54	newjerseyc___54	Trenton

				<table><tr><td>55</td><td>newjerseyc___55</td><td>Union</td></tr><tr><td>56</td><td>newjerseyc___56</td><td>Union City</td></tr><tr><td>57</td><td>newjerseyc___57</td><td>Vineland</td></tr><tr><td>58</td><td>newjerseyc___58</td><td>Wayne</td></tr><tr><td>59</td><td>newjerseyc___59</td><td>Weehawken</td></tr><tr><td>60</td><td>newjerseyc___60</td><td>West New York</td></tr><tr><td>61</td><td>newjerseyc___61</td><td>West Orange</td></tr><tr><td>62</td><td>newjerseyc___62</td><td>Willingboro</td></tr><tr><td>63</td><td>newjerseyc___63</td><td>Woodbridge</td></tr><tr><td>64</td><td>newjerseyc___64</td><td>Not listed</td></tr></table><br>Custom alignment: LH
	124	newmexc<br>Show the field ONLY if:<br>[states(31)] = '1'	Which cities in New Mexico?	checkbox<table><tr><td>1</td><td>newmexc___1</td><td>Acoma</td></tr><tr><td>2</td><td>newmexc___2</td><td>Alamogordo</td></tr><tr><td>3</td><td>newmexc___3</td><td>Albuquerque</td></tr><tr><td>4</td><td>newmexc___4</td><td>Artesia</td></tr><tr><td>5</td><td>newmexc___5</td><td>Belen</td></tr><tr><td>6</td><td>newmexc___6</td><td>Carlsbad</td></tr><tr><td>7</td><td>newmexc___7</td><td>Clovis</td></tr><tr><td>8</td><td>newmexc___8</td><td>Deming</td></tr><tr><td>9</td><td>newmexc___9</td><td>Farmington</td></tr><tr><td>10</td><td>newmexc___10</td><td>Gallup</td></tr><tr><td>11</td><td>newmexc___11</td><td>Grants</td></tr><tr><td>12</td><td>newmexc___12</td><td>Hobbs</td></tr><tr><td>13</td><td>newmexc___13</td><td>Las Cruces</td></tr><tr><td>14</td><td>newmexc___14</td><td>Las Vegas</td></tr><tr><td>15</td><td>newmexc___15</td><td>Los Alamos</td></tr><tr><td>16</td><td>newmexc___16</td><td>Lovington</td></tr><tr><td>17</td><td>newmexc___17</td><td>Portales</td></tr><tr><td>18</td><td>newmexc___18</td><td>Raton</td></tr><tr><td>19</td><td>newmexc___19</td><td>Roswell</td></tr><tr><td>20</td><td>newmexc___20</td><td>Santa Fe</td></tr><tr><td>21</td><td>newmexc___21</td><td>Shiprock</td></tr><tr><td>22</td><td>newmexc___22</td><td>Silver City</td></tr><tr><td>23</td><td>newmexc___23</td><td>Socorro</td></tr><tr><td>24</td><td>newmexc___24</td><td>Taos</td></tr><tr><td>25</td><td>newmexc___25</td><td>Truth or Consequences</td></tr><tr><td>26</td><td>newmexc___26</td><td>Tucumcari</td></tr><tr><td>27</td><td>newmexc___27</td><td>Not listed</td></tr></table><br>Custom alignment: LH
	125	newyorkc<br>Show the field ONLY if:<br>[states(32)] = '1'	Which cities in New York?	checkbox<table><tr><td>1</td><td>newyorkc___1</td><td>Albany</td></tr><tr><td>2</td><td>newyorkc___2</td><td>Amsterdam</td></tr><tr><td>3</td><td>newyorkc___3</td><td>Auburn</td></tr><tr><td>4</td><td>newyorkc___4</td><td>Babylon</td></tr><tr><td>5</td><td>newyorkc___5</td><td>Batavia</td></tr><tr><td>6</td><td>newyorkc___6</td><td>Beacon</td></tr></table>

7	newyorkc___7	Bedford
8	newyorkc___8	Binghamton
9	newyorkc___9	Bronx
10	newyorkc___10	Brooklyn
11	newyorkc___11	Buffalo
12	newyorkc___12	Chautauqua
13	newyorkc___13	Cheektowaga
14	newyorkc___14	Clinton
15	newyorkc___15	Cohoes
16	newyorkc___16	Coney Island
17	newyorkc___17	Cooperstown
18	newyorkc___18	Corning
19	newyorkc___19	Cortland
20	newyorkc___20	Crown Point
21	newyorkc___21	Dunkirk
22	newyorkc___22	East Aurora
23	newyorkc___23	East Hampton
24	newyorkc___24	Eastchester
25	newyorkc___25	Elmira
26	newyorkc___26	Flushing
27	newyorkc___27	Forest Hills
28	newyorkc___28	Fredonia
29	newyorkc___29	Garden City
30	newyorkc___30	Geneva
31	newyorkc___31	Glens Falls
32	newyorkc___32	Gloversville
33	newyorkc___33	Great Neck
34	newyorkc___34	Hammondsport
35	newyorkc___35	Harlem
36	newyorkc___36	Hempstead
37	newyorkc___37	Herkimer
38	newyorkc___38	Hudson
39	newyorkc___39	Huntington
40	newyorkc___40	Hyde Park
41	newyorkc___41	Ilion
42	newyorkc___42	Ithaca
43	newyorkc___43	Jamestown
44	newyorkc___44	Johnstown
45	newyorkc___45	Kingston
46	newyorkc___46	Lackawanna
47	newyorkc___47	Lake Placid
48	newyorkc___48	Levittown
49	newyorkc___49	Lockport
50	newyorkc___50	Mamaroneck
51	newyorkc___51	Manhattan
52	newyorkc___52	Massena
53	newyorkc___53	Middletown
54	newyorkc___54	Mineola

55	newyorkc___55	Mount Vernon
56	newyorkc___56	New Paltz
57	newyorkc___57	New Rochelle
58	newyorkc___58	New Windsor
59	newyorkc___59	New York City
60	newyorkc___60	Newburgh
61	newyorkc___61	Niagara Falls
62	newyorkc___62	North Hempstead
63	newyorkc___63	Nyack
64	newyorkc___64	Ogdensburg
65	newyorkc___65	Olean
66	newyorkc___66	Oneida
67	newyorkc___67	Oneonta
68	newyorkc___68	Ossining
69	newyorkc___69	Oswego
70	newyorkc___70	Oyster Bay
71	newyorkc___71	Palmyra
72	newyorkc___72	Peekskill
73	newyorkc___73	Plattsburgh
74	newyorkc___74	Port Washington
75	newyorkc___75	Potsdam
76	newyorkc___76	Poughkeepsie
77	newyorkc___77	Queens
78	newyorkc___78	Rensselaer
79	newyorkc___79	Rochester
80	newyorkc___80	Rome
81	newyorkc___81	Rotterdam
82	newyorkc___82	Rye
83	newyorkc___83	Sag Harbor
84	newyorkc___84	Saranac Lake
85	newyorkc___85	Saratoga Springs
86	newyorkc___86	Scarsdale
87	newyorkc___87	Schenectady
88	newyorkc___88	Seneca Falls
89	newyorkc___89	Southampton
90	newyorkc___90	Staten Island
91	newyorkc___91	Stony Brook
92	newyorkc___92	Stony Point
93	newyorkc___93	Syracuse
94	newyorkc___94	Tarrytown
95	newyorkc___95	Ticonderoga
96	newyorkc___96	Tonawanda
97	newyorkc___97	Troy
98	newyorkc___98	Utica
99	newyorkc___99	Watertown
100	newyorkc___100	Watervliet
101	newyorkc___101	Watkins Glen
102	newyorkc___102	West Seneca

				103 | newyorkc___103 | White Plains<br>104 | newyorkc___104 | Woodstock<br>105 | newyorkc___105 | Yonkers<br>106 | newyorkc___106 | Not listed<br><br>Custom alignment: LH
	126	northcarolinac<br><br>Show the field ONLY if:<br>[states(33)] = '1'	Which cities in North Carolina?	checkbox<br>1 | northcarolinac___1 | Asheboro<br>2 | northcarolinac___2 | Asheville<br>3 | northcarolinac___3 | Bath<br>4 | northcarolinac___4 | Beaufort<br>5 | northcarolinac___5 | Boone<br>6 | northcarolinac___6 | Burlington<br>7 | northcarolinac___7 | Chapel Hill<br>8 | northcarolinac___8 | Charlotte<br>9 | northcarolinac___9 | Concord<br>10 | northcarolinac___10 | Durham<br>11 | northcarolinac___11 | Edenton<br>12 | northcarolinac___12 | Elizabeth City<br>13 | northcarolinac___13 | Fayetteville<br>14 | northcarolinac___14 | Gastonia<br>15 | northcarolinac___15 | Goldsboro<br>16 | northcarolinac___16 | Greensboro<br>17 | northcarolinac___17 | Greenville<br>18 | northcarolinac___18 | Halifax<br>19 | northcarolinac___19 | Henderson<br>20 | northcarolinac___20 | Hickory<br>21 | northcarolinac___21 | High Point<br>22 | northcarolinac___22 | Hillsborough<br>23 | northcarolinac___23 | Jacksonville<br>24 | northcarolinac___24 | Kinston<br>25 | northcarolinac___25 | Kitty Hawk<br>26 | northcarolinac___26 | Lumberton<br>27 | northcarolinac___27 | Morehead City<br>28 | northcarolinac___28 | Morganton<br>29 | northcarolinac___29 | Nags Head<br>30 | northcarolinac___30 | New Bern<br>31 | northcarolinac___31 | Pinehurst<br>32 | northcarolinac___32 | Raleigh<br>33 | northcarolinac___33 | Rocky Mount<br>34 | northcarolinac___34 | Salisbury<br>35 | northcarolinac___35 | Shelby<br>36 | northcarolinac___36 | Washington<br>37 | northcarolinac___37 | Wilmington<br>38 | northcarolinac___38 | Wilson<br>39 | northcarolinac___39 | Winston-Salem<br>40 | northcarolinac___40 | Not listed<br><br>Custom alignment: LH

	127	northdakotac<br><br>Show the field ONLY if:<br>[states(34)] = '1'	Which cities in North Dakota?	checkbox<br>1 northdakotac___1 Bismarck<br>2 northdakotac___2 Devils Lake<br>3 northdakotac___3 Dickinson<br>4 northdakotac___4 Fargo<br>5 northdakotac___5 Grand Forks<br>6 northdakotac___6 Jamestown<br>7 northdakotac___7 Mandan<br>8 northdakotac___8 Minot<br>9 northdakotac___9 Rugby<br>10 northdakotac___10 Valley City<br>11 northdakotac___11 Wahpeton<br>12 northdakotac___12 Williston<br>13 northdakotac___13 Not listed<br><br>Custom alignment: LH
	128	ohioc<br><br>Show the field ONLY if:<br>[states(35)] = '1'	Which cities in Ohio?	checkbox<br>1 ohioc___1 Akron<br>2 ohioc___2 Alliance<br>3 ohioc___3 Ashtabula<br>4 ohioc___4 Athens<br>5 ohioc___5 Barberton<br>6 ohioc___6 Bedford<br>7 ohioc___7 Bellefontaine<br>8 ohioc___8 Bowling Green<br>9 ohioc___9 Canton<br>10 ohioc___10 Chillicothe<br>11 ohioc___11 Cincinnati<br>12 ohioc___12 Cleveland<br>13 ohioc___13 Cleveland Heights<br>14 ohioc___14 Columbus<br>15 ohioc___15 Conneaut<br>16 ohioc___16 Cuyahoga Falls<br>17 ohioc___17 Dayton<br>18 ohioc___18 Defiance<br>19 ohioc___19 Delaware<br>20 ohioc___20 East Cleveland<br>21 ohioc___21 East Liverpool<br>22 ohioc___22 Elyria<br>23 ohioc___23 Euclid<br>24 ohioc___24 Findlay<br>25 ohioc___25 Gallipolis<br>26 ohioc___26 Greenville<br>27 ohioc___27 Hamilton<br>28 ohioc___28 Kent<br>29 ohioc___29 Kettering<br>30 ohioc___30 Lakewood<br>31 ohioc___31 Lancaster<br>32 ohioc___32 Lima

				33, ohioc___33, Lorain<br>34, ohioc___34, Mansfield<br>35, ohioc___35, Marietta<br>36, ohioc___36, Marion<br>37, ohioc___37, Martins Ferry<br>38, ohioc___38, Massillon<br>39, ohioc___39, Mentor<br>40, ohioc___40, Middletown<br>41, ohioc___41, Milan<br>42, ohioc___42, Mount Vernon<br>43, ohioc___43, New Philadelphia<br>44, ohioc___44, Newark<br>45, ohioc___45, Niles<br>46, ohioc___46, North College Hill<br>47, ohioc___47, Norwalk<br>48, ohioc___48, Oberlin<br>49, ohioc___49, Painesville<br>50, ohioc___50, Parma<br>51, ohioc___51, Piqua<br>52, ohioc___52, Portsmouth<br>53, ohioc___53, Put-in-Bay<br>54, ohioc___54, Salem<br>55, ohioc___55, Sandusky<br>56, ohioc___56, Shaker Heights<br>57, ohioc___57, Springfield<br>58, ohioc___58, Steubenville<br>59, ohioc___59, Tiffin<br>60, ohioc___60, Toledo<br>61, ohioc___61, Urbana<br>62, ohioc___62, Warren<br>63, ohioc___63, Wooster<br>64, ohioc___64, Worthington<br>65, ohioc___65, Xenia<br>66, ohioc___66, Yellow Springs<br>67, ohioc___67, Youngstown<br>68, ohioc___68, Zanesville<br>69, ohioc___69, Not listed<br><br>Custom alignment: LH
	129	oklahomac<br><br>Show the field ONLY if:<br>[states(36)] = '1'	Which cities in Oklahoma?	checkbox<br>1, oklahomac___1, Ada<br>2, oklahomac___2, Altus<br>3, oklahomac___3, Alva<br>4, oklahomac___4, Anadarko<br>5, oklahomac___5, Ardmore<br>6, oklahomac___6, Bartlesville<br>7, oklahomac___7, Bethany<br>8, oklahomac___8, Chickasha<br>9, oklahomac___9, Claremore

10	oklahomac___10	Clinton
11	oklahomac___11	Cushing
12	oklahomac___12	Duncan
13	oklahomac___13	Durant
14	oklahomac___14	Edmond
15	oklahomac___15	El Reno
16	oklahomac___16	Elk City
17	oklahomac___17	Enid
18	oklahomac___18	Eufaula
19	oklahomac___19	Frederick
20	oklahomac___20	Guthrie
21	oklahomac___21	Guymon
22	oklahomac___22	Hobart
23	oklahomac___23	Holdenville
24	oklahomac___24	Hugo
25	oklahomac___25	Lawton
26	oklahomac___26	McAlester
27	oklahomac___27	Miami
28	oklahomac___28	Midwest City
29	oklahomac___29	Moore
30	oklahomac___30	Muskogee
31	oklahomac___31	Norman
32	oklahomac___32	Oklahoma City
33	oklahomac___33	Okmulgee
34	oklahomac___34	Pauls Valley
35	oklahomac___35	Pawhuska
36	oklahomac___36	Perry
37	oklahomac___37	Ponca City
38	oklahomac___38	Pryor
39	oklahomac___39	Sallisaw
40	oklahomac___40	Sand Springs
41	oklahomac___41	Sapulpa
42	oklahomac___42	Seminole
43	oklahomac___43	Shawnee
44	oklahomac___44	Stillwater
45	oklahomac___45	Tahlequah
46	oklahomac___46	The Village
47	oklahomac___47	Tulsa
48	oklahomac___48	Vinita
49	oklahomac___49	Wewoka
50	oklahomac___50	Woodward
51	oklahomac___51	Not listed

Custom alignment: LH

	130	oregonc<br><br>Show the field ONLY if:<br>[states(37)] = '1'	Which cities in Oregon?	checkbox<br>1 oregonc___1 Albany<br>2 oregonc___2 Ashland<br>3 oregonc___3 Astoria<br>4 oregonc___4 Baker City<br>5 oregonc___5 Beaverton<br>6 oregonc___6 Bend<br>7 oregonc___7 Brookings<br>8 oregonc___8 Burns<br>9 oregonc___9 Coos Bay<br>10 oregonc___10 Corvallis<br>11 oregonc___11 Eugene<br>12 oregonc___12 Grants Pass<br>13 oregonc___13 Hillsboro<br>14 oregonc___14 Hood River<br>15 oregonc___15 Jacksonville<br>16 oregonc___16 John Day<br>17 oregonc___17 Klamath Falls<br>18 oregonc___18 La Grande<br>19 oregonc___19 Lake Oswego<br>20 oregonc___20 Lakeview<br>21 oregonc___21 McMinnville<br>22 oregonc___22 Medford<br>23 oregonc___23 Newberg<br>24 oregonc___24 Newport<br>25 oregonc___25 Ontario<br>26 oregonc___26 Oregon City<br>27 oregonc___27 Pendleton<br>28 oregonc___28 Port Orford<br>29 oregonc___29 Portland<br>30 oregonc___30 Prineville<br>31 oregonc___31 Redmond<br>32 oregonc___32 Reedsport<br>33 oregonc___33 Roseburg<br>34 oregonc___34 Salem<br>35 oregonc___35 Seaside<br>36 oregonc___36 Springfield<br>37 oregonc___37 The Dalles<br>38 oregonc___38 Tillamook<br>39 oregonc___39 Not listed<br><br>Custom alignment: LH
	131	pennslyvaniac<br><br>Show the field ONLY if:<br>[states(38)] = '1'	Which cities in Pennsylvania?	checkbox<br>1 pennslyvaniac___1 Abington<br>2 pennslyvaniac___2 Aliquippa<br>3 pennslyvaniac___3 Allentown<br>4 pennslyvaniac___4 Altoona<br>5 pennslyvaniac___5 Ambridge<br>6 pennslyvaniac___6 Bedford

7	pennslyvaniac___7	Bethlehem
8	pennslyvaniac___8	Bloomsburg
9	pennslyvaniac___9	Bradford
10	pennslyvaniac___10	Bristol
11	pennslyvaniac___11	Carbondale
12	pennslyvaniac___12	Carlisle
13	pennslyvaniac___13	Chambersburg
14	pennslyvaniac___14	Chester
15	pennslyvaniac___15	Columbia
16	pennslyvaniac___16	Easton
17	pennslyvaniac___17	Erie
18	pennslyvaniac___18	Franklin
19	pennslyvaniac___19	Germantown
20	pennslyvaniac___20	Gettysburg
21	pennslyvaniac___21	Greensburg
22	pennslyvaniac___22	Hanover
23	pennslyvaniac___23	Harmony
24	pennslyvaniac___24	Harrisburg
25	pennslyvaniac___25	Hazleton
26	pennslyvaniac___26	Hershey
27	pennslyvaniac___27	Homestead
28	pennslyvaniac___28	Honesdale
29	pennslyvaniac___29	Indiana
30	pennslyvaniac___30	Jeannette
31	pennslyvaniac___31	Jim Thorpe
32	pennslyvaniac___32	Johnstown
33	pennslyvaniac___33	Lancaster
34	pennslyvaniac___34	Lebanon
35	pennslyvaniac___35	Levittown
36	pennslyvaniac___36	Lewistown
37	pennslyvaniac___37	Lock Haven
38	pennslyvaniac___38	Lower Southampton
39	pennslyvaniac___39	McKeesport
40	pennslyvaniac___40	Meadville
41	pennslyvaniac___41	Middletown
42	pennslyvaniac___42	Monroeville
43	pennslyvaniac___43	Nanticoke
44	pennslyvaniac___44	New Castle
45	pennslyvaniac___45	New Hope
46	pennslyvaniac___46	New Kensington
47	pennslyvaniac___47	Norristown
48	pennslyvaniac___48	Oil City
49	pennslyvaniac___49	Philadelphia
50	pennslyvaniac___50	Phoenixville
51	pennslyvaniac___51	Pittsburgh
52	pennslyvaniac___52	Pottstown
53	pennslyvaniac___53	Pottsville
54	pennslyvaniac___54	Reading

				<table><tr><td>55</td><td>pennslyvaniac___55</td><td>Scranton</td></tr><tr><td>56</td><td>pennslyvaniac___56</td><td>Shamokin</td></tr><tr><td>57</td><td>pennslyvaniac___57</td><td>Sharon</td></tr><tr><td>58</td><td>pennslyvaniac___58</td><td>State College</td></tr><tr><td>59</td><td>pennslyvaniac___59</td><td>Stroudsburg</td></tr><tr><td>60</td><td>pennslyvaniac___60</td><td>Sunbury</td></tr><tr><td>61</td><td>pennslyvaniac___61</td><td>Swarthmore</td></tr><tr><td>62</td><td>pennslyvaniac___62</td><td>Tamaqua</td></tr><tr><td>63</td><td>pennslyvaniac___63</td><td>Titusville</td></tr><tr><td>64</td><td>pennslyvaniac___64</td><td>Uniontown</td></tr><tr><td>65</td><td>pennslyvaniac___65</td><td>Warren</td></tr><tr><td>66</td><td>pennslyvaniac___66</td><td>Washington</td></tr><tr><td>67</td><td>pennslyvaniac___67</td><td>West Chester</td></tr><tr><td>68</td><td>pennslyvaniac___68</td><td>Wilkes-Barre</td></tr><tr><td>69</td><td>pennslyvaniac___69</td><td>Williamsport</td></tr><tr><td>70</td><td>pennslyvaniac___70</td><td>York</td></tr><tr><td>71</td><td>pennslyvaniac___71</td><td>Not listed</td></tr></table><br>Custom alignment: LH
	132	rhodeislandc<br><br>Show the field ONLY if:<br>[states(39)] = '1'	Which states in Rhode Island?	checkbox<table><tr><td>1</td><td>rhodeislandc___1</td><td>Barrington</td></tr><tr><td>2</td><td>rhodeislandc___2</td><td>Bristol</td></tr><tr><td>3</td><td>rhodeislandc___3</td><td>Central Falls</td></tr><tr><td>4</td><td>rhodeislandc___4</td><td>Cranston</td></tr><tr><td>5</td><td>rhodeislandc___5</td><td>East Greenwich</td></tr><tr><td>6</td><td>rhodeislandc___6</td><td>East Providence</td></tr><tr><td>7</td><td>rhodeislandc___7</td><td>Kingston</td></tr><tr><td>8</td><td>rhodeislandc___8</td><td>Middletown</td></tr><tr><td>9</td><td>rhodeislandc___9</td><td>Narragansett</td></tr><tr><td>10</td><td>rhodeislandc___10</td><td>Newport</td></tr><tr><td>11</td><td>rhodeislandc___11</td><td>North Kingstown</td></tr><tr><td>12</td><td>rhodeislandc___12</td><td>Pawtucket</td></tr><tr><td>13</td><td>rhodeislandc___13</td><td>Portsmouth</td></tr><tr><td>14</td><td>rhodeislandc___14</td><td>Providence</td></tr><tr><td>15</td><td>rhodeislandc___15</td><td>South Kingstown</td></tr><tr><td>16</td><td>rhodeislandc___16</td><td>Tiverton</td></tr><tr><td>17</td><td>rhodeislandc___17</td><td>Warren</td></tr><tr><td>18</td><td>rhodeislandc___18</td><td>Warwick</td></tr><tr><td>19</td><td>rhodeislandc___19</td><td>Westerly</td></tr><tr><td>20</td><td>rhodeislandc___20</td><td>Wickford</td></tr><tr><td>21</td><td>rhodeislandc___21</td><td>Woonsocket</td></tr><tr><td>22</td><td>rhodeislandc___22</td><td>Not listed</td></tr></table><br>Custom alignment: LH

<table>
<tr>
<td></td>
<td>133</td>
<td>scarolinac

Show the field ONLY if:
[states(40)] = '1'</td>
<td>Which cities in South Carolina?</td>
<td>checkbox
<table>
<tr><td>1</td><td>scarolinac___1</td><td>Abbeville</td></tr>
<tr><td>2</td><td>scarolinac___2</td><td>Aiken</td></tr>
<tr><td>3</td><td>scarolinac___3</td><td>Anderson</td></tr>
<tr><td>4</td><td>scarolinac___4</td><td>Beaufort</td></tr>
<tr><td>5</td><td>scarolinac___5</td><td>Camden</td></tr>
<tr><td>6</td><td>scarolinac___6</td><td>Charleston</td></tr>
<tr><td>7</td><td>scarolinac___7</td><td>Columbia</td></tr>
<tr><td>8</td><td>scarolinac___8</td><td>Darlington</td></tr>
<tr><td>9</td><td>scarolinac___9</td><td>Florence</td></tr>
<tr><td>10</td><td>scarolinac___10</td><td>Gaffney</td></tr>
<tr><td>11</td><td>scarolinac___11</td><td>Georgetown</td></tr>
<tr><td>12</td><td>scarolinac___12</td><td>Greenville</td></tr>
<tr><td>13</td><td>scarolinac___13</td><td>Greenwood</td></tr>
<tr><td>14</td><td>scarolinac___14</td><td>Hartsville</td></tr>
<tr><td>15</td><td>scarolinac___15</td><td>Lancaster</td></tr>
<tr><td>16</td><td>scarolinac___16</td><td>Mount Pleasant</td></tr>
<tr><td>17</td><td>scarolinac___17</td><td>Myrtle Beach</td></tr>
<tr><td>18</td><td>scarolinac___18</td><td>Orangeburg</td></tr>
<tr><td>19</td><td>scarolinac___19</td><td>Rock Hill</td></tr>
<tr><td>20</td><td>scarolinac___20</td><td>Spartanburg</td></tr>
<tr><td>21</td><td>scarolinac___21</td><td>Sumter</td></tr>
<tr><td>22</td><td>scarolinac___22</td><td>Union</td></tr>
<tr><td>23</td><td>scarolinac___23</td><td>Not listed</td></tr>
</table>

Custom alignment: LH</td>
</tr>
</table>

	134	sdakotac<br><br>Show the field ONLY if:<br>[states(41)] = '1'	Which cities in South Dakota?	checkbox<br>1 sdakotac___1 Aberdeen<br>2 sdakotac___2 Belle Fourche<br>3 sdakotac___3 Brookings<br>4 sdakotac___4 Canton<br>5 sdakotac___5 Custer<br>6 sdakotac___6 De Smet<br>7 sdakotac___7 Deadwood<br>8 sdakotac___8 Hot Springs<br>9 sdakotac___9 Huron<br>10 sdakotac___10 Lead<br>11 sdakotac___11 Madison<br>12 sdakotac___12 Milbank<br>13 sdakotac___13 Mitchell<br>14 sdakotac___14 Mobridge<br>15 sdakotac___15 Pierre<br>16 sdakotac___16 Rapid City<br>17 sdakotac___17 Sioux Falls<br>18 sdakotac___18 Spearfish<br>19 sdakotac___19 Sturgis<br>20 sdakotac___20 Vermillion<br>21 sdakotac___21 Watertown<br>22 sdakotac___22 Yankton<br>23 sdakotac___23 Not listed<br><br>Custom alignment: LH

	135	tennesseec<br><br>Show the field ONLY if:<br>[states(42)] = '1'	Which cities in Tennessee?	checkbox<br>1 tennesseec___1 Alcoa<br>2 tennesseec___2 Athens<br>3 tennesseec___3 Chattanooga<br>4 tennesseec___4 Clarksville<br>5 tennesseec___5 Cleveland<br>6 tennesseec___6 Columbia<br>7 tennesseec___7 Cookeville<br>8 tennesseec___8 Dayton<br>9 tennesseec___9 Elizabethton<br>10 tennesseec___10 Franklin<br>11 tennesseec___11 Gallatin<br>12 tennesseec___12 Gatlinburg<br>13 tennesseec___13 Greeneville<br>14 tennesseec___14 Jackson<br>15 tennesseec___15 Johnson City<br>16 tennesseec___16 Jonesborough<br>17 tennesseec___17 Kingsport<br>18 tennesseec___18 Knoxville<br>19 tennesseec___19 Lebanon<br>20 tennesseec___20 Maryville<br>21 tennesseec___21 Memphis<br>22 tennesseec___22 Morristown<br>23 tennesseec___23 Murfreesboro<br>24 tennesseec___24 Nashville<br>25 tennesseec___25 Norris<br>26 tennesseec___26 Oak Ridge<br>27 tennesseec___27 Shelbyville<br>28 tennesseec___28 Tullahoma<br>29 tennesseec___29 Not listed<br><br>Custom alignment: LH
	136	texasc<br><br>Show the field ONLY if:<br>[states(43)] = '1'	Which cities in Texas?	checkbox<br>1 texasc___1 Abilene<br>2 texasc___2 Alpine<br>3 texasc___3 Amarillo<br>4 texasc___4 Arlington<br>5 texasc___5 Austin<br>6 texasc___6 Baytown<br>7 texasc___7 Beaumont<br>8 texasc___8 Big Spring<br>9 texasc___9 Borger<br>10 texasc___10 Brownsville<br>11 texasc___11 Bryan<br>12 texasc___12 Canyon<br>13 texasc___13 Cleburne<br>14 texasc___14 College Station<br>15 texasc___15 Corpus Christi<br>16 texasc___16 Crystal City

17	texasc___17	Dallas
18	texasc___18	Del Rio
19	texasc___19	Denison
20	texasc___20	Denton
21	texasc___21	Eagle Pass
22	texasc___22	Edinburg
23	texasc___23	El Paso
24	texasc___24	Fort Worth
25	texasc___25	Freeport
26	texasc___26	Galveston
27	texasc___27	Garland
28	texasc___28	Goliad
29	texasc___29	Greenville
30	texasc___30	Harlingen
31	texasc___31	Houston
32	texasc___32	Huntsville
33	texasc___33	Irving
34	texasc___34	Johnson City
35	texasc___35	Kilgore
36	texasc___36	Killeen
37	texasc___37	Kingsville
38	texasc___38	Laredo
39	texasc___39	Longview
40	texasc___40	Lubbock
41	texasc___41	Lufkin
42	texasc___42	Marshall
43	texasc___43	McAllen
44	texasc___44	McKinney
45	texasc___45	Mesquite
46	texasc___46	Midland
47	texasc___47	Mission
48	texasc___48	Nacogdoches
49	texasc___49	New Braunfels
50	texasc___50	Odessa
51	texasc___51	Orange
52	texasc___52	Pampa
53	texasc___53	Paris
54	texasc___54	Pasadena
55	texasc___55	Pecos
56	texasc___56	Pharr
57	texasc___57	Plainview
58	texasc___58	Plano
59	texasc___59	Port Arthur
60	texasc___60	Port Lavaca
61	texasc___61	Richardson
62	texasc___62	San Angelo
63	texasc___63	San Antonio
64	texasc___64	San Felipe

65	texasc___65	San Marcos
66	texasc___66	Sherman
67	texasc___67	Sweetwater
68	texasc___68	Temple
69	texasc___69	Texarkana
70	texasc___70	Texas City
71	texasc___71	Tyler
72	texasc___72	Uvalde
73	texasc___73	Victoria
74	texasc___74	Waco
75	texasc___75	Weatherford
76	texasc___76	Wichita Falls
77	texasc___77	Ysleta
78	texasc___78	Not listed

Custom alignment: LH

	137	utahc<br><br>Show the field ONLY if:<br>[states(44)] = '1'	Which cities in Utah?	checkbox<br>1 utahc___1 Alta<br>2 utahc___2 American Fork<br>3 utahc___3 Bountiful<br>4 utahc___4 Brigham City<br>5 utahc___5 Cedar City<br>6 utahc___6 Clearfield<br>7 utahc___7 Delta<br>8 utahc___8 Fillmore<br>9 utahc___9 Green River<br>10 utahc___10 Heber City<br>11 utahc___11 Kanab<br>12 utahc___12 Layton<br>13 utahc___13 Lehi<br>14 utahc___14 Logan<br>15 utahc___15 Manti<br>16 utahc___16 Moab<br>17 utahc___17 Monticello<br>18 utahc___18 Murray<br>19 utahc___19 Nephi<br>20 utahc___20 Ogden<br>21 utahc___21 Orderville<br>22 utahc___22 Orem<br>23 utahc___23 Panguitch<br>24 utahc___24 Park City<br>25 utahc___25 Payson<br>26 utahc___26 Price<br>27 utahc___27 Provo<br>28 utahc___28 Saint George<br>29 utahc___29 Salt Lake City<br>30 utahc___30 Spanish Fork<br>31 utahc___31 Springville<br>32 utahc___32 Tooele<br>33 utahc___33 Vernal<br>34 utahc___34 Not listed<br><br>Custom alignment: LH

	138	vermontc<br><br>Show the field ONLY if:<br>[states(45)] = '1'	Which cities in Vermont?	checkbox<br>1 vermontc___1 Barre<br>2 vermontc___2 Bellows Falls<br>3 vermontc___3 Bennington<br>4 vermontc___4 Brattleboro<br>5 vermontc___5 Burlington<br>6 vermontc___6 Essex<br>7 vermontc___7 Manchester<br>8 vermontc___8 Middlebury<br>9 vermontc___9 Montpelier<br>10 vermontc___10 Newport<br>11 vermontc___11 Plymouth<br>12 vermontc___12 Rutland<br>13 vermontc___13 Saint Albans<br>14 vermontc___14 Saint Johnsbury<br>15 vermontc___15 Sharon<br>16 vermontc___16 Winooski<br>17 vermontc___17 Not listed<br><br>Custom alignment: LH

<table>
<tr>
<td></td>
<td>139</td>
<td>virginiac

Show the field ONLY if:
[states(46)] = '1'</td>
<td>Which cities in Virginia</td>
<td>checkbox
<table>
<tr><td>1</td><td>virginiac___1</td><td>Abingdon</td></tr>
<tr><td>2</td><td>virginiac___2</td><td>Alexandria</td></tr>
<tr><td>3</td><td>virginiac___3</td><td>Bristol</td></tr>
<tr><td>4</td><td>virginiac___4</td><td>Charlottesville</td></tr>
<tr><td>5</td><td>virginiac___5</td><td>Chesapeake</td></tr>
<tr><td>6</td><td>virginiac___6</td><td>Danville</td></tr>
<tr><td>7</td><td>virginiac___7</td><td>Fairfax</td></tr>
<tr><td>8</td><td>virginiac___8</td><td>Falls Church</td></tr>
<tr><td>9</td><td>virginiac___9</td><td>Fredericksburg</td></tr>
<tr><td>10</td><td>virginiac___10</td><td>Hampton</td></tr>
<tr><td>11</td><td>virginiac___11</td><td>Hanover</td></tr>
<tr><td>12</td><td>virginiac___12</td><td>Hopewell</td></tr>
<tr><td>13</td><td>virginiac___13</td><td>Lexington</td></tr>
<tr><td>14</td><td>virginiac___14</td><td>Lynchburg</td></tr>
<tr><td>15</td><td>virginiac___15</td><td>Manassas</td></tr>
<tr><td>16</td><td>virginiac___16</td><td>Martinsville</td></tr>
<tr><td>17</td><td>virginiac___17</td><td>New Market</td></tr>
<tr><td>18</td><td>virginiac___18</td><td>Newport News</td></tr>
<tr><td>19</td><td>virginiac___19</td><td>Norfolk</td></tr>
<tr><td>20</td><td>virginiac___20</td><td>Petersburg</td></tr>
<tr><td>21</td><td>virginiac___21</td><td>Portsmouth</td></tr>
<tr><td>22</td><td>virginiac___22</td><td>Reston</td></tr>
<tr><td>23</td><td>virginiac___23</td><td>Richmond</td></tr>
<tr><td>24</td><td>virginiac___24</td><td>Roanoke</td></tr>
<tr><td>25</td><td>virginiac___25</td><td>Staunton</td></tr>
<tr><td>26</td><td>virginiac___26</td><td>Suffolk</td></tr>
<tr><td>27</td><td>virginiac___27</td><td>Virginia Beach</td></tr>
<tr><td>28</td><td>virginiac___28</td><td>Waynesboro</td></tr>
<tr><td>29</td><td>virginiac___29</td><td>Williamsburg</td></tr>
<tr><td>30</td><td>virginiac___30</td><td>Winchester</td></tr>
<tr><td>31</td><td>virginiac___31</td><td>Not listed</td></tr>
</table>
Custom alignment: LH</td>
</tr>
</table>

	140	washingtonc<br><br>Show the field ONLY if:<br>[states(47)] = '1'	Which cities in Washington?	checkbox<br>1 washingtonc___1 Aberdeen<br>2 washingtonc___2 Anacortes<br>3 washingtonc___3 Auburn<br>4 washingtonc___4 Bellevue<br>5 washingtonc___5 Bellingham<br>6 washingtonc___6 Bremerton<br>7 washingtonc___7 Centralia<br>8 washingtonc___8 Coulee Dam<br>9 washingtonc___9 Coupeville<br>10 washingtonc___10 Ellensburg<br>11 washingtonc___11 Ephrata<br>12 washingtonc___12 Everett<br>13 washingtonc___13 Hoquiam<br>14 washingtonc___14 Kelso<br>15 washingtonc___15 Kennewick<br>16 washingtonc___16 Longview<br>17 washingtonc___17 Moses Lake<br>18 washingtonc___18 Oak Harbor<br>19 washingtonc___19 Olympia<br>20 washingtonc___20 Pasco<br>21 washingtonc___21 Point Roberts<br>22 washingtonc___22 Port Angeles<br>23 washingtonc___23 Pullman<br>24 washingtonc___24 Puyallup<br>25 washingtonc___25 Redmond<br>26 washingtonc___26 Renton<br>27 washingtonc___27 Richland<br>28 washingtonc___28 Seattle<br>29 washingtonc___29 Spokane<br>30 washingtonc___30 Tacoma<br>31 washingtonc___31 Vancouver<br>32 washingtonc___32 Walla Walla<br>33 washingtonc___33 Wenatchee<br>34 washingtonc___34 Yakima<br>35 washingtonc___35 Not listed<br><br>Custom alignment: LH

	141	wvirginiac<br><br>Show the field ONLY if:<br>[states(48)] = '1'	Which cities in West Virginia?	checkbox<br>1 wvirginiac___1 Bath<br>2 wvirginiac___2 Beckley<br>3 wvirginiac___3 Bluefield<br>4 wvirginiac___4 Buckhannon<br>5 wvirginiac___5 Charles Town<br>6 wvirginiac___6 Charleston<br>7 wvirginiac___7 Clarksburg<br>8 wvirginiac___8 Elkins<br>9 wvirginiac___9 Fairmont<br>10 wvirginiac___10 Grafton<br>11 wvirginiac___11 Harpers Ferry<br>12 wvirginiac___12 Hillsboro<br>13 wvirginiac___13 Hinton<br>14 wvirginiac___14 Huntington<br>15 wvirginiac___15 Keyser<br>16 wvirginiac___16 Lewisburg<br>17 wvirginiac___17 Logan<br>18 wvirginiac___18 Martinsburg<br>19 wvirginiac___19 Morgantown<br>20 wvirginiac___20 Moundsville<br>21 wvirginiac___21 New Martinsville<br>22 wvirginiac___22 Parkersburg<br>23 wvirginiac___23 Philippi<br>24 wvirginiac___24 Point Pleasant<br>25 wvirginiac___25 Princeton<br>26 wvirginiac___26 Romney<br>27 wvirginiac___27 Shepherdstown<br>28 wvirginiac___28 South Charleston<br>29 wvirginiac___29 Summersville<br>30 wvirginiac___30 Weirton<br>31 wvirginiac___31 Welch<br>32 wvirginiac___32 Wellsburg<br>33 wvirginiac___33 Weston<br>34 wvirginiac___34 Wheeling<br>35 wvirginiac___35 White Sulphur Springs<br>36 wvirginiac___36 Williamson<br>37 wvirginiac___37 Not listed<br><br>Custom alignment: LH

	142	wisconsinc<br><br>Show the field ONLY if:<br>[states(49)] = '1'	Which cities in Wisconsin?	checkbox<br>1 wisconsinc___1 Appleton<br>2 wisconsinc___2 Ashland<br>3 wisconsinc___3 Baraboo<br>4 wisconsinc___4 Belmont<br>5 wisconsinc___5 Beloit<br>6 wisconsinc___6 Eau Claire<br>7 wisconsinc___7 Fond du Lac<br>8 wisconsinc___8 Green Bay<br>9 wisconsinc___9 Hayward<br>10 wisconsinc___10 Janesville<br>11 wisconsinc___11 Kenosha<br>12 wisconsinc___12 La Crosse<br>13 wisconsinc___13 Lake Geneva<br>14 wisconsinc___14 Madison<br>15 wisconsinc___15 Manitowoc<br>16 wisconsinc___16 Marinette<br>17 wisconsinc___17 Menasha<br>18 wisconsinc___18 Milwaukee<br>19 wisconsinc___19 Neenah<br>20 wisconsinc___20 New Glarus<br>21 wisconsinc___21 Oconto<br>22 wisconsinc___22 Oshkosh<br>23 wisconsinc___23 Peshtigo<br>24 wisconsinc___24 Portage<br>25 wisconsinc___25 Prairie du Chien<br>26 wisconsinc___26 Racine<br>27 wisconsinc___27 Rhinelander<br>28 wisconsinc___28 Ripon<br>29 wisconsinc___29 Sheboygan<br>30 wisconsinc___30 Spring Green<br>31 wisconsinc___31 Stevens Point<br>32 wisconsinc___32 Sturgeon Bay<br>33 wisconsinc___33 Superior<br>34 wisconsinc___34 Waukesha<br>35 wisconsinc___35 Wausau<br>36 wisconsinc___36 Wauwatosa<br>37 wisconsinc___37 West Allis<br>38 wisconsinc___38 West Bend<br>39 wisconsinc___39 Wisconsin Dells<br>40 wisconsinc___40 Not listed<br><br>Custom alignment: LH

<table>
<tr>
<td></td>
<td>143</td>
<td>wyomingc

Show the field ONLY if:
[states(50)] = '1'</td>
<td>Which cities in Wyoming?</td>
<td>checkbox
<table>
<tr><td>1</td><td>wyomingc___1</td><td>Buffalo</td></tr>
<tr><td>2</td><td>wyomingc___2</td><td>Casper</td></tr>
<tr><td>3</td><td>wyomingc___3</td><td>Cheyenne</td></tr>
<tr><td>4</td><td>wyomingc___4</td><td>Cody</td></tr>
<tr><td>5</td><td>wyomingc___5</td><td>Douglas</td></tr>
<tr><td>6</td><td>wyomingc___6</td><td>Evanston</td></tr>
<tr><td>7</td><td>wyomingc___7</td><td>Gillette</td></tr>
<tr><td>8</td><td>wyomingc___8</td><td>Green River</td></tr>
<tr><td>9</td><td>wyomingc___9</td><td>Jackson</td></tr>
<tr><td>10</td><td>wyomingc___10</td><td>Lander</td></tr>
<tr><td>11</td><td>wyomingc___11</td><td>Laramie</td></tr>
<tr><td>12</td><td>wyomingc___12</td><td>Newcastle</td></tr>
<tr><td>13</td><td>wyomingc___13</td><td>Powell</td></tr>
<tr><td>14</td><td>wyomingc___14</td><td>Rawlins</td></tr>
<tr><td>15</td><td>wyomingc___15</td><td>Riverton</td></tr>
<tr><td>16</td><td>wyomingc___16</td><td>Rock Springs</td></tr>
<tr><td>17</td><td>wyomingc___17</td><td>Sheridan</td></tr>
<tr><td>18</td><td>wyomingc___18</td><td>Ten Sleep</td></tr>
<tr><td>19</td><td>wyomingc___19</td><td>Thermopolis</td></tr>
<tr><td>20</td><td>wyomingc___20</td><td>Torrington</td></tr>
<tr><td>21</td><td>wyomingc___21</td><td>Worland</td></tr>
<tr><td>22</td><td>wyomingc___22</td><td>Not listed</td></tr>
</table>
Custom alignment: LH</td>
</tr>
<tr>
<td></td>
<td>144</td>
<td>nonuscountries

Show the field ONLY if:
[location(2)] = '1'</td>
<td>Which Non-US countries?</td>
<td>checkbox
<table>
<tr><td>1</td><td>nonuscountries___1</td><td>Afghanistan</td></tr>
<tr><td>2</td><td>nonuscountries___2</td><td>Albania</td></tr>
<tr><td>3</td><td>nonuscountries___3</td><td>Algeria</td></tr>
<tr><td>4</td><td>nonuscountries___4</td><td>Andorra</td></tr>
<tr><td>5</td><td>nonuscountries___5</td><td>Angola</td></tr>
<tr><td>6</td><td>nonuscountries___6</td><td>Antigua and Barbuda</td></tr>
<tr><td>7</td><td>nonuscountries___7</td><td>Argentina</td></tr>
<tr><td>8</td><td>nonuscountries___8</td><td>Armenia</td></tr>
<tr><td>9</td><td>nonuscountries___9</td><td>Australia</td></tr>
<tr><td>10</td><td>nonuscountries___10</td><td>Austria</td></tr>
<tr><td>11</td><td>nonuscountries___11</td><td>Azerbaijan</td></tr>
<tr><td>12</td><td>nonuscountries___12</td><td>Bahamas</td></tr>
<tr><td>13</td><td>nonuscountries___13</td><td>Bahrain</td></tr>
<tr><td>14</td><td>nonuscountries___14</td><td>Bangladesh</td></tr>
<tr><td>15</td><td>nonuscountries___15</td><td>Barbados</td></tr>
<tr><td>16</td><td>nonuscountries___16</td><td>Belarus</td></tr>
<tr><td>17</td><td>nonuscountries___17</td><td>Belgium</td></tr>
<tr><td>18</td><td>nonuscountries___18</td><td>Belize</td></tr>
<tr><td>19</td><td>nonuscountries___19</td><td>Benin</td></tr>
<tr><td>20</td><td>nonuscountries___20</td><td>Bhutan</td></tr>
<tr><td>21</td><td>nonuscountries___21</td><td>Bolivia</td></tr>
<tr><td>22</td><td>nonuscountries___22</td><td>Bosnia and Herzegovina</td></tr>
</table></td>
</tr>
</table>

23	nonuscountries___23	Botswana
24	nonuscountries___24	Brazil
25	nonuscountries___25	Brunei
26	nonuscountries___26	Bulgaria
27	nonuscountries___27	Burkina Faso
28	nonuscountries___28	Burundi
29	nonuscountries___29	Cabo Verde
30	nonuscountries___30	Cambodia
31	nonuscountries___31	Cameroon
32	nonuscountries___32	Canada
33	nonuscountries___33	Central African Republic (CAR)
34	nonuscountries___34	Chad
35	nonuscountries___35	Chile
36	nonuscountries___36	China
37	nonuscountries___37	Colombia
38	nonuscountries___38	Comoros
39	nonuscountries___39	Democratic Republic of the Congo
40	nonuscountries___40	Republic of the Congo
41	nonuscountries___41	Costa Rica
42	nonuscountries___42	Cote d'Ivoire
43	nonuscountries___43	Croatia
44	nonuscountries___44	Cuba
45	nonuscountries___45	Cyprus
46	nonuscountries___46	Czech Republic
47	nonuscountries___47	Denmark
48	nonuscountries___48	Djibouti
49	nonuscountries___49	Dominica
50	nonuscountries___50	Dominican Republic
51	nonuscountries___51	Ecuador
52	nonuscountries___52	Egypt
53	nonuscountries___53	El Salvador
54	nonuscountries___54	Equatorial Guinea
55	nonuscountries___55	Eritrea
56	nonuscountries___56	Estonia
57	nonuscountries___57	Eswatini (formerly Swaziland)
58	nonuscountries___58	Ethiopia
59	nonuscountries___59	Fiji
60	nonuscountries___60	Finland
61	nonuscountries___61	France
62	nonuscountries___62	Gabon
63	nonuscountries___63	Gambia
64	nonuscountries___64	Georgia
65	nonuscountries___65	Germany
66	nonuscountries___66	Ghana
67	nonuscountries___67	Greece

68	nonuscountries___68	Grenada
69	nonuscountries___69	Guatemala
70	nonuscountries___70	Guinea
71	nonuscountries___71	Guinea-Bissau
72	nonuscountries___72	Guyana
73	nonuscountries___73	Haiti
74	nonuscountries___74	Honduras
75	nonuscountries___75	Hungary
76	nonuscountries___76	Iceland
77	nonuscountries___77	India
78	nonuscountries___78	Indonesia
79	nonuscountries___79	Iran
80	nonuscountries___80	Iraq
81	nonuscountries___81	Ireland
82	nonuscountries___82	Israel
83	nonuscountries___83	Italy
84	nonuscountries___84	Jamaica
85	nonuscountries___85	Japan
86	nonuscountries___86	Jordan
87	nonuscountries___87	Kazakhstan
88	nonuscountries___88	Kenya
89	nonuscountries___89	Kiribati
90	nonuscountries___90	Kosovo
91	nonuscountries___91	Kuwait
92	nonuscountries___92	Kyrgyzstan
93	nonuscountries___93	Laos
94	nonuscountries___94	Latvia
95	nonuscountries___95	Lebanon
96	nonuscountries___96	Lesotho
97	nonuscountries___97	Liberia
98	nonuscountries___98	Libya
99	nonuscountries___99	Liechtenstein
100	nonuscountries___100	Lithuania
101	nonuscountries___101	Luxembourg
102	nonuscountries___102	Macedonia (FYROM)
103	nonuscountries___103	Madagascar
104	nonuscountries___104	Malawi
105	nonuscountries___105	Malaysia
106	nonuscountries___106	Maldives
107	nonuscountries___107	Mali
108	nonuscountries___108	Malta
109	nonuscountries___109	Marshall Islands
110	nonuscountries___110	Mauritania
111	nonuscountries___111	Mauritius
112	nonuscountries___112	Mexico
113	nonuscountries___113	Micronesia
114	nonuscountries___114	Moldova
115	nonuscountries___115	Monaco

116	nonuscountries___116	Mongolia
117	nonuscountries___117	Montenegro
118	nonuscountries___118	Morocco
119	nonuscountries___119	Mozambique
120	nonuscountries___120	Myanmar (formerly Burma)
121	nonuscountries___121	Namibia
122	nonuscountries___122	Nauru
123	nonuscountries___123	Nepal
124	nonuscountries___124	Netherlands
125	nonuscountries___125	New Zealand
126	nonuscountries___126	Nicaragua
127	nonuscountries___127	Niger
128	nonuscountries___128	Nigeria
129	nonuscountries___129	North Korea
130	nonuscountries___130	Norway
131	nonuscountries___131	Oman
132	nonuscountries___132	Pakistan
133	nonuscountries___133	Palau
134	nonuscountries___134	Palestine
135	nonuscountries___135	Panama
136	nonuscountries___136	Papua New Guinea
137	nonuscountries___137	Paraguay
138	nonuscountries___138	Peru
139	nonuscountries___139	Philippines
140	nonuscountries___140	Poland
141	nonuscountries___141	Portugal
142	nonuscountries___142	Qatar
143	nonuscountries___143	Romania
144	nonuscountries___144	Russia
145	nonuscountries___145	Rwanda
146	nonuscountries___146	Saint Kitts and Nevis
147	nonuscountries___147	Saint Lucia
148	nonuscountries___148	Saint Vincent and the Grenadines
149	nonuscountries___149	Samoa
150	nonuscountries___150	San Marino
151	nonuscountries___151	Sao Tome and Principe
152	nonuscountries___152	Saudi Arabia
153	nonuscountries___153	Senegal
154	nonuscountries___154	Serbia
155	nonuscountries___155	Seychelles
156	nonuscountries___156	Sierra Leone
157	nonuscountries___157	Singapore
158	nonuscountries___158	Slovakia
159	nonuscountries___159	Slovenia
160	nonuscountries___160	Solomon Islands
161	nonuscountries___161	Somalia

162	nonuscountries___162	South Africa
163	nonuscountries___163	South Korea
164	nonuscountries___164	South Sudan
165	nonuscountries___165	Spain
166	nonuscountries___166	Sri Lanka
167	nonuscountries___167	Sudan
168	nonuscountries___168	Suriname
169	nonuscountries___169	Swaziland (renamed to Eswatini)
170	nonuscountries___170	Sweden
171	nonuscountries___171	Switzerland
172	nonuscountries___172	Syria
173	nonuscountries___173	Taiwan
174	nonuscountries___174	Tajikistan
175	nonuscountries___175	Tanzania
176	nonuscountries___176	Thailand
177	nonuscountries___177	Timor-Leste
178	nonuscountries___178	Togo
179	nonuscountries___179	Tonga
180	nonuscountries___180	Trinidad and Tobago
181	nonuscountries___181	Tunisia
182	nonuscountries___182	Turkey
183	nonuscountries___183	Turkmenistan
184	nonuscountries___184	Tuvalu
185	nonuscountries___185	Uganda
186	nonuscountries___186	Ukraine
187	nonuscountries___187	United Arab Emirates (UAE)
188	nonuscountries___188	United Kingdom (UK)
189	nonuscountries___189	Uruguay
190	nonuscountries___190	Uzbekistan
191	nonuscountries___191	Vanuatu
192	nonuscountries___192	Vatican City (Holy See)
193	nonuscountries___193	Venezuela
194	nonuscountries___194	Vietnam
195	nonuscountries___195	Yemen
196	nonuscountries___196	Zambia
197	nonuscountries___197	Zimbabwe

Custom alignment: LH

	145	nonuscities<br><br>Show the field ONLY if: [nonuscountries(1)] = '1' or [nonuscountries(2)] = '1' or [nonuscountries(3)] = '1' or [nonuscountries(4)] = '1' or [nonuscountries(5)] = '1' or [nonuscountries(6)] = '1' or [nonuscountries(7)] = '1' or [nonuscountries(8)] = '1' or [nonuscountries(9)] = '1' or [nonuscountries(10)] = '1' or [nonuscountries	Which Cities? (if applicable)	notes

		(11)] = '1' or [nonuscountries<br>(13)] = '1' or [nonuscountries<br>(14)] = '1' or [nonuscountries<br>(15)] = '1' or [nonuscountries<br>(16)] = '1' or [nonuscountries<br>(18)] = '1' or [nonuscountries<br>(19)] = '1' or [nonuscountries<br>(20)] = '1' or [nonuscountries<br>(21)] = '1' or [nonuscountries<br>(22)] = '1' or [nonuscountries<br>(23)] = '1' or [nonuscountries<br>(24)] = '1' or [nonuscountries<br>(25)] = '1' or [nonuscountries<br>(26)] = '1' or [nonuscountries<br>(27)] = '1' or [nonuscountries<br>(28)] = '1' or [nonuscountries<br>(29)] = '1' or [nonuscountries<br>(30)] = '1' or [nonuscountries<br>(31)] = '1' or [nonuscountries<br>(32)] = '1' or [nonuscountries<br>(33)] = '1' or [nonuscountries<br>(34)] = '1' or [nonuscountries<br>(35)] = '1' or [nonuscountries<br>(38)] = '1' or [nonuscountries<br>(39)] = '1' or [nonuscountries<br>(36)] = '1' or [nonuscountries<br>(37)] = '1' or [nonuscountries<br>(40)] = '1' or [nonuscountries<br>(41)] = '1' or [nonuscountries<br>(46)] = '1' or [nonuscountries<br>(47)] = '1' or [nonuscountries<br>(48)] = '1' or [nonuscountries<br>(49)] = '1' or [nonuscountries<br>(50)] = '1' or [nonuscountries<br>(51)] = '1' or [nonuscountries<br>(52)] = '1' or [nonuscountries<br>(53)] = '1' or [nonuscountries<br>(54)] = '1' or [nonuscountries<br>(55)] = '1' or [nonuscountries<br>(56)] = '1' or [nonuscountries<br>(57)] = '1' or [nonuscountries<br>(58)] = '1' or [nonuscountries<br>(59)] = '1' or [nonuscountries<br>(60)] = '1' or [nonuscountries<br>(61)] = '1' or [nonuscountries<br>(62)] = '1' or [nonuscountries<br>(63)] = '1' or [nonuscountries<br>(64)] = '1' or [nonuscountries<br>(65)] = '1' or [nonuscountries<br>(66)] = '1' or [nonuscountries<br>(67)] = '1' or [nonuscountries<br>(68)] = '1' or [nonuscountries<br>(69)] = '1' or [nonuscountries<br>(70)] = '1' or [nonuscountries<br>(71)] = '1' or [nonuscountries<br>(72)] = '1' or [nonuscountries<br>(73)] = '1' or [nonuscountries<br>(74)] = '1' or [nonuscountries<br>(75)] = '1' or [nonuscountries<br>(76)] = '1' or [nonuscountries<br>(77)] = '1' or [nonuscountries<br>(78)] = '1' or [nonuscountries<br>(79)] = '1' or [nonuscountries<br>(80)] = '1' or [nonuscountries<br>(81)] = '1' or [nonuscountries<br>(82)] = '1' or [nonuscountries<br>(83)] = '1' or [nonuscountries<br>(84)] = '1' or [nonuscountries<br>(85)] = '1' or [nonuscountries<br>(86)] = '1' or [nonuscountries<br>(87)] = '1' or [nonuscountries<br>(88)] = '1' or [nonuscountries		

		(89)] = '1' or [nonuscountries<br>(90)] = '1' or [nonuscountries<br>(91)] = '1' or [nonuscountries<br>(92)] = '1' or [nonuscountries<br>(93)] = '1' or [nonuscountries<br>(94)] = '1' or [nonuscountries<br>(95)] = '1' or [nonuscountries<br>(96)] = '1' or [nonuscountries<br>(97)] = '1' or [nonuscountries<br>(98)] = '1' or [nonuscountries<br>(99)] = '1' or [nonuscountries<br>(102)] = '1' or [nonuscountries<br>(103)] = '1' or [nonuscountries<br>(104)] = '1' or [nonuscountries<br>(101)] = '1' or [nonuscountries<br>(100)] = '1' or [nonuscountries<br>(105)] = '1' or [nonuscountries<br>(106)] = '1' or [nonuscountries<br>(107)] = '1' or [nonuscountries<br>(108)] = '1' or [nonuscountries<br>(109)] = '1' or [nonuscountries<br>(110)] = '1' or [nonuscountries<br>(111)] = '1' or [nonuscountries<br>(112)] = '1' or [nonuscountries<br>(113)] = '1' or [nonuscountries<br>(114)] = '1' or [nonuscountries<br>(115)] = '1' or [nonuscountries<br>(116)] = '1' or [nonuscountries<br>(117)] = '1' or [nonuscountries<br>(118)] = '1' or [nonuscountries<br>(119)] = '1' or [nonuscountries<br>(120)] = '1' or [nonuscountries<br>(121)] = '1' or [nonuscountries<br>(122)] = '1' or [nonuscountries<br>(123)] = '1' or [nonuscountries<br>(124)] = '1' or [nonuscountries<br>(125)] = '1' or [nonuscountries<br>(126)] = '1' or [nonuscountries<br>(127)] = '1' or [nonuscountries<br>(128)] = '1' or [nonuscountries<br>(129)] = '1' or [nonuscountries<br>(130)] = '1' or [nonuscountries<br>(131)] = '1' or [nonuscountries<br>(132)] = '1' or [nonuscountries<br>(133)] = '1' or [nonuscountries<br>(134)] = '1' or [nonuscountries<br>(135)] = '1' or [nonuscountries<br>(136)] = '1' or [nonuscountries<br>(137)] = '1' or [nonuscountries<br>(138)] = '1' or [nonuscountries<br>(139)] = '1' or [nonuscountries<br>(140)] = '1' or [nonuscountries<br>(142)] = '1' or [nonuscountries<br>(141)] = '1' or [nonuscountries<br>(143)] = '1' or [nonuscountries<br>(144)] = '1' or [nonuscountries<br>(145)] = '1' or [nonuscountries<br>(146)] = '1' or [nonuscountries<br>(147)] = '1' or [nonuscountries<br>(148)] = '1' or [nonuscountries<br>(149)] = '1' or [nonuscountries<br>(150)] = '1' or [nonuscountries<br>(151)] = '1' or [nonuscountries<br>(153)] = '1' or [nonuscountries<br>(152)] = '1' or [nonuscountries<br>(154)] = '1' or [nonuscountries<br>(155)] = '1' or [nonuscountries<br>(156)] = '1' or [nonuscountries<br>(157)] = '1' or [nonuscountries<br>(158)] = '1' or [nonuscountries<br>(159)] = '1' or [nonuscountries<br>(160)] = '1' or [nonuscountries		

		(161)] = '1' or [nonuscountries<br>(162)] = '1' or [nonuscountries<br>(163)] = '1' or [nonuscountries<br>(164)] = '1' or [nonuscountries<br>(165)] = '1' or [nonuscountries<br>(166)] = '1' or [nonuscountries<br>(167)] = '1' or [nonuscountries<br>(168)] = '1' or [nonuscountries<br>(169)] = '1' or [nonuscountries<br>(170)] = '1' or [nonuscountries<br>(171)] = '1' or [nonuscountries<br>(172)] = '1' or [nonuscountries<br>(173)] = '1' or [nonuscountries<br>(174)] = '1' or [nonuscountries<br>(175)] = '1' or [nonuscountries<br>(177)] = '1' or [nonuscountries<br>(176)] = '1' or [nonuscountries<br>(178)] = '1' or [nonuscountries<br>(179)] = '1' or [nonuscountries<br>(180)] = '1' or [nonuscountries<br>(181)] = '1' or [nonuscountries<br>(182)] = '1' or [nonuscountries<br>(183)] = '1' or [nonuscountries<br>(184)] = '1' or [nonuscountries<br>(185)] = '1' or [nonuscountries<br>(186)] = '1' or [nonuscountries<br>(187)] = '1' or [nonuscountries<br>(188)] = '1' or [nonuscountries<br>(189)] = '1' or [nonuscountries<br>(190)] = '1' or [nonuscountries<br>(191)] = '1' or [nonuscountries<br>(192)] = '1' or [nonuscountries<br>(193)] = '1' or [nonuscountries<br>(194)] = '1' or [nonuscountries<br>(195)] = '1' or [nonuscountries<br>(196)] = '1' or [nonuscountries<br>(197)] = '1'		
	146	outcometype	Outcome Type	checkbox<br>1 | outcometype___1 | Incidence<br>2 | outcometype___2 | Mortality<br>3 | outcometype___3 | Other (Notes)
	147	other_outcometype<br>Show the field ONLY if:<br>[outcometype(3)]	Other type of outcome	notes
	148	analysistype	Analysis type	checkbox<br>1 | analysistype___1 | Case Control/ Odds Ratio<br>2 | analysistype___2 | Standardized Incidence Ratio<br>3 | analysistype___3 | Standardized Mortality Ratio<br>4 | analysistype___4 | Proportional Mortality Ratio<br>5 | analysistype___5 | Frequency<br>6 | analysistype___6 | Other (Notes)
	149	other_analysistype<br>Show the field ONLY if:<br>[analysistype(6)] = '1'	Other type of analysis	notes
	150	n	Total sample size	text
	151	npersonyrs	Total Person-Years	text
	152	sirn<br>Show the field ONLY if:<br>[analysistype(2)] = '1'	SIR n=	text

	153	freqposo_posf<br><br>Show the field ONLY if: [analysistype(5)] = '1' or [anal ysistype(1)] = '1'	n=<br>F+/O+<br>*number of firefighters with outcome*	text
	154	freq_nego_posf<br><br>Show the field ONLY if: [analysistype(5)] = '1' or [anal ysistype(1)] = '1'	n=<br>F+/O-<br>*number of firefighters without outcome*	text
	155	freq_poso_negf<br><br>Show the field ONLY if: [analysistype(5)] = '1' or [anal ysistype(1)] = '1'	n=<br>F-/O+<br>*number of non-firefighters with outcome*	text
	156	freq_nego_negf<br><br>Show the field ONLY if: [analysistype(5)] = '1' or [anal ysistype(1)] = '1'	n=<br>F-/O-<br>*number of non-firefighters without outcome*	text
	157	designation	Source of occupation designation	checkbox<br>1 | designation___1 | Employment records<br>2 | designation___2 | Certification records<br>3 | designation___3 | Cancer registry records<br>4 | designation___4 | Death certificate records<br>5 | designation___5 | Other (Notes)<br>6 | designation___6 | Not Reported
	158	otherdesignation<br><br>Show the field ONLY if: [designation(5)] = '1'	Other (Notes)	notes
	159	cancercoding	Cancer DX coding system	checkbox<br>1 | cancercoding___1 | ICD-O<br>2 | cancercoding___2 | ICD-O-2<br>3 | cancercoding___3 | ICD-O-3<br>4 | cancercoding___4 | ICD-8<br>5 | cancercoding___5 | ICD-9<br>6 | cancercoding___6 | ICD-10<br>7 | cancercoding___7 | SEER Codes<br>8 | cancercoding___8 | Other (Notes)<br>9 | cancercoding___9 | Not specified<br><br>Custom alignment: LH
	160	icd_9_code	ICD-9 code/recode	notes
	161	cancercodingpg<br><br>Show the field ONLY if: [cancercoding(1)] = '1' or [can cercoding(2)] = '1' or [cancerc oding(3)] = '1' or [cancercodin g(4)] = '1' or [cancercoding(4)] = '1' or [cancercoding(5)] = '1' or [cancercoding(6)] = '1' or [c ancercoding(7)] = '1' or [cance rcoding(8)] = '1'	Please provide the page number of the article with the codes on it.	notes<br>Custom alignment: LH
	162	cancercodingother<br><br>Show the field ONLY if: [cancercoding(8)]='1'	Other (Notes)	notes<br>Custom alignment: LH

	163	cancertypes	Cancer Types	checkbox<br>1 | cancertypes___1 | All<br>2 | cancertypes___2 | Subtypes<br>3 | cancertypes___3 | Subtypes by histology
	164	subtypeslist	Subtypes of cancers	notes
	165	insitu	Insitu<br>*In situ: These are a type of abnormal cells that have not spread from their original position in the body and may or may not become cancerous. Most cancer registries do no collect in situ cases with the exception of a few sites. Normally we would exclude in situ cases in data analyses, but it would be good to note of other studies we look at do not exclude them.*	radio<br>1 | Included<br>0 | Excluded<br>2 | Not reported
	166	malignant	Malignant/ Invasive<br>*Non-in situ cases, i.e., true cancers.*	radio<br>1 | Included<br>0 | Excluded<br>2 | Not reported
	167	latestage	Cancer stage	radio<br>0 | Early stage<br>1 | Late stage<br>2 | Not reported
	168	agetiming	Age	checkbox<br>1 | agetiming___1 | At cohort start date<br>2 | agetiming___2 | At cohort end date<br>3 | agetiming___3 | At death<br>4 | agetiming___4 | At diagnosis<br>5 | agetiming___5 | At hire<br>6 | agetiming___6 | Not reported
	169	agecsm<br><br>Show the field ONLY if:<br>[agetiming(1)] = '1'	Age at cohort start date: Mean	text (number)
	170	agecssd<br><br>Show the field ONLY if:<br>[agetiming(1)] = '1'	Age at cohort start date : Standard Deviation<br>*If variance is given please convert to standard deviation*	text
	171	agecsmin<br><br>Show the field ONLY if:<br>[agetiming(1)] = '1'	Age at cohort start date: Min	text (number)
	172	agecsmax<br><br>Show the field ONLY if:<br>[agetiming(1)] = '1'	Age at cohort start date: Max	text
	173	agestartfreq<br><br>Show the field ONLY if:<br>[agetiming(1)] = '1'	Age at cohort start<br>Frequencies and percentages	notes
	174	agecem<br><br>Show the field ONLY if:<br>[agetiming(2)] = '1'	Age at cohort end date: Mean	text (number)
	175	agecesd<br><br>Show the field ONLY if:<br>[agetiming(2)] = '1'	Age at cohort end date : Standard Deviation<br>*If variance is given please convert to standard deviation*	text
	176	agecemin<br><br>Show the field ONLY if:<br>[agetiming(2)] = '1'	Age at cohort end date: Min	text (number)
	177	agecemax<br><br>Show the field ONLY if:<br>[agetiming(2)] = '1'	Age at cohort end date: Max	text

	178	ageendfreq<br><br>Show the field ONLY if:<br>[agetiming(2)] = '1'	Age at cohort end<br>Frequencies and percentages	notes
	179	agedeathm<br><br>Show the field ONLY if:<br>[agetiming(3)] = '1'	Age at death: Mean	text (number)
	180	agedeathsd<br><br>Show the field ONLY if:<br>[agetiming(3)] = '1'	Age at death : Standard Deviation<br>*If variance is given please convert to standard deviation*	text
	181	agedeathmin<br><br>Show the field ONLY if:<br>[agetiming(3)] = '1'	Age at death: Min	text (number)
	182	agedeathmax<br><br>Show the field ONLY if:<br>[agetiming(3)] = '1'	Age at death: Max	text
	183	agedeathfreq<br><br>Show the field ONLY if:<br>[agetiming(3)] = '1'	Age at death<br>Frequencies and percentages	notes
	184	agedxm<br><br>Show the field ONLY if:<br>[agetiming(4)] = '1'	Age at dx: Mean	text (number)
	185	agedxsd<br><br>Show the field ONLY if:<br>[agetiming(4)] = '1'	Age at dx : Standard Deviation<br>*If variance is given please convert to standard deviation*	text
	186	agedxmin<br><br>Show the field ONLY if:<br>[agetiming(4)] = '1'	Age at dx: Min	text (number)
	187	agedxmax<br><br>Show the field ONLY if:<br>[agetiming(4)] = '1'	Age at dx: Max	text
	188	agedxfreq<br><br>Show the field ONLY if:<br>[agetiming(4)] = '1'	Age at dx<br>Frequencies and percentages	notes
	189	agehirem<br><br>Show the field ONLY if:<br>[agetiming(5)]='1'	Age at hire: Mean	text
	190	agehiresd<br><br>Show the field ONLY if:<br>[agetiming(5)]='1'	Age at hire: SD<br>*if variance is reported please convert it to standard deviation and type here*	text
	191	agehiremin<br><br>Show the field ONLY if:<br>[agetiming(5)]='1'	Age at hire: Min	text
	192	agehiremax<br><br>Show the field ONLY if:<br>[agetiming(5)]='1'	Age at hire: Max	text
	193	agehirefreq<br><br>Show the field ONLY if:<br>[agetiming(5)] = '1'	Age at hire<br>Frequencies and percentages	notes
	194	employcontinuous	Is duration of employment in years collected categorically or continuously?	radio<br>0 Categorically<br>1 Continuous

	195	employmentyrsmean<br><br>Show the field ONLY if:<br>[employcontinuous] = '1'	Employment in years<br>Mean:	text (number)
	196	employmentyrssd<br><br>Show the field ONLY if:<br>[employcontinuous] = '1'	Employment in years<br>SD:<br>*(if variance is reported please convert it to standard deviation and type here)*	text (number)
	197	employmentyrsmin<br><br>Show the field ONLY if:<br>[employcontinuous] = '1'	Employment in years<br>Min:	text (number)
	198	employmentyrsmax<br><br>Show the field ONLY if:<br>[employcontinuous] = '1'	Employment in years<br>Max:	text (number)
	199	durationcat<br><br>Show the field ONLY if:<br>[employcontinuous] = '0'	Type in the categories best fitting for employment in years.	notes
	200	employstatus	Employment status	radio<br>1 Part-time<br>2 Full-time<br>3 Other (Notes)<br>4 Not reported
	201	otheremploystatus<br><br>Show the field ONLY if:<br>[employstatus] = '3'	Other employment status	notes
	202	incident_type	Type of incident attended	checkbox<br>1 incident_type___1 All fires<br>2 incident_type___2 Landscape fires<br>3 incident_type___3 Vehicle fires<br>4 incident_type___4 Structural<br>5 incident_type___5 Other (Notes)<br>6 incident_type___6 Not specified
	203	otherincident_type<br><br>Show the field ONLY if:<br>[incident_type(5)] = '1'	Other incident type	notes
	204	eratype	Era of first employment<br>*Era of employment: When they were first certified or first started working as a firefighter so that we can accurately document how long they have been exposed.*	radio<br>1 Continuous<br>2 Categorical
	205	firstemploym<br><br>Show the field ONLY if:<br>[eratype] = '1'	Mean year of first employment	text (number)
	206	firstemploysd<br><br>Show the field ONLY if:<br>[eratype] = '1'	SD year of first employment<br>*(if variance is reported please convert it to standard deviation and type here)*	text
	207	firstemploymin<br><br>Show the field ONLY if:<br>[eratype]='1'	Minimum year of first employment	text
	208	firstemploymax<br><br>Show the field ONLY if:<br>[eratype]='1'	Maximum year of first employment	text
	209	firstemploycat<br><br>Show the field ONLY if:<br>[eratype]='2'	Type in the categories for Era of first employment	notes

<table>
<tr><td></td><td>210</td><td>gender</td><td>Gender</td><td>checkbox
<table><tr><td>1</td><td>gender___1</td><td>Male</td></tr><tr><td>2</td><td>gender___2</td><td>Female</td></tr><tr><td>3</td><td>gender___3</td><td>Other (Please specify)</td></tr><tr><td>4</td><td>gender___4</td><td>Not reported</td></tr></table></td></tr>
<tr><td></td><td>211</td><td>othergender

Show the field ONLY if:
[gender(3)] = '1'</td><td>Other</td><td>notes</td></tr>
<tr><td></td><td>212</td><td>malen

Show the field ONLY if:
[gender(1)]='1'</td><td>Male n=</td><td>text (number)</td></tr>
<tr><td></td><td>213</td><td>malepct

Show the field ONLY if:
[gender(1)]='1'</td><td>Male %=</td><td>text</td></tr>
<tr><td></td><td>214</td><td>femalen

Show the field ONLY if:
[gender(2)] = '1'</td><td>Female n=</td><td>text (number)</td></tr>
<tr><td></td><td>215</td><td>femalepct

Show the field ONLY if:
[gender(2)] = '1'</td><td>Female %=</td><td>text</td></tr>
<tr><td></td><td>216</td><td>othergendern

Show the field ONLY if:
[gender(3)] = '1'</td><td>Other n=</td><td>text (number)</td></tr>
<tr><td></td><td>217</td><td>othergenderpct

Show the field ONLY if:
[gender(3)]='1'</td><td>Other %=</td><td>text (number)</td></tr>
<tr><td></td><td>218</td><td>race</td><td>Race/ Ethnicity</td><td>checkbox
<table><tr><td>1</td><td>race___1</td><td>White</td></tr><tr><td>2</td><td>race___2</td><td>Black</td></tr><tr><td>3</td><td>race___3</td><td>Asian</td></tr><tr><td>4</td><td>race___4</td><td>Other</td></tr><tr><td>5</td><td>race___5</td><td>Unknown</td></tr><tr><td>6</td><td>race___6</td><td>Not reported</td></tr><tr><td>7</td><td>race___7</td><td>Notes</td></tr><tr><td>8</td><td>race___8</td><td>Hispanic</td></tr></table>
Custom alignment: LH</td></tr>
<tr><td></td><td>219</td><td>whiten

Show the field ONLY if:
[race(1)]='1'</td><td>White n=</td><td>text
Custom alignment: RH</td></tr>
<tr><td></td><td>220</td><td>whitepct

Show the field ONLY if:
[race(1)]='1'</td><td>White %=</td><td>text
Custom alignment: RH</td></tr>
<tr><td></td><td>221</td><td>blackn

Show the field ONLY if:
[race(2)] = '1'</td><td>Black n=</td><td>text
Custom alignment: RH</td></tr>
<tr><td></td><td>222</td><td>blackpct

Show the field ONLY if:
[race(2)] = '1'</td><td>Black %=</td><td>text
Custom alignment: RH</td></tr>
<tr><td></td><td>223</td><td>asiann

Show the field ONLY if:
[race(3)] = '1'</td><td>Asian n=</td><td>text
Custom alignment: RH</td></tr>
</table>

	224	asianpct<br><br>Show the field ONLY if:<br>[race(3)] = '1'	Asian %=	text<br>Custom alignment: RH
	225	otherrace<br><br>Show the field ONLY if:<br>[race(4)] = '1'	Other<br>*please list the other race*	text<br>Custom alignment: RH
	226	othern<br><br>Show the field ONLY if:<br>[race(4)] = '1'	other n=	text<br>Custom alignment: RH
	227	otherpct<br><br>Show the field ONLY if:<br>[race(4)] = '1'	other %=	text
	228	unknownn<br><br>Show the field ONLY if:<br>[race(5)] = '1'	Unknown n=	text (number)<br>Custom alignment: RH
	229	unknownpct<br><br>Show the field ONLY if:<br>[race(5)]='1'	Unknown %=	text (number)<br>Custom alignment: RH
	230	notes<br><br>Show the field ONLY if:<br>[race(7)] = '1'	Notes	notes
	231	hispanicn<br><br>Show the field ONLY if:<br>[race(8)]='1'	Hispanic n=	text (number)<br>Custom alignment: RH
	232	hispanicpct<br><br>Show the field ONLY if:<br>[race(8)]='1'	Hispanic %=	text<br>Custom alignment: RH
	233	mortalive<br><br>Show the field ONLY if:<br>[outcometype(2)] = '1'	Section Header: *Mortality follow-up*<br>Alive n=	text
	234	alivepct<br><br>Show the field ONLY if:<br>[outcometype(2)] = '1'	Alive %=	text (number, Max: 100)
	235	mortdead<br><br>Show the field ONLY if:<br>[outcometype(2)] = '1'	Dead n=	text
	236	deadpct<br><br>Show the field ONLY if:<br>[outcometype(2)] = '1'	Dead %=	text
	237	pyearsm<br><br>Show the field ONLY if:<br>[outcometype(2)] = '1'	Person years of follow up<br>Mean=	text
	238	pyearssd<br><br>Show the field ONLY if:<br>[outcometype(2)] = '1'	Person years of follow up<br>SD=	text
	239	pyearsmin<br><br>Show the field ONLY if:<br>[outcometype(2)] = '1'	Person years of follow up<br>Min=	text
	240	pyearsmax<br><br>Show the field ONLY if:<br>[outcometype(2)] = '1'	Person years of follow up<br>Max=	text

	241	lengthfum<br><br>Show the field ONLY if:<br>[outcometype(2)] = '1'	Average length of follow up in years<br>Mean=	text
	242	lengthfusd<br><br>Show the field ONLY if:<br>[outcometype(2)] = '1'	Length of follow-up in years<br>SD=	text
	243	death_id_agencyn<br><br>Show the field ONLY if:<br>[outcometype(2)] = '1'	Deaths notified by agencies<br>n=	text
	244	death_id_agencypct<br><br>Show the field ONLY if:<br>[outcometype(2)] = '1'	Deaths notified by agencies<br>%=	text
	245	linkagedeathsn<br><br>Show the field ONLY if:<br>[outcometype(2)] = '1'	Deaths from linkage<br>n=	text
	246	linkagedeathpct<br><br>Show the field ONLY if:<br>[outcometype(2)] = '1'	Deaths from linkage<br>%=	text
	247	ageatdeathm<br><br>Show the field ONLY if:<br>[outcometype(2)] = '1'	Age at death, if deceased<br>Mean=	text
	248	ageatdeathsd<br><br>Show the field ONLY if:<br>[outcometype(2)] = '1'	Age at death, if deceased<br>SD=	text
	249	pyearsincm<br><br>Show the field ONLY if:<br>[outcometype(1)] = '1'	Section Header: *Cancer Incidence- Follow up*<br><br>Person years of follow up<br>Mean=	text
	250	pyearsincsd<br><br>Show the field ONLY if:<br>[outcometype(1)] = '1'	Person years of follow up<br>SD=	text
	251	pyearsincmin<br><br>Show the field ONLY if:<br>[outcometype(1)] = '1'	Person years of follow up<br>Min=	text
	252	pyearsincmax<br><br>Show the field ONLY if:<br>[outcometype(1)] = '1'	Person years of follow up<br>Max=	text
	253	dxcancerm<br><br>Show the field ONLY if:<br>[outcometype(1)] = '1'	Age at diagnosis of first cancer<br>Mean=	text
	254	dxcancersd<br><br>Show the field ONLY if:<br>[outcometype(1)] = '1'	Age at diagnosis of first cancer<br>SD=	text
	255	dxcancermin<br><br>Show the field ONLY if:<br>[outcometype(1)] = '1'	Age at diagnosis of first cancer<br>Min=	text
	256	dxcancermax<br><br>Show the field ONLY if:<br>[outcometype(1)] = '1'	Age at diagnosis of first cancer<br>Max=	text
	257	linkagecancern<br><br>Show the field ONLY if:<br>[outcometype(1)] = '1'	Cancers from linkage<br>n=	text

	258	linkagecancerpct<br><br>Show the field ONLY if:<br>[outcometype(1)] = '1'	Cancers from linkage<br>%=	text
	259	firstprimary	First Primary Analysis<br>*First primaries are the first diagnosed cancer for a particular person and secondary primaries are any subsequent cancers diagnosed in the same person, but not including metastases.*	radio<br>1 Yes<br>2 No<br>3 Not applicable
	260	multipleprimary	Multiple Primary analysis	radio<br>1 Yes<br>2 No<br>3 Not applicable
	261	smoking	Section Header: *Smoking Information*<br><br>Smoking Status	radio<br>0 Never smoker<br>1 Former smoker<br>2 Current smoker<br>3 Ever smoker<br>4 Not reported
	262	cigpackyears<br><br>Show the field ONLY if:<br>[smoking] = '1' or [smoking] = '2' or [smoking] = '3'	Are the cigarette pack years collected continuously or categorically?	radio<br>1 Continuously<br>2 Categorically<br>3 Not reported
	263	cigpackyearsm<br><br>Show the field ONLY if:<br>[cigpackyears] = '1'	Cigarette pack years<br>Mean=	text
	264	cigpackyearssd<br><br>Show the field ONLY if:<br>[cigpackyears] = '1'	Cigarette pack years<br>SD=	text
	265	cigpackyearsmin<br><br>Show the field ONLY if:<br>[cigpackyears] = '1'	Cigarette pack years<br>Min=	text
	266	cigpackyearsmax<br><br>Show the field ONLY if:<br>[cigpackyears] = '1'	Cigarette pack years<br>Max=	text
	267	cigpackyearscat<br><br>Show the field ONLY if:<br>[cigpackyears]='2'	Cigarette pack years	radio<br>1 < 10 pack years<br>2 10-20 pack years<br>3 21+ pack years
	268	characteristics_complete	Section Header: *Form Status*<br><br>Complete?	dropdown<br>0 Incomplete<br>1 Unverified<br>2 Complete
Instrument: **Rejection Form** (rejection_form)				Collapse
	269	coder	Coder's name<br>(Please type your last name)<br>*please use only lowercase letters*	text

	#	Variable	Field Label	Field Type
	270	reject_si	Study Id:<br>Last name of first author (first letter - cap) + first letter (cap) of second author's last name + first letter (cap) of third author's last name (if only two authors exist, just use Last name of first author + first letter of second author's last name)_pubyear<br>Ahn, Y.S. & Jeong, K.S. 2015 = AhnJ2015<br>Ahn, Y.S., Jeong, K.S, & Kim, K. S. 2012 = AhnJK2012<br><br>if unpublished use 0000 as year	notes, Required
	271	reject	Study rejected?	yesno<br>1 Yes<br>0 No
	272	reason_reject<br>Show the field ONLY if:<br>[reject]="1"	Why was it rejected?	notes
	273	rejection_form_complete	Section Header: *Form Status*<br>Complete?	dropdown<br>0 Incomplete<br>1 Unverified<br>2 Complete
Instrument: **SIR Overall** (sir_overall)				Collapse
	274	siroverall_si	Study Id:<br>Last name of first author (first letter - cap) + first letter (cap) of second author's last name + first letter (cap) of third author's last name (if only two authors exist, just use Last name of first author + first letter of second author's last name)_pubyear<br>Ahn, Y.S. & Jeong, K.S. 2015 = AhnJ2015<br>Ahn, Y.S., Jeong, K.S, & Kim, K. S. 2012 = AhnJK2012<br><br>if unpublished use 0000 as year	text
	275	sir_coder	Coder's name<br>(Please type your last name)<br>*please use only lowercase letters*	text
	276	rejectsir	Study rejected?	yesno<br>1 Yes<br>0 No
	277	sampledessir	Description of the sample associated with the effect size.<br><br>Eg. n_female_age30_breast_norway<br>*please use only lowercase letters*	notes
	278	sirotheref	Notes on different effect sizes provided by study for future reference.	notes
	279	smrcontrolled	Variable controlled for:	notes
	280	sircomputed	How was the SIR computed?	notes
	281	sirtotaln	Total N	text (number)
	282	numerator	Numerator<br><br>eg. the numerator was the sum of the number of cancer cases	notes
	283	denominator	Denominator<br><br>e.g. multiplying the stratum specific rates in men by the corresponding number of person-years in the cohort and then adding over all strata	notes
	284	siroverallest	SIR: Overall<br>Estimate	text (number)

	285	siroverall_se	SIR: Overall<br>Standard error	text
	286	siroverall_ll	SIR : Overall<br>Confidence Interval LL	text (number)
	287	siroverall_ul	SIR: Overall<br>Confidence Interval UL	text (number)
	288	sir_overall_complete	Section Header: *Form Status*<br>Complete?	dropdown<br>0 Incomplete<br>1 Unverified<br>2 Complete
Instrument: **OR Overall** (or_overall)				Collapse
	289	oroverall_si	Study Id:<br>Last name of first author (first letter - cap) + first letter (cap) of second author's last name + first letter (cap) of third author's last name (if only two authors exist, just use Last name of first author + first letter of second author's last name)_pubyear<br>Ahn, Y.S. & Jeong, K.S. 2015 = AhnJ2015<br>Ahn, Y.S., Jeong, K.S, & Kim, K. S. 2012 = AhnJK2012<br><br>if unpublished use 0000 as year	text
	290	or_coder	Coder's name<br>(Please type your last name)<br>*please use only lowercase letters*	text
	291	rejector	Study rejected?	yesno<br>1 Yes<br>0 No
	292	sampledesor	Description of the sample associated with the effect size.<br><br>Eg. n_female_age30_breast_norway<br>*please use only lowercase letters*	notes
	293	orotheref	Notes on different effect sizes provided by study for future reference.	notes
	294	orcontrolled	Variable controlled for:	notes
	295	oroverallest	OR: Overall<br>Estimate	text (number)
	296	oroverall_se	OR: Overall<br>Standard error	text
	297	oroverall_ll	OR: Overall<br>Confidence Interval LL	text (number)
	298	oroverall_ul	OR: Overall<br>Confidence Interval UL	text (number)
	299	orfo	Number of firefighters with outcome<br>F+/O+	text
	300	orfno	Number of firefighter without outcome<br>F+/O-	text
	301	ornfo	Number of Non-firefighters with outcome<br>F-/O+	text
	302	ornfnd	Number of non-firefighters without outcome<br>F-/O-	text
	303	or_overall_complete	Section Header: *Form Status*<br>Complete?	dropdown<br>0 Incomplete<br>1 Unverified<br>2 Complete
Instrument: **SMR Overall** (smr_overall)				Collapse

	304	smroverall_si	Study Id:<br>Last name of first author (first letter - cap) + first letter (cap) of second author's last name + first letter (cap) of third author's last name (if only two authors exist, just use Last name of first author + first letter of second author's last name)_pubyear<br>Ahn, Y.S. & Jeong, K.S. 2015 = AhnJ2015<br>Ahn, Y.S., Jeong, K.S, & Kim, K. S. 2012 = AhnJK2012<br><br>if unpublished use 0000 as year	text
	305	smr_coder	Coder's name<br>(Please type your last name)<br>*please use only lowercase letters*	text
	306	rejectsmr	Study rejected?	yesno<br>1 Yes<br>0 No
	307	sampledessmr	Description of the sample associated with the effect size.<br><br>Eg. female_age30_breast_norway<br>*please use only lowercase letters*	notes
	308	smrotheref	Notes on different effect sizes provided by study for future reference.	notes
	309	smres	How was the effect size calculated?	notes
	310	smrtotaln	SMR Total N	text (number)
	311	smrnumerator	Numerator:<br>Observed deaths	text
	312	smrdenom	Denominator<br>Expected deaths	text
	313	smroverallest	SMR: Overall<br>Estimate	text (number)
	314	smroverall_se	SMR: Overall<br>Standard error	text
	315	smroverall_ll	SMR : Overall<br>Confidence Interval LL	text (number)
	316	smroverall_ul	SMR: Overall<br>Confidence Interval UL	text (number)
	317	smr_overall_complete	Section Header: *Form Status*<br>Complete?	dropdown<br>0 Incomplete<br>1 Unverified<br>2 Complete

Instrument: **PMR Overall** (pmr_overall) Collapse

	318	pmroverall_si	Study Id:<br>Last name of first author (first letter - cap) + first letter (cap) of second author's last name + first letter (cap) of third author's last name (if only two authors exist, just use Last name of first author + first letter of second author's last name)_pubyear<br>Ahn, Y.S. & Jeong, K.S. 2015 = AhnJ2015<br>Ahn, Y.S., Jeong, K.S, & Kim, K. S. 2012 = AhnJK2012<br><br>if unpublished use 0000 as year	text
	319	pmr_coder	Coder's name<br>(Please type your last name)<br>*please use only lowercase letters*	text
	320	rejectpmr	Study rejected?	yesno<br>1 Yes<br>0 No

	321	sampledespmr	Description of the sample associated with the effect size.<br><br>Eg. female_age30_breast_norway<br>*please use only lowercase letters*	notes
	322	pmrotheref	Notes on different effect sizes provided by study for future reference.	notes
	323	pmrcontrolled	Variable controlled for:	notes
	324	pmres	How did they calculate the effect size?	notes
	325	pmrtotaln	PMR Total N	text (number)
	326	pmrnumerator	Numerator<br>Number of death from a specific cause	text
	327	pmrdenom	Denominator<br>Number of death in the population	text
	328	pmroverallest	PMR: Overall<br>Estimate	text (number)
	329	pmroverall_se	PMR: Overall<br>Standard error	text
	330	pmroverall_ll	PMR : Overall<br>Confidence Interval LL	text (number)
	331	pmroverall_ul	PMR: Overall<br>Confidence Interval UL	text (number)
	332	pmr_overall_complete	Section Header: *Form Status*<br>Complete?	dropdown<br>0 Incomplete<br>1 Unverified<br>2 Complete

Instrument: **Frequency** (frequency) Collapse

	333	freqoverall_si	Study Id:<br>Last name of first author (first letter - cap) + first letter (cap) of second author's last name + first letter (cap) of third author's last name (if only two authors exist, just use Last name of first author + first letter of second author's last name)_pubyear<br>Ahn, Y.S. & Jeong, K.S. 2015 = AhnJ2015<br>Ahn, Y.S., Jeong, K.S, & Kim, K. S. 2012 = AhnJK2012<br><br>if unpublished use 0000 as year	text
	334	freq_coder	Coder's name<br>(Please type your last name)<br>*please use only lowercase letters*	text
	335	rejectfreq	Study rejected?	yesno<br>1 Yes<br>0 No
	336	freqfo	Number of firefighters with outcome<br>F+/O+	text
	337	freqfneg0	Number of firefighter without outcome<br>F+/O-	text
	338	freqnegfo	Number of Non-firefighters with outcome<br>F-/O+	text
	339	freqnegfnego	Number of non-firefighters without outcome<br>F-/O-	text
	340	frequency_complete	Section Header: *Form Status*<br>Complete?	dropdown<br>0 Incomplete<br>1 Unverified<br>2 Complete

Instrument: **Other Effect Size** (other_effect_size) Collapse

	341	othereffectsize_si	Study Id:<br>Last name of first author (first letter - cap) + first letter (cap) of second author's last name + first letter (cap) of third author's last name (if only two authors exist, just use Last name of first author + first letter of second author's last name)_pubyear<br>Ahn, Y.S. & Jeong, K.S. 2015 = AhnJ2015<br>Ahn, Y.S., Jeong, K.S, & Kim, K. S. 2012 = AhnJK2012<br><br>if unpublished use 0000 as year	text
	342	othereffectsize_coder	Coder's name<br>(Please type your last name)<br>*please use only lowercase letters*	text
	343	othereffectsize	Effect size type<br><br>(Eg. Hazard ratios related incidence)	notes
	344	otherespg	Page numbers	notes
	345	other_effect_size_complete	Section Header: *Form Status*<br>Complete?	dropdown<br>0 Incomplete<br>1 Unverified<br>2 Complete
Instrument: **Quality Scale Case-Control** (quality_scale_casecontrol)				Collapse
	346	qualityscale	The Newcastle-Ottawa Quality Assessment Scale<br>Case Control Studies<br><br>Note: A study can be awarded a maximum of one star for each numbered item within the Selection and Exposure categories. A maximum of two stars can be given for Comparability.	descriptive
	347	nos_ca_co_codername	Coder's last name	text
	348	selection1_cc	Section Header: *SELECTION*<br>1. Is the Case Definition Adequate? a) yes, with independent validation (Requires some independent validation (e.g. >1 person/record/time/process to extract information, or reference to primary record source such as x-rays or medical/hospital records)) =1 star b) yes, eg record linkage or based on self reports (Record linkage (e.g. ICD codes in database) or self-report with no reference to primary record) =0 stars c) No description =0 stars Note: ICD coding version should be specified in order to be awarded one star	dropdown<br>0 0 stars<br>1 1 star<br><br>Custom alignment: LV
	349	selection2_cc	2. Representativeness of the Cases<br><br>a) consecutive or obviously representative series of cases (All eligible cases with outcome of interest over a defined period of time, all cases in a defined hospital or clinic, group of hospitals, health maintenance organisation, or an appropriate sample of those cases (e.g. random sample)) =1 star<br><br>b) potential for selection biases or not stated (Not satisfying requirements in part (a), or not stated.) =0 stars	dropdown<br>0 0 stars<br>1 1 star<br><br>Custom alignment: LV

	350	selection3_cc	3. Selection of Controls<br><br>This item assesses whether the control series used in the study is derived from the same population as the cases and essentially would have been cases had the outcome been present.<br><br>a) community controls (i.e. same community as cases and would be cases if had outcome)) =1 star<br><br>b) hospital controls (within same community as cases (i.e. not another city) but derived from a hospitalised population) =0 stars<br><br>c) No description =0 stars	dropdown<br>0 | 0 stars<br>1 | 1 star<br><br>Custom alignment: LV
	351	selection4_cc	4) Definition of Controls<br><br>a) no history of diesease (endpoint) (If cases are first occurrence of outcome, then it must explicitly state that controls have no history of this outcome. If cases have new (not necessarily first) occurrence of outcome, then controls with previous occurrences of outcome of interest should not be excluded.) =1 star<br><br>b) no description of source (No mention of history of outcome) =0 stars	dropdown<br>0 | 0 stars<br>1 | 1 star<br><br>Custom alignment: LV
	352	compare_cc	Section Header: *COMPARABILITY A maximum of 2 stars can be allotted in this category Either cases and controls must be matched in the design and/or confounders must be adjusted for in the analysis. Statements of no differences between groups or that differences were not statistically significant are not sufficient for establishing comparability. Note: If the odds ratio for the exposure of interest is adjusted for the confounders listed, then the groups will be considered to be comparable on each variable used in the adjustment. There may be multiple ratings for this item for different categories of exposure (e.g. ever vs. never, current vs. previous or never) Age =*, Other controlled factors =**<br><br>1) Comparability of Cases and Controls on the Basis of the Design or Analysis a) study controls for _______________ (Select the most important factor.) =1 star b) study controls for any additional factor (This criteria could be modified to indicate specific control for a second important factor.) =1 star Note: age only =1, age+any other variable=2	dropdown<br>0 | 0 stars<br>1 | 1 star<br>2 | 2 stars<br><br>Custom alignment: LV
	353	exp1_cc	Section Header: *EXPOSURE*<br><br>1) Ascertainment of exposure a) secure record (eg surgical records)=1 star b) structured interview where blind to case/control status =1 star c) interview not blinded to case/control status=0 stars d) written self report or medical record only=0 stars e) no description=0 stars Note: Can ignore "blind to case/control status" and code all structured interviews as=1.	dropdown<br>0 | 0 stars<br>1 | 1 star<br><br>Custom alignment: LV
	354	exp2_cc	2) Same method of ascertainment for cases and controls a) yes=1 star b) no=0 stars Note: In the case of occupational/certification determination of occupational status applied only to firefighters then code=0.	dropdown<br>0 | 0 stars<br>1 | 1 star<br><br>Custom alignment: LV
	355	exp3_cc	(DO NOT CODE THIS ITEM) 3) Non-Response rate a) same rate for both groups=1 star b) non respondents described=0 stars c) rate different and no designation=0 stars	dropdown<br>0 | 0 stars<br>1 | 1 star<br><br>Custom alignment: LV
	356	star_total_cc	Total # of stars	calc<br>Calculation: ([selection1_cc]+ [selection2_cc]+ [selection3_cc]+ [selection4_cc]+ [compare_cc]+ [exp1_cc]+[exp2_cc]+[exp3_cc])<br>Custom alignment: LV

	357	support_cc	Were any supporting documents referred to in filling out information for this form?	yesno<br>1 Yes<br>0 No<br><br>Custom alignment: LV
	358	pdf1_cc<br><br>Show the field ONLY if:<br>[support_cc] = '1'	Please upload the pdf of the file used	file<br>Custom alignment: LV
	359	pdf2_cc<br><br>Show the field ONLY if:<br>[support_cc] = '1'	Please upload the pdf of the file used	file<br>Custom alignment: LV
	360	pdf3_cc<br><br>Show the field ONLY if:<br>[support_cc] = '1'	Please upload the pdf of the file used	file<br>Custom alignment: LV
	361	annotatedpdf_cc	Please upload the annotated pdf for discussion purposes at the end.	file<br>Custom alignment: LV
	362	quality_scale_casecontrol_complete	Section Header: *Form Status*<br>Complete?	dropdown<br>0 Incomplete<br>1 Unverified<br>2 Complete

Instrument: **Quality Scale Cohort** (quality_scale_cohort) Collapse

	363	qualityscale_cohort	The Newcastle-Ottawa Quality Assessment Scale<br>Cohort Studies<br><br>Note: A study can be awarded a maximum of one star for each numbered item within the Selection and Outcome categories. A maximum of two stars can be given for Comparability.	descriptive
	364	nos_cohort_codername	Coder's last name	text
	365	selection1_cohort	1. Representativeness of the exposed cohort<br><br>Item is assessing the representativeness of exposed individuals in the community, not the representativeness of the sample of women from some general population. For example, subjects derived from groups likely to contain middle class, better educated, health oriented women are likely to be representative of postmenopausal estrogen users while they are not representative of all women (e.g. members of a health maintenance organisation (HMO) will be a representative sample of estrogen users. While the HMO may have an under-representation of ethnic groups, the poor, and poorly educated, these excluded groups are not the predominant users users of estrogen).<br><br>a) truly representative of the average ______________ (describe) in the community =1 star<br><br>b) somewhat representative of the average _____________ in the community =1 star<br><br>c) selected group of users eg nurses, volunteers =0 stars<br><br>d)no description of the derivation of the cohort =0 stars	dropdown<br>0 0 stars<br>1 1 star<br><br>Custom alignment: LV
	366	selection2_cohort	2. Selection of the non exposed cohort a) drawn from the same community as the exposed cohort=1 star b) drawn from a different source=0 stars c)no description of the derivation of the non exposed cohort=0 stars Note: In the case of general population can code=1; if other occupational groups only then code 0.5 stars given possible overlaping exposures	dropdown<br>0 0 stars<br>1 1 star<br><br>Custom alignment: LV

	367	selection3_cohort	3. Ascertainment of exposure a) secure record (eg surgical records) =1 star b) structured interview =1 star c) written self report=0 stars d) No description =0 stars Note: if self-report=zero. Exposure based on registry/death records= 0.5 stars. Disregard other confounders when assessing on this item.	dropdown<br>0 — 0 stars<br>1 — 1 star<br><br>Custom alignment: LV
	368	selection4_cohort	4) Demonstration That Outcome of Interest Was Not Present at Start of Study In the case of mortality studies, outcome of interest is still the presence of a disease/ incident, rather than death. That is to say that a statement of no history of disease or incident earns a star.a) yes =1 star b) no =0 stars Note: For mortlaity studies code=1 if there if you can assume that persons were alive at enrollment into the cohort.	dropdown<br>0 — 0 stars<br>1 — 1 star<br><br>Custom alignment: LV
	369	compare_cohort	Section Header: *COMPARABILITY A maximum of 2 stars can be allotted in this category Either exposed and non-exposed individuals must be matched in the design and/or confounders must be adjusted for in the analysis. Statements of no differences between groups or that differences were not statistically significant are not sufficient for establishing comparability. Note: If the relative risk for the exposure of interest is adjusted for the confounders listed, then the groups will be considered to be comparable on each variable used in the adjustment. There may be multiple ratings for this item for different categories of exposure (e.g. ever vs. never, current vs. previous or never) Age =*, Other controlled factors =**<br><br>1) Comparability of Cohorts on the Basis of the Design or Analysis Age and one other control OR stratified analysis will qualify for two star rating a) study controls for ____AGE___________ (Select the most important factor.) =1 star b) study controls for any additional factor (This criteria could be modified to indicate specific control for a second important factor.) =1 star Note: If analysis adjusts for age (e.g., SMR SIR) then at least one star can applied-- any other adjustment or stratified analysis qualified for an additional star. If the only stratification variable is age then=1.	dropdown<br>0 — 0 stars<br>1 — 1 star<br>2 — 2 stars<br><br>Custom alignment: LV
	370	out1_cohort	Section Header: *OUTCOME*<br><br>1)Assessment of outcome For some outcomes (e.g. fractured hip), reference to the medical record is sufficient tosatisfy the requirement for confirmation of the fracture. This would not be adequatefor vertebral fracture outcomes where reference to x-rays would be required.a) independent blind assessment (Independent or blind assessment stated in the paper, or confirmation of the outcome by reference to secure records (x-rays, medical records, etc.)=1 star b) record linkage (e.g. identified through ICD codes on database records) =1 star c) self report (i.e. no reference to original medical records or x-rays to confirm theoutcome) =0 stars d) no description=0 stars Note: ICD version should be specified in order to earn 1 star.	dropdown<br>0 — 0 stars<br>1 — 1 star<br><br>Custom alignment: LV
	371	out2_cohort	2) Was follow-up long enough for outcomes to occur Must mention >=1 year lag An acceptable length of time should be decided before quality assessment begins (e.g.5 yrs. for exposure to breast implants)a) yes (select an adequate follow up period for outcome of interest) =1 star b) no=0 stars Note: Must mention >=2 year lag; if not then assign=0	dropdown<br>0 — 0 stars<br>1 — 1 star<br><br>Custom alignment: LV
	372	out3_cohort	3)Adequacy of follow up of cohorts This item assesses the follow-up of the exposed and non-exposed cohorts to ensurethat losses are not related to either the exposure or the outcome.a) complete follow up - all subjects accounted for=1 star b) subjects lost to follow up unlikely to introduce bias - small number lost - > ____ % (select anadequate %) follow up, or description provided of those lost)=1 star c) follow up rate < ____% (select an adequate %) and no description of those lost=0 stars d) no statement =0 stars Note: No star assignment if loss to follow-up exceeds 10%; Active follow-up (e.g., last date of contact reported when the event could be verified)=1; passive follow-up or no mention of follow-up=0)	dropdown<br>0 — 0 stars<br>1 — 1 star<br><br>Custom alignment: LV

	373	star_total_cohort	Total # of stars	calc<br>Calculation: ([selection1_cohort]+[selection2_cohort]+[selection3_cohort]+[selection4_cohort]+[compare_cohort]+[out1_cohort]+[out2_cohort]+[out3_cohort])<br>Custom alignment: LV
	374	support	Were any supporting documents referred to in filling out information for this form?	yesno<br>1 Yes<br>0 No<br>Custom alignment: LV
	375	pdf1<br>Show the field ONLY if:<br>[support] = '1'	Please upload the pdf of the file used	file<br>Custom alignment: LV
	376	pdf2<br>Show the field ONLY if:<br>[support] = '1'	Please upload the pdf of the file used	file<br>Custom alignment: LV
	377	pdf3<br>Show the field ONLY if:<br>[support] = '1'	Please upload the pdf of the file used	file<br>Custom alignment: LV
	378	annotatedpdf	Please upload the annotated pdf for discussion purposes at the end.	file<br>Custom alignment: LV
	379	quality_scale_cohort_complete	Section Header: *Form Status*<br>Complete?	dropdown<br>0 Incomplete<br>1 Unverified<br>2 Complete
Instrument: **RTI Quality Assessment** (rti_quality_assessment) Collapse				
	380	rti_codername	Coder's last name	text
	381	rti_time_start	Start Time	text (time)
	382	rti_sampledef	SAMPLE DEFINITION AND SELECTION	descriptive
	383	sampledef_rti1	Retrospective/Prospective1. Is the study design prospective, retrospective, or mixed? [Abstractor: Prospective design requires that the outcome has not occurred at the time the study is initiated and information is collected over time to assess relationships with the outcome (and includes nested case-control studies). Mixed design includes case-control or cohort studies in which one group is studied prospectively and the other retrospectively. A retrospective design analyzes data from past records. The question is not applicable to cross-sectional studies. Note: retrospective cohort designs should be coded as retrospective]	dropdown<br>1 Prospective<br>2 Mixed<br>3 Retrospective<br>4 Cannot determine/not applicable
	384	sampledef_rti1_expl	Retrospective/Prospective1. Is the study design prospective, retrospective, or mixed? EXPLANATION FOR RATING	notes

	385	sampledef_rti2	Inclusion/Exclusion Criteria2. Are critical inclusion/exclusion criteria clearly stated (does not require the reader to infer)? [Principal Investigator (PI): Provide direction to abstractors by listing individual criteria of a priori significance and minimal requirements for criteria to be considered “clearly stated.” Include this question to identify specific inclusion/exclusion criteria that should be consistently recorded across studies] [Abstractor: Use “Partially” if only some criteria are stated or if some criteria are not clearly stated (corresponding to directions provided by the PI). Note that studies may describe inclusion criteria alone (i.e., include x), exclusion criteria (i.e., do not include x), or a combination of inclusion and exclusion criteria. Note: many studies will describe these criteria on the basis of identifying firefighters thru employment/ certification/death certificate coding records which can be classified as ‘yes’]	dropdown<br>1 Yes<br>2 Partially: some, but not all, criteria stated or some criteria not clearly stated<br>3 No
	386	sampledef_rti2_expl	Inclusion/Exclusion Criteria2. Are critical inclusion/exclusion criteria clearly stated (does not require the reader to infer)? EXPLANATION FOR RATING	notes
	387	sampledef_rti3	Inclusion/Exclusion Criteria3. Are the inclusion/exclusion criteria measured using valid and reliable measures? [PI: Separately specify each criterion that abstractors should consider based on its relevance to study bias. It is unlikely that all criteria will need to be evaluated in relation to this question. Provide direction to abstractors on valid and reliable measurement of each criterion that is to be considered. For example, prior exposure or disease status is a frequent inclusion/exclusion criterion, particularly in inception cohorts. Subjective measures based on self-report tend to have lower reliability and validity than objective measures such as clinical reports and lab findings. Replicate question to evaluate each individual inclusion/exclusion criterion. Note, in most cases firefighter studies will be coded yes since most studies rely upon administrative/employment/ death certificate records to identify firefighters for inclusion in the study.]	dropdown<br>1 Yes<br>2 No<br>3 Cannot determine; measurement approach not reported
	388	sampledef_rti3_expl	Inclusion/Exclusion Criteria 3. Are the inclusion/exclusion criteria measured using valid and reliable measures? EXPLANATION FOR RATING	notes
	389	sampledef_rti4	Inclusion/Exclusion Criteria 4. Did the study apply inclusion/exclusion criteria uniformly to all comparison groups/arms of the study? [PI: Drop question if not relevant to entire body of evidence (e.g., all case-series, single- arm studies). Note: it may be possible that criteria are not uniformly applied when assembling firefighter cohorts or comparison groups]	dropdown<br>1 Yes<br>2 Partially: some, but not all criteria, applied to all arms or not clearly stated if some criteria are applied to all arms<br>3 No<br>4 Cannot determine: article does not specify<br>5 Not applicable: study has only one arm and so does not include comparison groups
	390	sampledef_rti4_expl	Inclusion/Exclusion Criteria4. Did the study apply inclusion/exclusion criteria uniformly to all comparison groups/arms of the study? EXPLANATION FOR RATING	notes
	391	sampledef_rti5	Inclusion/Exclusion Criteria 5. Was the strategy for recruiting participants into the study the same across study groups/arms of the study? [PIs: This question is likely to be more relevant for prospective or mixed designs than retrospective designs. Drop question if not relevant to entire body of evidence (e.g., all studies generally have only one arm). Note: true recruitment into a cohort study is rare for firefighter cancer studies so most studies can be judged as meeting this criteria. In most cases for case-control studies this will be coded as “yes”, unless case and control recruitment differs.]	dropdown<br>1 Yes<br>2 No<br>3 Cannot determine<br>4 Not applicable: one study group/arm

	392	sampledef_rti5_expl	Inclusion/Exclusion Criteria5. Was the strategy for recruiting participants into the study the same across study groups/arms of the study? EXPLANATION FOR RATING	notes
	393	excluded_rti6	EXCLUDED: 6. Was the sample size sufficiently large to detect a clinically significant difference of 5% or more between groups in at least one primary outcome measure?	descriptive
	394	rti_interv_exp	INTERVENTIONS/EXPOSURE	descriptive
	395	interv_exp_rti7	Clear Specification7. What is the level of detail in describing the intervention or exposure? [PI: Specify which details need to be stated (e.g., intensity, duration, frequency, route, setting, and timing of intervention/exposure). For case-control studies, consider whether the condition, timing, frequency, and setting of symptoms are provided in the case definition. PI needs to establish criteria for high, medium, or low response. Note: Many firefighter exposures are often based on occupational title/certification/hospital-based record or death certificate which which can be judged as medium given that it is a crude measure of exposure. Important to consider if the comparison group is truly unexposed or reasonably complete in order to mark as high]	dropdown<br>1 High: very clear, all PI-required details provided<br>2 Medium: somewhat clear, majority of PI-required details provided<br>3 Low: unclear, many PI-required details missing
	396	interv_exp_rti7_expl	Clear Specification7. What is the level of detail in describing the intervention or exposure? EXPLANATION FOR RATING	notes
	397	excluded_rti8	EXCLUDED: OUTCOMES: Clear Specification 8. Are the important outcomes pre-specified by the researchers?	descriptive
	398	rti_tx_grps	CREATION OF TREATMENT GROUPS	descriptive
	399	tx_grps_rti9	Allocation9. Is the selection of the comparison group appropriate, after taking into account feasibility and ethical considerations. [PI: Provide instruction to the abstractor based on the type of study. Interventions with community components are likely to have contamination if all groups are drawn from the same community. Interventions without community components should select groups from the same source (e.g., community or hospital) to reduce baseline differences across groups. For case-control studies, controls should represent the population from which cases arose; that is, controls should have met the case definition if they had the outcome. Note: For most firefighter cohort studies the comparison group will be non-fighters drawn from the same registry while case control studies will draw controls from the same hospital system(s) or surrounding communities. In most cases these can be rated as “yes”. Cases in which selected controls clearly do not reflect the same community in cohort studies can be rated as “no” (e.g., comparing deaths in firefighters in a particular state to nation-wide mortality). In cases where the time periods or exposure and disease are different across groups in comparison, rate as “no”. In cases when multiple comparators/controls are applied select and rate the most favorable case (i.e., if at least one assessment is “yes” then report “yes for this item).]	dropdown<br>1 Yes<br>2 No<br>3 Cannot determine or no description of the derivation of the comparison group<br>4 Not applicable: study does not include a comparison group (case series, one study arm)
	400	tx_grps_rti9_expl	Allocation9. Is the selection of the comparison group appropriate, after taking into account feasibility and ethical considerations. EXPLANATION FOR RATING	notes
	401	tx_grps_rti10	Any Attempt to Balance10. Any attempt to balance the allocation between the groups (e.g., through stratification, matching, propensity scores). [PI: This is most likely to be used in case-control study designs. Drop if not relevant to the body of evidence. Note: Score all prospective studies or retrospective cohorts as “Not applicable”; for case-control studies that employ any of these in the design phase—not the analytic phase]	dropdown<br>1 Yes or study accounts for imbalance between groups through a post hoc approach such as multivariate analysis<br>2 No or cannot determine<br>3 Not applicable: study does not include a comparison group (case series or one study arm)

	402	tx_grps_rti10_expl	Any Attempt to Balance10. Any attempt to balance the allocation between the groups (e.g., through stratification, matching, propensity scores). EXPLANATION FOR RATING	notes
	403	excluded_rti11	EXCLUDED: Contamination 11. Did researchers isolate the impact from a concurrent intervention or an unintended exposure that might bias results, e.g., through multivariate analysis, stratification, or subgroup analysis?	descriptive
	404	excluded_rti12	EXCLUDED: Contamination 12. Did execution of the study vary from the intervention protocol proposed by the investigators and therefore compromise the conclusions of the study?	descriptive
	405	excluded_rti13	EXCLUDED: Blind Outcomes Assessment 13. Were the outcome assessors blinded to the intervention or exposure status of participants?	descriptive
	406	rti_soundness	SOUNDNESS OF INFORMATION	descriptive
	407	soundness_rti14	Source of Information For Interventions/Exposure14. Are interventions/exposures assessed using valid and reliable measures, implemented consistently across all study participants? [PI: Important measures may be listed separately. PI may need to establish a threshold for what would constitute acceptable measures based on study topic. When subjective or objective measures could be collected, subjective measures based on self- report may be considered as being less reliable and valid than objective measures such as clinical reports and lab findings. Replicate question when needed. Note: Code High if there is a job title certification. If death certificate or registry records code medium, if no specification code Low.	dropdown<br>1 High<br>2 Medium<br>3 Low
	408	soundness_rti14_expl	Source of Information For Interventions/Exposure14. Are interventions/exposures assessed using valid and reliable measures, implemented consistently across all study participants? [PI: Important measures may be listed separately. EXPLANATION FOR RATING	notes
	409	soundness_rti15	Source of Information for Outcomes15. Are outcomes assessed using valid and reliable measures, implemented consistently across all study participants? [PI: Primary outcomes should be identified for abstractors and if there is more than one, they may be listed separately. Also, identify any relevant secondary outcomes and harms. Subjective measures based on self-report tend to have lower reliability and validity than objective measures such as clinical reports and lab findings. Note for case-control studies: consider whether the ascertainment of cases was independent of exposure. Note: In most cases this will be coded “yes” since outcomes are typically derived registry/death certificate records and are collected in the same manner across exposure groups. In rare cases this information may not be collected in the same manner across exposure groups in which a “no” rating would be appropriate. Additional rare events could include poorly specified sources of incidence or mortality events which would warrant “Cannot determine….]	dropdown<br>1 Yes<br>2 No<br>3 Cannot determine or measurement approach not reported
	410	soundness_rti15_expl	Source of Information for Outcomes15. Are outcomes assessed using valid and reliable measures, implemented consistently across all study participants? EXPLANATION FOR RATING	notes
	411	rti_followup	FOLLOW-UP	descriptive
	412	followup_rti16	EXCLUDED: Equality of Length of Follow-Up for Participants 16. Is the length of follow-up the same for all groups?	descriptive

	413	followup_rti17	Length of Follow-Up Adequate17. Is the length of time following the intervention/exposure sufficient to support the evaluation of primary outcomes and harms? [PI: Primary outcomes (including harms) should be identified for abstractors. Important measures may be listed separately. Abstractors should be provided with specific criteria for sufficient length of follow-up based on prior research or theory. Drop if entire body of evidence is cross-sectional or if minimal length of follow-up period is specified through inclusion criteria. Note: If cross-sectional list non-applicable. Otherwise in most cases this will be coded "yes" unless the follow-up period is less than 5 years then code "No". If there is no information provided on the follow-up period then report "Cannot determine"]	dropdown<br>1 Yes<br>2 Partially: some primary outcomes are followed for a sufficient length of time<br>3 No<br>4 Cannot determine<br>5 Not applicable: cross-sectional
	414	followup_rti17_expl	Length of Follow-Up Adequate17. Is the length of time following the intervention/exposure sufficient to support the evaluation of primary outcomes and harms? EXPLANATION FOR RATING	notes
	415	excluded_rti18	EXCLUDED: Completeness of Follow-Up 18. Did attrition from any group exceed [x] percent?	descriptive
	416	excluded_rti19	EXCLUDED: Completeness of Follow-Up 19. Did attrition differ between groups by more than 20 percent?	descriptive
	417	rti_anal_comp	ANALYSIS COMPARABILITY	descriptive
	418	excluded_rti20	EXCLUDED: Assessment of Baseline Comparability 20. Does the analysis control for baseline differences between groups?	descriptive
	419	excluded_rti21	EXCLUDED: Identification of Prognostic Factors (Effect Modifiers and Confounders) 21. Are confounding and/or effect modifying variables assessed using valid and reliable measures across all study participants?	descriptive
	420	anal_comp_rti22	Case-Mix Adjustment22. Were the important confounding and effect modifying variables taken into account in the design and/or analysis (e.g., through matching, stratification, interaction terms, multivariate analysis, or other statistical adjustment)? [PI: Provide instruction to abstractors on adequate adjustment for confounding and testing for effect modification. Note: if only age is accounted for in the analysis or via age group stratification, then select "partially"; select "yes if two or more variables is accounted for in the analysis or via age group stratification; otherwise select "no"]	dropdown<br>1 Yes<br>2 Partially: some variables taken into account or adjustment achieved to some extent<br>3 No: not accounted for or not identified<br>4 Cannot determine
	421	anal_comp_rti22_expl	Case-Mix Adjustment22. Were the important confounding and effect modifying variables taken into account in the design and/or analysis (e.g., through matching, stratification, interaction terms, multivariate analysis, or other statistical adjustment)? EXPLANATION FOR RATING	notes
	422	rti_anal_outcome	ANALYSIS OUTCOME	descriptive
	423	excluded_rti23	EXCLUDED: Intention-to-Treat Analysis 23. In cases of high loss to follow-up (or differential loss to follow-up), is the impact assessed (e.g., through sensitivity analysis or other adjustment method)?	descriptive
	424	excluded_rti24	EXCLUDED: Appropriate Analytic Methods 24. Are any important primary outcomes missing from the results?	descriptive
	425	excluded_rti25	EXCLUDED: Appropriate Analytic Methods: 25. Are the statistical methods used to assess the primary benefit outcomes appropriate to the data?	descriptive
	426	excluded_rti26	EXCLUDED: Appropriate Analytic Methods 26. Are any important harms or adverse events that may be a consequence of the intervention/exposure missing from the results?	descriptive
	427	excluded_rti27	EXCLUDED: Appropriate Analytic Methods 27. Are the statistical methods used to assess the main harm or adverse event outcomes appropriate to the data?	descriptive
	428	rti_interp	INTERPRETATION	descriptive

	429	interp_rti28	Appropriately Based on Results28. Are results believable taking study limitations into consideration? [Abstractor:This question is intended to capture the overall quality of the study. Consider issues that may limit your ability to interpret the results of the study. Review responses to earlier questions for specific criteria. Note: Most firefighter linkage studies have inherent limitations such as job title as proxy for exposures that limit study 'believability'. These studies and those employing case-control designs can also be impacted by limited statistical power. Coding of "partially" and "no" should be reserved for studies that have limitations beyond those that broadly impact firefighter cancer studies.]	dropdown<br>1 Yes<br>2 Partially<br>3 No
	430	interp_rti28_expl	Appropriately Based on Results28. Are results believable taking study limitations into consideration? EXPLANATION FOR RATING	notes
	431	rti_pres_rep	PRESENTATION AND REPORTING	descriptive
	432	pres_rep_rti29	Completeness, Clarity, and Structure29. Is the source of funding identified? [PI: The relevance of this question will depend upon the topic. This question may be modified to identify particular sources of funding (e.g., industry, government, university, or foundation funding).] If it is acknowledged that there is no source of external funding then code "Yes".	dropdown<br>1 Yes<br>2 No
	433	pres_rep_rti29_expl	Completeness, Clarity, and Structure29. Is the source of funding identified? EXPLANATON FOR RATING	notes
	434	rti_time_end	End Time	text (time)
	435	rti_quality_assessment_complete	Section Header: *Form Status*<br>Complete?	dropdown<br>0 Incomplete<br>1 Unverified<br>2 Complete

Instrument: **Newcastle Case-Control** (newcastle_casecontrol) Collapse

	436	qualityscale_v2	The Newcastle-Ottawa Quality Assessment Scale<br>Case Control Studies<br><br>Note: A study can be awarded a maximum of one star for each numbered item within the Selection and Exposure categories. A maximum of two stars can be given for Comparability.	descriptive
	437	nos_ca_co_codername_v2	Coder's last name	text
	438	newcas_cohort_start_time	Start Time	text (time)
	439	selection1_cc_v2	Section Header: *SELECTION*<br>1. Is the Case Definition Adequate? a) yes, with independent validation (Requires some independent validation (e.g. >1 person/record/time/process to extract information, or reference to primary record source such as x-rays or medical/hospital records)) =1 star b) yes, eg record linkage or based on self reports (Record linkage (e.g. ICD codes in database) or self-report with no reference to primary record) =0 stars c) No description =0 stars Note: ICD coding version should be specified in order to be awarded one star	dropdown<br>0 0 stars<br>1 1 star<br><br>Custom alignment: LV
	440	selection2_cc_v2	2. Representativeness of the Cases<br><br>a) consecutive or obviously representative series of cases (All eligible cases with outcome of interest over a defined period of time, all cases in a defined catchment area, all cases in a defined hospital or clinic, group of hospitals, health maintenance organisation, or an appropriate sample of those cases (e.g. random sample)) =1 star<br><br>b) potential for selection biases or not stated (Not satisfying requirements in part (a), or not stated.) =0 stars	dropdown<br>0 0 stars<br>1 1 star<br><br>Custom alignment: LV

	441	selection3_cc_v2	3. Selection of Controls<br><br>This item assesses whether the control series used in the study is derived from the same population as the cases and essentially would have been cases had the outcome been present.<br><br>a) community controls (i.e. same community as cases and would be cases if had outcome)) =1 star<br><br>b) hospital controls (within same community as cases (i.e. not another city) but derived from a hospitalised population) =0 stars<br><br>c) No description =0 stars	dropdown<br>0 | 0 stars<br>1 | 1 star<br><br>Custom alignment: LV
	442	selection4_cc_v2	4) Definition of Controls<br><br>a) no history of diesease (endpoint) (If cases are first occurrence of outcome, then it must explicitly state that controls have no history of this outcome. If cases have new (not necessarily first) occurrence of outcome, then controls with previous occurrences of outcome of interest should not be excluded.) =1 star<br><br>b) no description of source (No mention of history of outcome) =0 stars	dropdown<br>0 | 0 stars<br>1 | 1 star<br><br>Custom alignment: LV
	443	compare_cc_v2	Section Header: *COMPARABILITY A maximum of 2 stars can be allotted in this category Either cases and controls must be matched in the design and/or confounders must be adjusted for in the analysis. Statements of no differences between groups or that differences were not statistically significant are not sufficient for establishing comparability. Note: If the odds ratio for the exposure of interest is adjusted for the confounders listed, then the groups will be considered to be comparable on each variable used in the adjustment. There may be multiple ratings for this item for different categories of exposure (e.g. ever vs. never, current vs. previous or never) Age =*, Other controlled factors =**<br><br>1) Comparability of Cases and Controls on the Basis of the Design or Analysis a) study controls for _______________ (Select the most important factor.) =1 star b) study controls for any additional factor (This criteria could be modified to indicate specific control for a second important factor.) =1 star Note: age only =1, age+any other variable=2	dropdown<br>0 | 0 stars<br>1 | 1 star<br>2 | 2 stars<br><br>Custom alignment: LV
	444	exp1_cc_v2	Section Header: *EXPOSURE*<br><br>1) Ascertainment of exposure a) secure record (eg surgical records)=1 star b) structured interview where blind to case/control status =1 star c) interview not blinded to case/control status=0 stars d) written self report or medical record only=0 stars e) no description=0 stars Note: Can ignore "blind to case/control status" and code all structured interviews as=1.	dropdown<br>0 | 0 stars<br>1 | 1 star<br><br>Custom alignment: LV
	445	exp2_cc_v2	2) Same method of ascertainment for cases and controls a) yes=1 star b) no=0 stars Note: In the case of occupational/certification determination of occupational status applied only to firefighters then code=0.	dropdown<br>0 | 0 stars<br>1 | 1 star<br><br>Custom alignment: LV
	446	exp3_cc_v2	(DO NOT CODE THIS ITEM) 3) Non-Response rate a) same rate for both groups=1 star b) non respondents described=0 stars c) rate different and no designation=0 stars	dropdown<br>0 | 0 stars<br>1 | 1 star<br><br>Custom alignment: LV
	447	star_total_cc_v2	Total # of stars	calc<br>Calculation: ([selection1_cc_v2]+ [selection2_cc_v2]+ [selection3_cc_v2]+ [selection4_cc_v2]+ [compare_cc_v2]+[exp1_cc_v2]+[exp2_cc_v2]+ [exp3_cc_v2])<br>Custom alignment: LV

	448	support_cc_v2	Were any supporting documents referred to in filling out information for this form?	yesno<br>1 Yes<br>0 No<br><br>Custom alignment: LV
	449	pdf1_cc_v2<br><br>Show the field ONLY if:<br>[support_cc_v2] = '1'	Please upload the pdf of the file used	file<br>Custom alignment: LV
	450	pdf2_cc_v2<br><br>Show the field ONLY if:<br>[support_cc_v2] = '1'	Please upload the pdf of the file used	file<br>Custom alignment: LV
	451	pdf3_cc_v2<br><br>Show the field ONLY if:<br>[support_cc_v2] = '1'	Please upload the pdf of the file used	file<br>Custom alignment: LV
	452	annotatedpdf_cc_v2	Please upload the annotated pdf for discussion purposes at the end.	file<br>Custom alignment: LV
	453	newcas_cohort_end_time	End Time	text (time)
	454	newcastle_casecontrol_complete	Section Header: *Form Status*<br>Complete?	dropdown<br>0 Incomplete<br>1 Unverified<br>2 Complete

Instrument: **Newcastle Cohort** (newcastle_cohort) Collapse

	455	qualityscale_cohort_v2	The Newcastle-Ottawa Quality Assessment Scale<br>Cohort Studies<br><br>Note: A study can be awarded a maximum of one star for each numbered item within the Selection and Outcome categories. A maximum of two stars can be given for Comparability.	descriptive
	456	nos_cohort_codername_v2	Coder's last name	text
	457	newcas_caco_start_time	Start Time	text (time)
	458	selection1_cohort_v2	1. Representativeness of the exposed cohort<br><br>Item is assessing the representativeness of exposed individuals in the community, not the representativeness of the sample of women from some general population. For example, subjects derived from groups likely to contain middle class, better educated, health oriented women are likely to be representative of postmenopausal estrogen users while they are not representative of all women (e.g. members of a health maintenance organisation (HMO) will be a representative sample of estrogen users. While the HMO may have an under-representation of ethnic groups, the poor, and poorly educated, these excluded groups are not the predominant users users of estrogen).<br><br>a) truly representative of the average _______________ (describe) in the community =1 star<br><br>b) somewhat representative of the average ______________ in the community =1 star<br><br>c) selected group of users eg nurses, volunteers =0 stars<br><br>d)no description of the derivation of the cohort =0 stars	dropdown<br>0 0 stars<br>1 1 star<br><br>Custom alignment: LV

	459	selection2_cohort_v2	2. Selection of the non exposed cohort a) drawn from the same community as the exposed cohort=1 star b) drawn from a different source=0 stars c)no description of the derivation of the non exposed cohort=0 stars Note: In the case of general population can code=1; if other occupational groups only then code 0.5 stars given possible overlaping exposures	dropdown<br>0 | 0 stars<br>0.5 | 0.5 star<br>1 | 1 star<br><br>Custom alignment: LV
	460	selection3_cohort_v2	3. Ascertainment of exposure a) secure record (eg surgical records) =1 star b) structured interview =1 star c) written self report=0 stars d) No description =0 stars Note: if self-report=zero. Exposure based on registry/death records= 0.5 stars. Disregard other confounders when assessing on this item.	dropdown<br>0 | 0 stars<br>0.5 | 0.5 star<br>1 | 1 star<br><br>Custom alignment: LV
	461	selection4_cohort_v2	4) Demonstration That Outcome of Interest Was Not Present at Start of Study In the case of mortality studies, outcome of interest is still the presence of a disease/ incident, rather than death. That is to say that a statement of no history of disease or incident earns a star.a) yes =1 star b) no =0 stars Note: For mortlaity studies code=1 if there if you can assume that persons were alive at enrollment into the cohort.	dropdown<br>0 | 0 stars<br>1 | 1 star<br><br>Custom alignment: LV
	462	compare_cohort_v2	Section Header: *COMPARABILITY A maximum of 2 stars can be allotted in this category Either exposed and non-exposed individuals must be matched in the design and/or confounders must be adjusted for in the analysis. Statements of no differences between groups or that differences were not statistically significant are not sufficient for establishing comparability. Note: If the relative risk for the exposure of interest is adjusted for the confounders listed, then the groups will be considered to be comparable on each variable used in the adjustment. There may be multiple ratings for this item for different categories of exposure (e.g. ever vs. never, current vs. previous or never) Age =*, Other controlled factors =**<br><br>1) Comparability of Cohorts on the Basis of the Design or Analysis Age and one other control OR stratified analysis will qualify for two star rating a) study controls for ____AGE___________ (Select the most important factor.) =1 star b) study controls for any additional factor (This criteria could be modified to indicate specific control for a second important factor.) =1 star Note: If analysis adjusts for age (e.g., SMR SIR) then at least one star can applied-- any other adjustment or stratified analysis qualified for an additional star. If the only stratification variable is age then=1.	dropdown<br>0 | 0 stars<br>1 | 1 star<br>2 | 2 stars<br><br>Custom alignment: LV
	463	out1_cohort_v2	Section Header: *OUTCOME*<br><br>1)Assessment of outcome For some outcomes (e.g. fractured hip), reference to the medical record is sufficient tosatisfy the requirement for confirmation of the fracture. This would not be adequatefor vertebral fracture outcomes where reference to x-rays would be required.a) independent blind assessment (Independent or blind assessment stated in the paper, or confirmation of the outcome by reference to secure records (x-rays, medical records, etc.)=1 star b) record linkage (e.g. identified through ICD codes on database records) =1 star c) self report (i.e. no reference to original medical records or x-rays to confirm theoutcome) =0 stars d) no description=0 stars Note: ICD version should be specified in order to earn 1 star.	dropdown<br>0 | 0 stars<br>1 | 1 star<br><br>Custom alignment: LV
	464	out2_cohort_v2	2) Was follow-up long enough for outcomes to occur a) yes (select an adequate follow up period for outcome of interest) =1 star b) no=0 stars Note: If average follow-up is 5+years=1; if not then assign=0	dropdown<br>0 | 0 stars<br>1 | 1 star<br><br>Custom alignment: LV

	465	out3_cohort_v2	3)Adequacy of follow up of cohorts This item assesses the follow-up of the exposed and non-exposed cohorts to ensurethat losses are not related to either the exposure or the outcome.a) complete follow up - all subjects accounted for=1 star b) subjects lost to follow up unlikely to introduce bias - small number lost - > ____ % (select anadequate %) follow up, or description provided of those lost)=1 star c) follow up rate < ____% (select an adequate %) and no description of those lost=0 stars d) no statement =0 stars Note: No star assignment if loss to follow-up exceeds 10%; Active follow-up (e.g., last date of contact reported when the event could be verified) in both groups=1; passive follow-up in all groups=0.5 stars; no mention of follow-up=0)	dropdown<br>0 | 0 stars<br>0.5 | 0.5 stars<br>1 | 1 star<br><br>Custom alignment: LV
	466	star_total_cohort_v2	Total # of stars	calc<br>Calculation: ([selection1_cohort_v2]+ [selection2_cohort_v2]+ [selection3_cohort_v2]+ [selection4_cohort_v2]+[compare_cohort_v2]+ [out1_cohort_v2]+[out2_cohort_v2]+[out3_cohort_v2])<br>Custom alignment: LV
	467	support_v2	Were any supporting documents referred to in filling out information for this form?	yesno<br>1 | Yes<br>0 | No<br><br>Custom alignment: LV
	468	pdf1_v2<br><br>Show the field ONLY if:<br>[support_v2] = '1'	Please upload the pdf of the file used	file<br>Custom alignment: LV
	469	pdf2_v2<br><br>Show the field ONLY if:<br>[support_v2] = '1'	Please upload the pdf of the file used	file<br>Custom alignment: LV
	470	pdf3_v2<br><br>Show the field ONLY if:<br>[support_v2] = '1'	Please upload the pdf of the file used	file<br>Custom alignment: LV
	471	annotatedpdf_v2	Please upload the annotated pdf for discussion purposes at the end.	file<br>Custom alignment: LV
	472	newcas_caco_end_time	End Time	text (time)
	473	newcastle_cohort_complete	Section Header: *Form Status*<br>Complete?	dropdown<br>0 | Incomplete<br>1 | Unverified<br>2 | Complete
